# Molecular regulation and physiological role of GOLPH3-mediated Golgi retention

**DOI:** 10.1038/s41467-026-74133-6

**Published:** 2026-06-11

**Authors:** Anastasia Theodoropoulou, Anita Nasrallah, Luciano A. Abriata, Laurence Abrami, Juliane Da Graça, Irmak Kaysudu, Francesco Talotta, Maria J. Marcaida, Muhammad U. Anwar, Ondrej Kováč, Sergey Y. Vakhrushev, Alejandro Alonso-Calleja, Sylvia Ho, Antonino Asaro, Francisco S. Mesquita, Leila Alieh, Charlotte Gehin, Lucie Bracq, Nika Goršek, Sarah Vacle, Arthur Samurkas, Alessio Prunotto, Luca Fusar Bassini, Miroslav Machala, Olaia Naveiras, Katrine T. Schjoldager, F. Gisou van der Goot, Matteo Dal Peraro, Giovanni D’Angelo

**Affiliations:** 1 Laboratory for Biomolecular Modeling, Institute of Bioengineering, School of Life Sciences, École Polytechnique Fédérale de Lausanne (EPFL), Lausanne, Switzerland; 2 Laboratory of Lipid Cell Biology, Institute of Bioengineering and Global Health Institute, School of Life Sciences, École Polytechnique Fédérale de Lausanne (EPFL), Lausanne, Switzerland; 3 Global Health Institute, School of Life Sciences, EPFL, Lausanne, Switzerland; 4 Institute of Genetics and Biophysics, National Research Council, Naples, Italy; 5 Department of Pharmacology and Toxicology, Veterinary Research Institute, Brno, Czech Republic; 6 Copenhagen Center for Glycocalyx Research, Departments of Cellular and Molecular Medicine, University of Copenhagen, Copenhagen, Denmark; 7 Laboratory of Regenerative Hematopoiesis, Department of Biomedical Sciences, Faculty of Biology and Medicine, University of Lausanne, Lausanne, Switzerland; 8 Laboratory of Brain Development and Biological Data Science, Brain Mind Institute, Faculty of Life Sciences, École Polytechnique Fédérale de Lausanne (EPFL), Lausanne, Switzerland; 9 Hematology Service, Departments of Oncology and Laboratory Medicine, Lausanne University Hospital (CHUV), Lausanne, Switzerland

**Keywords:** Structural biology, Membrane trafficking, Developmental biology, Glycobiology

## Abstract

The Golgi complex serves as the central hub of the biosynthetic pathway, where anterograde and retrograde trafficking converge. How cargo and Golgi-resident proteins traverse this organelle has long been debated. Recent studies have identified a molecular machinery that sorts resident proteins into retrograde-directed COPI vesicles during cisternal maturation. Golgi phosphoprotein 3 (GOLPH3) is a key component of this system; however, its physiological relevance and regulatory mechanisms remain poorly defined. Here, we show that GOLPH3 depletion in mice alters both protein and lipid glycosylation, causes partially penetrant embryonic lethality, and severely impairs growth and bone mineralization. At the molecular level, we find that GOLPH3 is regulated by functionally antagonistic S-acylation events that control the topology of its membrane association. To mediate retrograde trafficking of Golgi-resident glycosyltransferases, GOLPH3 must bind their cytosolic tails. This occurs via a negatively charged surface region, which is correctly oriented only in one of the S-acylated GOLPH3 conformations. Together, these findings reveal a lipid-mediated regulatory mechanism for intra-Golgi trafficking and establish the critical role of GOLPH3 in vertebrate development.

## Abstract (web summary)

Researchers show that the COPI adapter GOLPH3 uses dynamic S-acylation to switch its orientation and control enzyme recycling in the Golgi. This lipid-mediated mechanism is vital for proper glycosylation, vertebrate growth, and bone mineralization.

## Introduction

Proteins and lipids synthesized in the endoplasmic reticulum (ER) are transported to the Golgi complex, where resident enzymes modify them prior to their delivery to final cellular destinations^1^. The most prominent of these modifications is glycosylation, catalyzed by approximately 200 glycosyltransferases that collectively define the mammalian glycomes^2^. Unlike protein or nucleic acid synthesis, glycan assembly proceeds without relying on a nucleic acid template^3^. Nevertheless, it is both highly reproducible and dynamically responsive. The mechanisms that enable this balance of fidelity and flexibility remain only partially understood.

The Golgi apparatus is thought to form from ER-derived carriers that fuse at its *cis* face to generate new *cisternae*. These *cisternae* then mature into medial and *trans* compartments through the sequential remodeling of their glycosyltransferase content, while cargo remains enclosed within the lumen or membrane^4^. Glycosyltransferases are thus continuously recycled within the maturing Golgi stack to support the ordered assembly of glycans on proteins and lipids^5^.

Glycosyltransferase recycling depends on retrograde retrieval via Coat Protein complex 1 (COPI)-coated vesicles, which return enzymes to earlier Golgi compartments. Sorting into these vesicles requires either direct interaction with COPI subunits^6^ or indirect recruitment through adapter proteins that link enzymes to the COPI machinery^7,8^. Although the molecular mechanisms of this sorting process are well characterized, its physiological regulation and broader impact remain poorly defined. In particular, it is unclear how this trafficking system adapts to modulate the glycome in response to cellular cues.

To address this gap, we investigated Golgi phosphoprotein 3 (GOLPH3), the first identified COPI adapter^9–11^. GOLPH3 is a peripheral membrane protein that localizes to the *trans*-Golgi by binding phosphatidylinositol-4-phosphate (PtdIns(4)*P*)^12,13^. Adjacent to its PtdIns(4)*P*-binding site is a hydrophobic β-hairpin that inserts into the membrane and promotes curvature^12,14,15^. Once membrane-bound, GOLPH3 interacts with COPI coatomer via an N-terminal basic motif^9^, and with a large subset of glycosyltransferases, facilitating their retrograde transport and protecting them from lysosomal degradation^10,11^. GOLPH3 preferentially recognizes a basic residue–enriched motif in the cytosolic tails of glycosyltransferases^9–11^, which are typically type II transmembrane proteins with luminal catalytic domains^16^.

GOLPH3 clients span multiple glycosylation pathways, including glycosphingolipid biosynthesis (B4GALT5, ST3GAL5, A4GALT, ST8SIA1 and B4GALNT1)^10^, N-linked (MAN1B1 and MGAT2) and mucin-type O-linked (C1GALT1, GALNT2, GALNT6, GALNT7 and GCNT1) glycosylation, proteoglycan synthesis (EXT1 and FAM20B), O-mannosylation (POMGNT1), tyrosine sulfation (TPST1 and TPST2), and nucleotide hydrolysis^11^. GOLPH3 also maintains lysosomal function by stabilizing LYSET, a key component of the mannose-6-phosphate tagging system^17^. Accordingly, perturbing GOLPH3 expression widely alters both protein and lipid glycosylation in cultured cells^10,11^.

Among its clients, GOLPH3 critically regulates lactosylceramide synthase (LCS, also known as B4GALT5), a *trans*-Golgi enzyme involved in glycosphingolipid biosynthesis^10^. GOLPH3 anchors LCS at the Golgi, shielding it from proteolytic degradation^10^. LCS receives its substrate, glucosylceramide, from the lipid transfer protein FAPP2, another PtdIns(4)*P* effector, and catalyzes the production of complex glycosphingolipids^18,19^. By stabilizing LCS, GOLPH3 plays a key role in lipid remodeling at the Golgi. GOLPH3 also regulates mucin-type O-glycosylation by retaining GALNT family enzymes, which initiate glycan attachment to serine and threonine residues^20^. Loss of GOLPH3, in cellular systems, leads to degradation or mislocalization of these enzymes, impairing glycoprotein processing, disrupting glycan biosynthesis, and compromising extracellular matrix organization^11^.

In this study, we examined the physiological consequences of GOLPH3 dysfunction in a mammalian model and uncovered an essential role in development. We next investigated how cells regulate GOLPH3 membrane association and client interactions. Our results reveal a regulatory mechanism involving antagonistic S-acylation events that establish distinct membrane topologies, either enabling or inhibiting client engagement. These findings demonstrate that GOLPH3-mediated retention of glycosyltransferases can be dynamically modulated by a previously unrecognized mechanism of intra-Golgi trafficking control. This work raises new questions about how acute and post-transcriptional regulatory pathways converge to reshape the glycome, and guide organismal development.

## Results

### GOLPH3 loss of function induces glycosylation defects in vivo

**Fig. 1:**
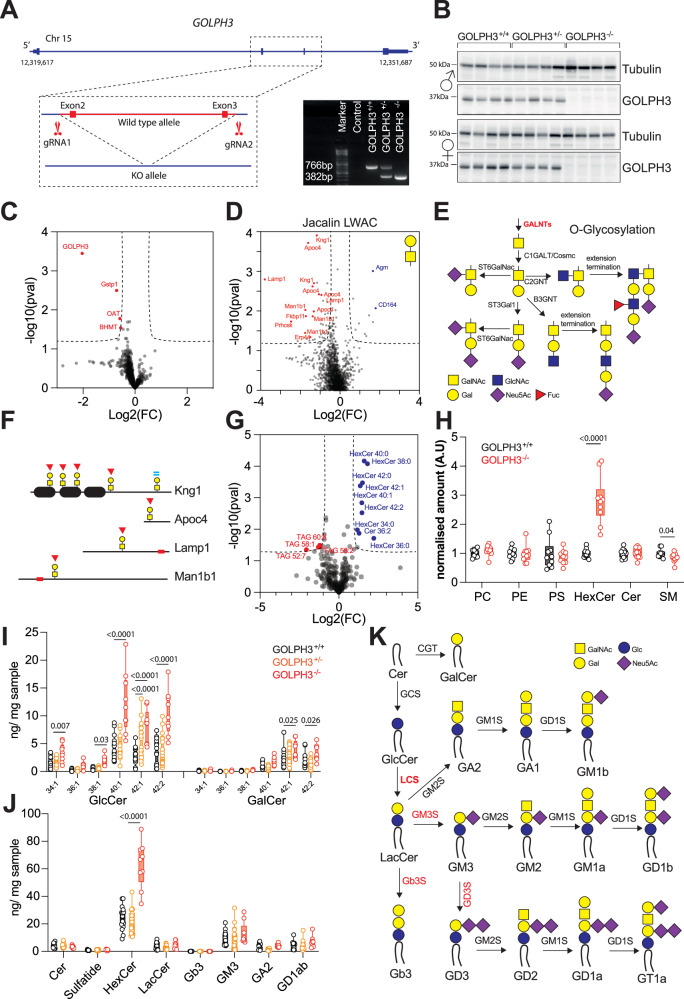
GOLPH3 loss alters glycosylation and lipid metabolism in mouse liver. **A** Schematic of the CRISPR/Cas9-mediated strategy used to generate GOLPH3 knockout (GOLPH3^−/−^) mice by deleting exons 2 and 3. The diagnostic PCR (right) confirms wild-type (766 bp) and KO (382 bp) alleles in genomic DNA from mice of the indicated genotypes. **B** Western blot analysis of liver lysates from male (top) and female (bottom) mice with the indicated genotypes. GOLPH3 and Tubulin (loading control) were detected with specific antibodies, confirming complete loss of GOLPH3 protein in knockout livers. **C** Volcano plot showing differential protein abundance in livers from GOLPH3^+/+^ vs GOLPH3^−/−^ mice (*n* = 3) by TMT-based quantitative proteomics. In red are proteins significantly decreased in GOLPH3^−/−^ livers. GOLPH3 is the most significantly downregulated protein. The experiment was performed once on three independent biological replicates per group; *p*-values are calculated by two-tailed unpaired *t*-test. **D** Volcano plot showing changes in O-glycopeptide abundance from the same samples as in (**C**), enriched using jacalin-based lectin weak affinity chromatography (LWAC) and analyzed by TMT-MS. In red and blue are glycopeptides significantly decreased or increased in GOLPH3^−/−^ livers, respectively. Known GALNT2 substrates (e.g., Kng1, Apoc4, Lamp1) show reduced glycosylation in GOLPH3^−/−^ livers. The experiment was performed once on three independent biological replicates per group; p-values are calculated by two-tailed unpaired t-test. **E** Diagram of the mucin-type O-glycosylation pathway, illustrating key enzymes and intermediates. GALNTs initiate glycosylation by transferring GalNAc to Ser/Thr residues. **F** Schematic showing O-glycosylation sites in glycoproteins (e.g., Kng1, Apoc4, Lamp1) with decreased glycopeptide abundance in GOLPH3^−/−^ livers. Red arrowheads indicate a decrease; cyan equality symbols indicate no change. **G** Volcano plot depicting differential lipid species abundance between GOLPH3^+/+^ and GOLPH3^−/−^ livers (*n* = 10). Hexosylceramides (HexCer, blue) are elevated, while triglycerides (TAGs, red) are reduced in GOLPH3^−/−^ males. The experiment was performed once on ten independent biological replicates per group; p-values are calculated by two-tailed unpaired t-test. **H** Box-and-whisker plot (whiskers indicate min and max values) showing normalized abundance of major lipid classes. The experiment was performed once on ten independent biological replicates per group; p-values are calculated by two-tailed unpaired *t*-test. **I** Box-and-whisker plot (whiskers indicate min and max values) showing quantification of individual GlcCer and GalCer species via targeted LC-MS/MS across genotypes. The experiment was performed once on independent biological replicates; p-values are calculated by two-way ANOVA. GOLPH3^+/+^ *n* = 16; GOLPH3^+/-^ *n* = 22; GOLPH3^−/−^ *n* = 9. **J** Box-and-whisker plot (whiskers indicate min and max values) showing quantification of glycosphingolipid intermediates and products via LC-MS/MS. The experiment was performed once on independent biological replicates; p-values are calculated by two-way ANOVA. GOLPH3^+/+^ *n* = 16; GOLPH3^+/-^ *n* = 22; GOLPH3^−/−^ *n* = 9. **K** Schematic of the glycosphingolipid biosynthesis pathway. GOLPH3-dependent LCS converts GlcCer to LacCer, which serves as a precursor for downstream gangliosides and globosides. Accumulation of GlcCer in GOLPH3^−/−^ mice indicates reduced LCS function.

While GOLPH3 overexpression has been linked to solid tumor progression^21^, the physiological impact of GOLPH3 loss of function in vivo remains largely unknown. To address this, we investigated the systemic effects of GOLPH3 deficiency in mice (Fig. 1A). We first evaluated GOLPH3 expression at the mRNA and protein levels across multiple tissues in male and female mice at 15 and 41 weeks of age. GOLPH3 was ubiquitously expressed with minimal variation across age or sex (Fig. S1A, B), consistent with single-cell RNA-seq data from the Tabula Muris atlas^22^, which reports broad GOLPH3 expression in 20 mouse tissues (Fig. S1C). Notably, GOLPH3 has a paralog, GOLPH3L, with overlapping function but more restricted expression, primarily in highly glycosylating cells such as goblet cells in the large intestine (Fig. S1C).

We generated GOLPH3 loss of function (GOLPH3^−/−^) mice via CRISPR/Cas-mediated deletion of exons 2 and 3 (Fig. 1A, see methods). Western blotting on knockout livers confirmed loss of full length GOLPH3 protein (Fig. 1B).

To examine the consequences for glycosylation, we focused on the liver, a key site of glycoprotein production. Tandem mass tag (TMT)-based quantitative proteomics identified few significant changes among >2500 peptides in GOLPH3^−/−^ livers, with GOLPH3 itself being the most significantly downregulated protein (Fig. 1C). The residual detection of a GOLPH3 peptide (aa 28 to 44) in GOLPH3^−/−^ livers is likely linked to an unstable and truncated product due to our gene targeting strategy (see methods). However, when the same samples were analyzed using lectin weak affinity chromatography (LWAC) followed by TMT-MS to enrich for T/Tn antigens O-glycopeptides, a distinct subset was markedly reduced. These included known substrates of the GOLPH3 client GALNT2, such as APOC4 and LAMP1^23^.

To evaluate the possibility that these changes were due to indirect effects of GOLPH3 loss of function on the expression of the glycosylation machinery we performed bulk RNAseq (Fig. S1D). This experiment revealed 58 significantly downregulated genes (*p* < 0.05, FC <−0.5) and 9 significantly upregulated genes (*p* < 0.05, FC > 0.5). Inspection of a curated set of 238 glycogenes found no significantly altered glycogene suggesting that impaired O-glycosylation is primarily due to client posttranscriptional misregulation (Fig. 1D–F).

We next performed untargeted LC-MS lipidomics on GOLPH3^−/−^ livers. Most lipid classes were unchanged, but hexosylceramide (HexCer) levels were consistently elevated in both male and female knockouts (Fig. 1G–H). In contrast, triglyceride levels were selectively reduced in male GOLPH3^−/−^ livers (Fig. S1D, E).

Because standard LC-MS cannot differentiate isobaric species like glucosylceramide (GlcCer) and galactosylceramide (GalCer), we employed targeted LC-MS/MS. This analysis revealed a significant increase in GlcCer, but not GalCer, in GOLPH3^−/−^ livers compared to controls (Fig. 1I). Further sphingolipidomic profiling confirmed elevated GlcCer levels, while downstream metabolites of LCS activity remained unchanged (Fig. 1J).

These findings indicate reduced LCS activity in the absence of GOLPH3, resulting in a metabolic bottleneck and GlcCer accumulation (Fig. 1K). This phenotype parallels our earlier findings in HeLa cells, where GOLPH3 knockdown impaired LCS localization and stability^10^. Together, our results demonstrate that GOLPH3 orchestrates glycosylation and lipid metabolism in vivo, likely by maintaining the proper localization and function of its enzyme clients.

### GOLPH3 loss of function results in partially penetrant embryonic lethality and bone defects

**Fig. 2:**
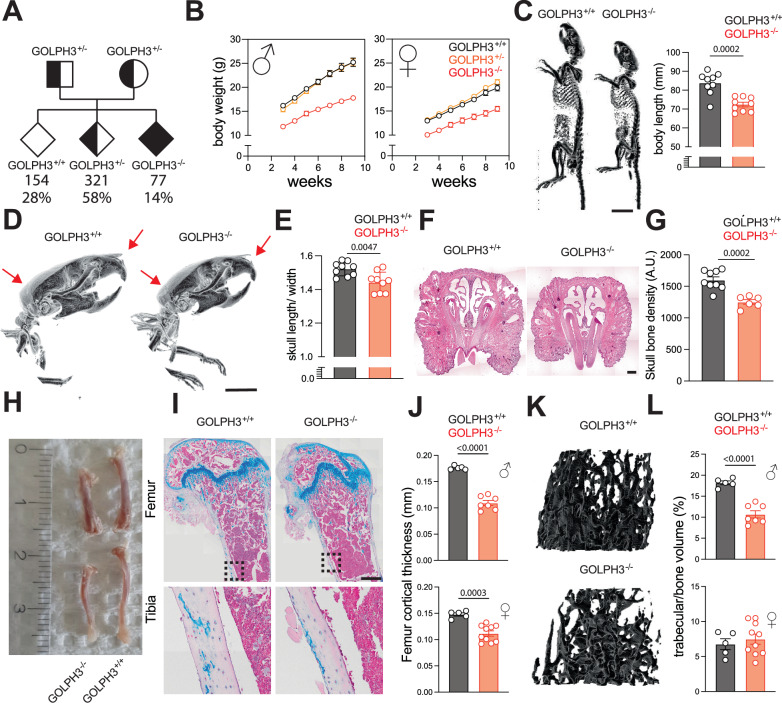
GOLPH3 loss of function leads to partially penetrant embryonic lethality and bone defects. **A** Pedigree chart showing genotype frequencies among offspring from GOLPH3^+/-^ × GOLPH3^+/-^ crosses. **B** Growth curves displaying body weight trajectories for male (left) and female (right) GOLPH3^+/+^, GOLPH3^+/-^, and GOLPH3^-/-^ mice between 3 and 9 weeks of age. Data are presented as mean ± SEM. The measurement were performed each week on the same cohort of animals. **C** Representative whole-body micro-computed tomography (µCT) scans of 9-week-old GOLPH3^+/+^ and GOLPH3^-/-^ mice (left). Quantification of body length is shown on the right. Data are mean ± SEM; The experiment was performed once on independent biological replicates; p-values are calculated by unpaired *t*-test. Bar = 10 mm; *n* = 9. **D** Representative µCT scans of the skull from 9-week-old male GOLPH3^+/+^ and GOLPH3^−/−^ mice. Red arrows highlight craniofacial anomalies, including reduced snout length and diminished cranial bulging in GOLPH3^−/−^ mice. Bar = 5 mm. **E** Quantification of skull length-to-width ratio in 9-week-old GOLPH3^+/+^ and GOLPH3^−/−^ mice. Data are mean ± SEM; the experiment was performed once on nine independent biological replicates per group; p-values are calculated by unpaired *t*-test. **F** Representative hematoxylin and eosin (H&E)-stained coronal sections of the nasal cavity in 9-week-old GOLPH3^+/+^ and GOLPH3^−/−^ mice. Bar = 1 mm. **G** Quantification of skull bone density in GOLPH3^+/+^ and GOLPH3^-/-^ mice assessed by µCT. Data are mean ± SEM; the experiment was performed once on nine independent biological replicates per group; p-values are calculated by unpaired *t*-test. **H** Representative photograph of femurs and tibias from 9-week-old GOLPH3^+/+^ and GOLPH3^−/−^ mice. **I** Representative Alcian Blue-stained decalcified femur sections from 9-week-old GOLPH3^+/+^ and GOLPH3^−/−^ mice. Insets show magnified views of cortical bone regions. Bar = 500 µm. **J** µCT-based quantification of femoral cortical thickness in male (top) and female (bottom) GOLPH3^+/+^ and GOLPH3^−/−^ mice. Data are mean ± SEM; the experiment was performed once on independent biological replicates; *p*-values are calculated by unpaired t-test. GOLPH3^+/+^ males *n* = 5; GOLPH3^−/−^ males *n* = 7; GOLPH3^+/+^ females *n* = 5; GOLPH3^−/−^ females *n* = 10. **K** Representative 3D reconstructions of femoral trabecular bone from 9-week-old male GOLPH3^+/+^ and GOLPH3^−/−^ mice. Bar = 200 µm **L** Quantification of femoral trabecular bone volume fraction (%) in male (top) and female (bottom) mice. Data are mean ± SEM; the experiment was performed once on independent biological replicates; *p*-values are calculated by unpaired *t*-test. GOLPH3^+/+^ males *n* = 5; GOLPH3^-/-^ males *n* = 7; GOLPH3^+/+^ females *n* = 5; GOLPH3^−/−^ females *n* = 10.

Strikingly, GOLPH3^+/−^ intercrosses yielded a significant deviation from Mendelian expectations. Among 552 offspring, only 77 (14%) were GOLPH3^−/−^, compared to 154 (28%) GOLPH3^+/+^ and 321 (58%) GOLPH3^+/−^ (χ², *p* < 0.0001), indicating substantial embryonic lethality in GOLPH3-null mice (Fig. 2A).

Surviving GOLPH3^−/−^ mice showed marked growth defects. Both males and females exhibited a 20–30% reduction in body weight between weeks 3 and 9 (Fig. 2B), driven by losses in both fat and lean mass as shown by EchoMRI (Fig. S2A). Body length was also significantly reduced (Fig. 2C). While fasting glucose levels were lower in GOLPH3^−/−^ mice, insulin and glucose tolerance remained unchanged across genotypes (Fig. S2B, C). Organ weights were generally reduced, but this effect was normalized when adjusted for total body weight, except in the brain, which showed a relatively increased mass in GOLPH3^−/−^ mice (Fig. S2D).

Craniofacial and skeletal anomalies further revealed a role for GOLPH3 in bone development. GOLPH3^−/−^ mice had shorter snouts, reduced skull doming (Fig. 2D, E), and diminished skull bone density on µCT, despite preserved nasal cavity structure (Fig. 2F, G). Femoral and tibial lengths were ~66% of controls in both sexes (Fig. 2H, and S2E), indicating global skeletal stunting. Cortical bone thickness was also reduced, particularly in males, by 30% in the femur and 15% in the tibia, whereas females showed milder effects (Fig. 2I, J; Fig. S2F).

Trabecular bone defects exhibited sex-specific differences: male GOLPH3^−/−^ mice had significantly less dense trabeculae compared to controls, a phenotype not observed in females (Fig. 2K, L; Fig. S2G). Together, these findings demonstrate that GOLPH3 loss impairs growth and bone architecture, with more severe consequences in males. Notably, some skeletal features resemble those of GALNT2 knockout mice^24^ and of humans bearing loss-of-function mutations in GALNT2^23^, linking GOLPH3-dependent Golgi trafficking to bone development via its glycosyltransferase clients.

### Regulated topology of GOLPH3 membrane association

The evidence above underscores the physiological importance of GOLPH3. This primed us to investigate its role as a Golgi adapter at the molecular level. Because client interaction depends on GOLPH3’s membrane association, we first examined its binding to the Golgi bilayer using coarse-grained (CG) molecular dynamics (MD) simulations.

**Fig. 3:**
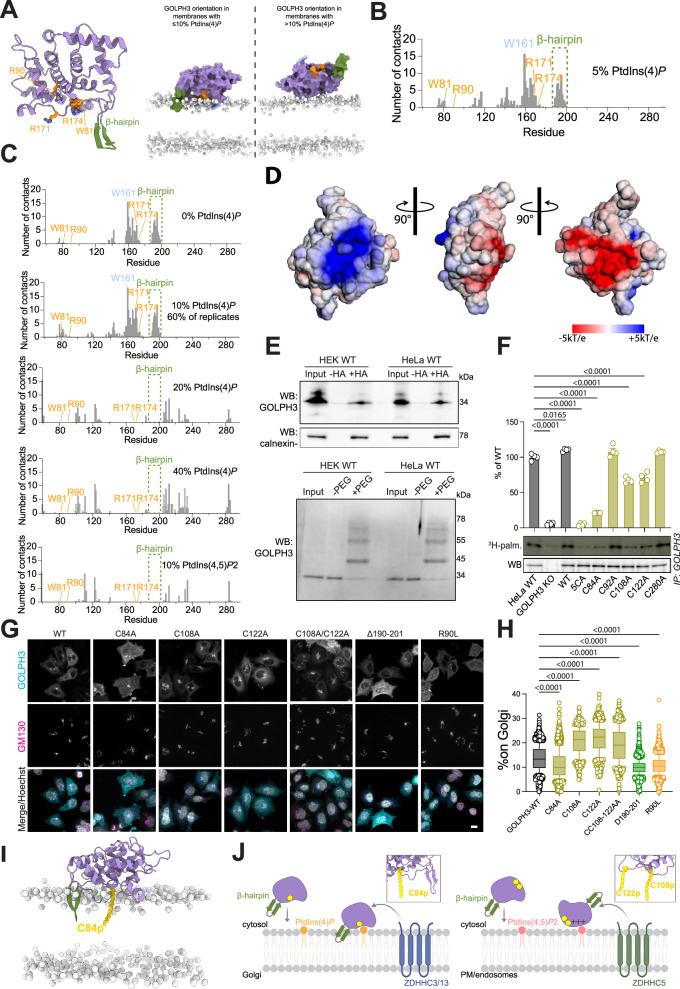
GOLPH3 membrane orientation. **A** Structure of GOLPH3 (left, from PDB 3KN1) and snapshots from coarse-grained MD simulations with membranes containing 5% (middle) or 10% (right) PtdIns(4)P. At 5%, GOLPH3 (purple surface) interacts via its β-hairpin (green), PtdIns(4)*P*-binding residues (W81, R90, R171, R174; orange), and Trp161 (blue). At 10% and higher, binding is mediated by a positively charged surface. Lipid head groups are shown as gray spheres; other lipids and water are omitted for clarity. **B** Average number of contacts between GOLPH3 residues and membranes with 5% PtdIns(4)P across MD trajectories. The experiment was performed on five independent replicates. **C** MD-based contact analysis of GOLPH3 with membranes containing 0%, 10% (detected in 60% of replicates), 20%, 40% PtdIns(4)*P*, or 10% PtdIns(4,5)*P*₂. The experiment was performed on five independent replicates per condition. **D** Electrostatic surface potential of GOLPH3 WT computed via the APBS website, by assigning charges to PDB 3KN1 with the PDB2PQR module using standard settings. **E** S-acylation of endogenous GOLPH3 in HEK and HeLa cells. Palmitoylated proteins were detected by hydroxylamine treatment (+HA), followed by SDS-PAGE and anti-GOLPH3 immunoblotting. Calnexin was used as a loading control (Top). Stoichiometry of GOLPH3 palmitoylation determined via PEG-5 labeling and anti-GOLPH3 western blotting (Bottom). Both experiments were performed on three independent biological replicates. **F** Palmitoylation analysis of GOLPH3 cysteine mutants. GOLPH3-KO HeLa cells were transfected with WT or mutant constructs, labeled with ³H-palmitic acid, and analyzed by autoradiography following immunoprecipitation. Quantification (mean ± SEM, *n* = 4) is relative to endogenous WT GOLPH3. The first lane refers to WT HeLa cells, the following lanes to GOLPH3-KO HeLa cells transfected with the indicated GOLPH3 mutants. The experiment was performed once on four independent replicates per condition; p-values are calculated by ordinary one-way ANOVA vs. HeLa WT. **G** Immunofluorescence of HeLa cells expressing WT or mutant GOLPH3, labeled with antibodies against GOLPH3, GM130 (Golgi), and Hoechst (nuclei). Scale bars = 10 μm (Top). Data are representative images from experiments performed on four independent biological replicates per condition. **H** Quantification of Golgi-to-cytosol GOLPH3 intensity ratio (Bottom). Whiskers show 2.5th–97.5th percentile; outliers as dots. *P*-values are calculated by one-way ANOVA vs. WT. Only transfected cells were analyzed (defined by GOLPH3 intensity > mean + 10 SD of non-transfected cells) *n* > 500. **I** MD model of S-acylated GOLPH3 (C84) interacting with membranes. **J** Proposed model of GOLPH3 membrane recruitment, integrating electrostatic interactions (via PtdIns(4)*P*-binding residues or a positively charged surface), β-hairpin insertion, and S-acylation at C84, C108, and C122.

We set up GOLPH3 (PDB: 3KN1) at ~60 Å from a membrane modeled with a lipid composition based on lipidomics of Golgi isolates, including 5% molar PtdIns(4)*P*^25^ (see Methods) (Fig. 3A). These simulations revealed rapid and stable membrane association, with GOLPH3 binding after 0.63 ± 0.26 µs on average (Fig. S3A).

GOLPH3 consistently approached the membrane via a specific surface. In this orientation, the hydrophobic β-hairpin (residues 190–201) and W161 inserted into the bilayer, while key PtdIns(4)*P*-binding residues (W81, R90, R171, R174)^12,13^ were positioned near the membrane (Fig. 3A, B). This orientation aligns with prior biochemical studies using purified GOLPH3^12^ and with poses returned by the protein-membrane interaction servers OPM^26,27^, DREAMM^28^, and MODA^29^ and with AlphaFold3 prediction of the complex between the protein and 50 lipid molecules^26,30^ (Fig. S3B, C). The different methods consistently identified identical membrane-interacting residues (80, 161, 194–197) with OPM predicting a binding free energy of −8.7 kcal·mol^−1^.

To assess how PtdIns(4)*P* influences membrane binding, we ran CG MD simulations with increasing concentrations of PtdIns(4)*P* (0–40 mol%). GOLPH3 associated with membranes even in the absence of PtdIns(4)*P* (binding in 5 of 6 MD replicas), maintaining the same binding surface as at physiological PtdIns(4)*P* levels (5 mol%) (Fig. 3C).

Remarkably, at higher PtdIns(4)*P* levels (10 mol%), GOLPH3 adopted an inverted orientation in ~40% of cases, engaging the membrane via a completely distinct, positively charged surface located on the opposing side of the protein (Fig. 3A, C-D). This flipped conformation became exclusive at 20 and 40 mol% PtdIns(4)*P*. Substituting PtdIns(4)*P* with the more negatively charged PtdIns(4,5)*P*₂ (10 mol%) also drove exclusive binding through the positively charged surface (Fig. 3C, D).

Other negatively charged lipids, phosphatidylserine and cardiolipin (20 mol%), did not replicate this effect (Fig. S3D), indicating that total negative charge alone is insufficient to promote this alternative membrane association mode. Instead, the specific charge distribution of the inositol headgroup appears critical.

In summary, CG MD simulations suggest that while PtdIns(4)*P* is not strictly required for GOLPH3’s initial membrane association, it controls the binding orientation in a concentration-dependent manner.

This orientation of GOLPH3 proposed by our simulations led us to investigate whether other mechanisms could lead to localization to the *trans*-Golgi. A particularly suited modification is S-acylation, the only reversible modification, which would be compatible with reversible, alternating membrane binding. The SwissPalm webserver^31^ predicts S-acylation at Cys122, a cysteine conserved in mouse GOLPH3 but absent in its paralog GOLPH3L and the yeast homolog Vps74p (Fig. S4A).

To test whether GOLPH3 can be S-acylated, we employed the Acyl-Resin-Assisted Capture (Acyl‑RAC) method^32^, which selectively captures S-acylated proteins by hydrolyzing thioester bonds and binding the resulting free thiols to thiopropyl beads for isolation followed by western blot detection. This approach in HEK293 and HeLa cells indicated that endogenous GOLPH3 is S-acylated at steady state (Fig. 3E). Since Acyl‑RAC does not resolve the proportion of S-acylated protein nor the number of acylated sites, we used Acyl-PEG, a related method where free thiols resulting from hydroxylamine treatment are labeled by PEG-maleimide, which causes gel-shifts proportional to the number of modified cysteines. PEGylation revealed at least three S-acylated cysteines, with 97% of GOLPH3 molecules being S-acylated on at least one cysteine (54% mono-acylated, 30% di-acylated, and 13% tri-acylated). This demonstrates that the vast majority of GOLPH3 molecules contain at least one acylated site under our experimental conditions (Fig. 3E, and Fig. S4B).

To confirm S-acylation and identify the sites, we generated five GOLPH3 single-point mutants (Cys-to-Ala), along with a quintuple mutant (GOLPH3-5CA) in which all cysteines were mutated. Using [³H]-palmitate metabolic labeling^33^ (see Methods), we found that wild-type GOLPH3 is robustly palmitoylated. Mutation of C84 markedly reduced labeling (~80% loss), while Cys108 and Cys122 mutations had a modest effect (Fig. 3F). GOLPH3-5CA showed no detectable palmitoylation, indicating that Cys84, Cys108, and Cys122 are bona fide palmitoylation sites.

Structural analysis revealed that concurrent acylation at Cys84, Cys108, and Cys122 is incompatible with a single membrane orientation (Fig. S4C). Cys84 aligns with the membrane-binding surface that accommodates β-hairpin insertion and PtdIns(4)*P* binding (Fig. S4C). In contrast, Cys108 and Cys122 are positioned on the opposite side of the protein, supporting an inverted orientation involving GOLPH3’s positively charged surface (Fig. 3A, D and Fig. S4C).

To identify the responsible S-acyltransferases, we used six siRNA pools covering the 23 human ZDHHC enzymes in HeLa cells and assessed GOLPH3 acylation by Acyl‑RAC (Fig. S4D–F). Mixes 1 and 3, which respectively contained ZDHHCs 1, 3, 7, 13, and 17 and ZDHHCs 5, 8, 9, and 20, led to a major decrease in recovery by Acyl‑RAC (Fig. S4F). Silencing of individual enzymes present in these pools showed that knockdown of ZDHHC5, ZDHHC13, and to a lesser extent ZDHHC3, reduced GOLPH3 acylation. Further dissection using the S-acylation defective mutants revealed that ZDHHC13 targets Cys84, while ZDHHC5 modifies Cys108 and Cys122 (Fig. S4G, H).

This could be further confirmed using click-PEG metabolic labeling^34^. After 4 h of labeling, a shifted band corresponding to acylated GOLPH3 was observed; this band was lost upon silencing of ZDHHC5 or ZDHHC3/13, confirming the requirement for these acyltransferases and suggesting interdependence among S-acylation sites. Inhibition of APT2 with Palmostatin B^35^ or ML349^36^ increased the click-PEG signal, indicating APT2-mediated depalmitoylation. Collectively, these data establish that the GOLPH3 population undergoes dynamic, site-specific acylation–deacylation cycles regulated by ZDHHC enzymes and APT2 (Fig. S4I).

We next asked whether acylation affects GOLPH3 localization. Quantitative immunofluorescence showed that mutation of C84 reduced Golgi association, and resulted in GOLPH3 relocation to the cell surface (Fig. 3G). Conversely, mutation of C108 and/or C122 increased Golgi localization, suggesting that acylation at these sites may antagonize Golgi targeting (Fig. 3G).

Both PtdIns(4)*P* binding and S-acylation at Cys84 appear to contribute to Golgi localization (Fig. 3G). To understand the interplay between these regulatory inputs, we perturbed them both and monitored GOLPH3 localization (Fig. S5A). Cells expressing GOLPH3 WT or S-acylation mutants were treated with the PI4KIIIβ inhibitor PIK93 to deplete TGN PtdIns(4)*P*^37^. Compared with the canonical TGN PtdIns(4)*P* binder CERT, WT GOLPH3 was more resistant to PtdIns(4)*P* depletion, indicating that PtdIns(4)*P* is important for initial recruitment but not strictly required for retention. Mutation of the PtdIns(4)*P*-binding site (R90L) largely abolished Golgi localization; the small residual Golgi fraction of R90L was insensitive to PIK93, consistent with loss of the lipid-dependent recruitment step. Preventing S-acylation at Cys84 (C84A) decreased Golgi localization and rendered GOLPH3 sensitive to PIK93, showing that Cys84 acylation contributes to membrane retention when PtdIns(4)*P* is limiting. By contrast, mutation of Cys108 and Cys122 produced a strongly Golgi-associated, PIK93-insensitive form. Notably, ZDHHC13 localizes to the Golgi, while ZDHHC5 is distributed throughout the secretory pathway and the plasma membrane^38,39^. Supporting this spatial logic, super-resolution microscopy data (Fig. S5B) show that ZDHHC13 is significantly enriched at the *trans*-side of the Golgi. This places the acyltransferase precisely where it is needed to lock GOLPH3 into its PtdIns(4)*P*-bound state in a form of coincidence detection mechanism.

Altogether, these findings support a model in which GOLPH3 binds distinct intracellular membranes via two opposing surfaces. At the Golgi, where ZDHHC13 is localized, GOLPH3 inserts its β-hairpin, interacts with PtdIns(4)*P*, and undergoes acylation at Cys84 (Fig. 3I, J). In contrast, at the plasma membrane, it may interact with PtdIns(4,5)*P*₂ through its positively charged surface, encounter ZDHHC5, resulting in modification at Cys108 and Cys122, which favors this alternative membrane-binding mode. This model suggests that S-acylation dynamically regulates both the subcellular localization and membrane orientation of GOLPH3.

### Identification of the GOLPH3-client enzyme interacting surface

**Fig. 4:**
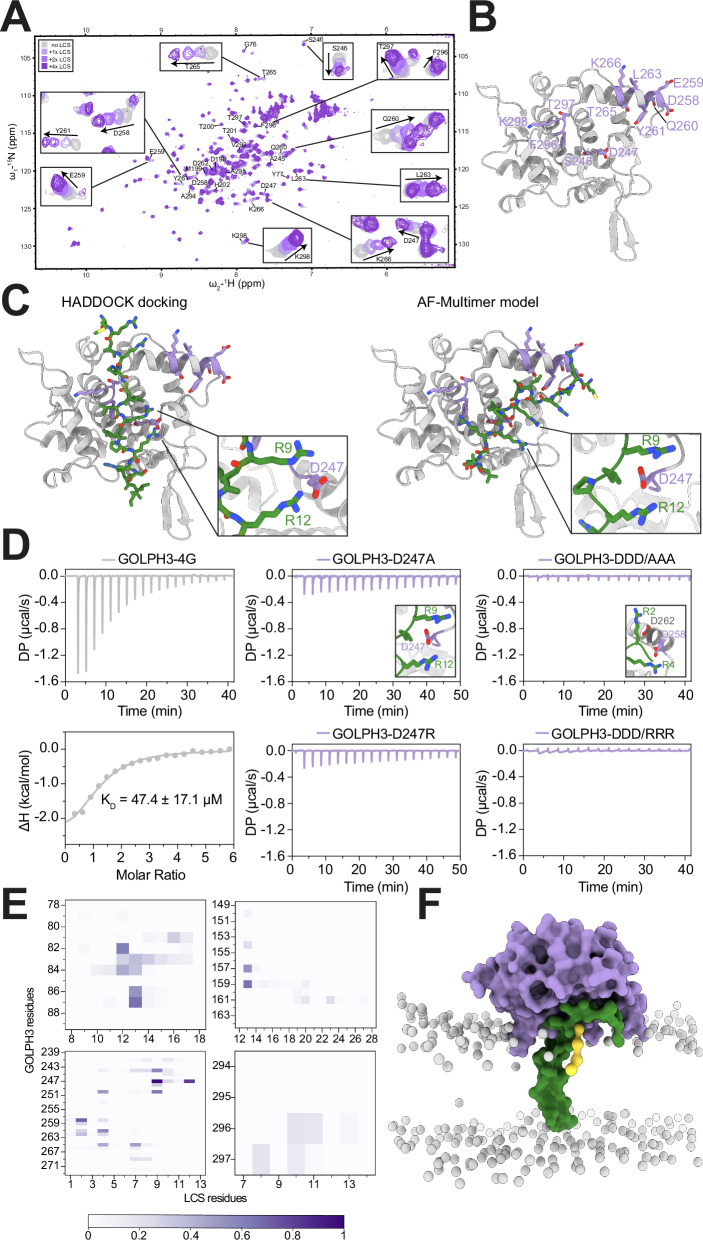
LCS binding site on GOLPH3 as mapped by NMR. **A** Overlay of 2D ^1^H–^15^N HSQC spectra of ^15^N-labeled GOLPH3 in its apo form (gray) and with increasing molar ratios of LCS peptide (1:1 to 4:1, gradient gray to violet). Assigned residues are labeled; boxes highlight regions with notable chemical shift changes. The experiment was once. **B** NMR-mapped LCS binding site on the negatively charged surface of GOLPH3, involving residues S246, D247, D258, E259, Q260, Y261, L263, T265, K266, F296, T297, and K298. The partial backbone assignments for human GOLPH3 are available at the BMRB under the ID 53516 and in Table S1 together with the chemical shift perturbation obtained from the NMR data. **C** Structural models of the GOLPH3–LCS complex from HADDOCK (guided by NMR data) and AlphaFold-Multimer (unguided). Insets show electrostatic interactions. LCS is in green; GOLPH3 residues with NMR shifts are in purple, others in gray. Coordinates for the two structural models shown in this panel are available for download at zenodo.org (doi.org/10.5281/zenodo.18430252). The AlphaFold model is also available at the ModelArchive with ID ma-c5v3o, and the HADDOCK model at the PDB-IHM with ID 9AAE. **D** ITC binding curves for LCS WT with GOLPH3 D247R (left) and D247R/D258R/D262R (middle), and LCS R9A/R12A mutant with GOLPH3 WT (right). Representative of 2–3 independent replicates. **E** Normalized contact heatmaps showing residue-level interactions between GOLPH3 and LCS during CG MD simulations. **F** CG MD snapshot of S-acylated GOLPH3 (at C84) bound to the cytosolic tail and transmembrane domain of LCS (green) at the membrane.

Given the aforementioned effects of S-acylation on GOLPH3 orientation, and presumably its function, we next investigated which region of the GOLPH3 protein interacts with the cytosolic tails of its client enzymes^10^. To this end, we employed Nuclear Magnetic Resonance (NMR) spectroscopy, selecting the cytosolic tail of LCS, which exhibits micromolar-range binding affinity for GOLPH3^10^ (Fig. 4).

Due to the low solubility of recombinant wild-type (WT) GOLPH3, we engineered a mutant (F194 G/L195G/L196G/F197G; hereafter GOLPH3-4G) that reduces hydrophobicity at the β-hairpin tip, improving solubility. GOLPH3-4G exhibited slightly higher thermal stability (Fig. S6A), remained monomeric by SEC-MALS (Fig. S6B), retained comparable folding and LCS-binding affinity as WT as judged by largely overlapping ^1^H, ^15^N HSQC spectra and isothermal titration calorimetry (ITC) (K_D_ = 65.3 ± 8.9 µM for WT and K_D_ = 47.4 ± 17.1 µM, for GOLPH3-4G; Fig. S6C, D).

Titrating the LCS tail peptide into ^15^N-labeled GOLPH3-4G revealed widespread chemical shift perturbations (Fig. 4A, and Table S1), indicating extensive interaction. The observed fast exchange regime and absence of saturation are consistent with a high micromolar dissociation constant, in agreement with ITC measurements (Fig. S6D).

To map the binding interface, we assigned perturbed residues using triple-resonance spectra of ^15^N,^13^C-labeled GOLPH3-4G under titration conditions. Despite limited spectral quality, we confidently assigned seven residues that shifted strongly upon peptide binding (S246, D247, T265, K266, F296, T297, K298), and 19 that showed small or no shift upon peptide titration (Table S1, see Methods). Additional assignments via single-residue mutants led us to assign residues in the 258–263 stretch, five of which (D258, E259, Q260, Y261, L263) shifted upon titration (Fig. S7A–C).

These data identify a broad, negatively charged interaction surface on GOLPH3, comprising at least S246, D247, D258, E259, Q260, Y261, L263, T265, K266, F296, T297, and K298 (Fig. 4B). Given the highly basic sequence of the LCS peptide (MRARRGLLRLPRSLLA), this supports a weak, low-specificity electrostatic interaction in solution. However, the effective affinity of the GOLPH3–LCS complex may be higher under physiological conditions, due to the two-dimensional confinement imposed by the membrane surface.

To further explore the interface, we performed computational docking of the LCS peptide to GOLPH3 using HADDOCK^40,41^, guided by NMR-identified “active residues”. Docking revealed dominant electrostatic interactions: LCS R2 and R4 with GOLPH3 D273, R9 and R12 with D247, and R13 with E159 (Fig. 4C). Hydrophobic contacts also contributed, with LCS leucines engaging pockets on GOLPH3: Y241, H244, L272, and V293 with L7; F296 with L8; and W81, S86, and L16 with LCS’s C-terminal leucines. Notably, W81 is a PtdIns(4)*P*-binding residue and may be unavailable in a physiological context.

Because the chemical shift perturbations span a larger area than can be explained by a single docking pose, we used AlphaFold-Multimer^42^ and AlphaFold 3^30^ for blind prediction of the LCS-GOLPH3 complex. The resulting models recapitulated key electrostatic interactions observed in HADDOCK and consistent with the NMR mapping: R2 and R4 with D258 and D262; R9 and R12 with D247; R13 with E159. Hydrophobic interactions were also consistent, involving H244, L249, F253, V268, L272, and Y261 engaging L7–L8 of LCS (Fig. 4C). AlphaFold 3 in particular, executed with 1000 runs, yields highly accurate metrics for the GOLPH3-LCS interface: pTM as high as 0.89, ipTM at 0.53, and low local RMSDs in the PAE plots at the GOLPH3-LCS interface (Fig. S8A, B). Despite not being guided by experimental data, the AlphaFold-Multimer model was very consistent closely aligned with the NMR-mapped residues, reinforcing its validity.

A common assumption of these co-folding models is that the LCS peptide is unstructured. When LCS cytosolic tail was assessed by CD experimentally no evidence of stable secondary structure was found nor in the full-length tail nor in truncation variants. Incubating the peptide with increasing concentrations of TFE (2,2,2-trifluoroethanol), which stabilizes α-helices by strengthening intramolecular H-bonds did not induce any significant folding suggesting that indeed LCS cytosolic tail is unstructured (Fig. S7C, D).

Among the predicted contacts, the interaction between GOLPH3 D247 and LCS R9/R12 emerged as a central binding hub. To validate this, we tested WT GOLPH3 with LCS mutants (R9A, R12A, R9A/R12A) and GOLPH3 mutants (D247A, D247R) using ITC. All mutations reduced binding, with combined mutations of D247, D258, and D262 abolishing interaction (Fig. 4D, and Fig. S8A, B). Importantly, all mutants retained proper folding and thermal stability (Fig. S8C, D and Table S2), confirming that binding loss was not due to protein misfolding.

We next modeled the GOLPH3–client complex in the membrane context using CG MD simulations. The system included the cytosolic tail and transmembrane domain of LCS bound to GOLPH3 on a Golgi-like lipid bilayer, using the AlphaFold-Multimer pose as the starting configuration (Fig. 4C). Simulations showed stable GOLPH3–LCS association, with the LCS tail exploring the GOLPH3 surface while its transmembrane helix remained embedded (Fig. 4E).

GOLPH3 maintained a membrane orientation consistent with prior membrane-association modeling and stabilized by Cys84 S-acylation (Fig. 3J). When C84 S-acylation was modeled explicitly, GOLPH3 embedded into the membrane without altering its binding to LCS (Fig. 4F, Fig. S8B).

Altogether, the combination of NMR, MD simulations, AlphaFold predictions, and HADDOCK modeling indicates that GOLPH3 binds its target substrate, LCS, through a well-defined groove lined with negatively charged residues, fully consistent with the membrane-associated conformation in which Cys84 is S-acylated.

### GOLPH3 regulation drives GOLPH3-clients retention at the Golgi

Once bound to GOLPH3, Golgi-resident proteins are packaged into COPI-coated vesicles, enabling their retrograde transport through maturing Golgi *cisternae*^10^. We thus investigated how perturbing the various GOLPH3 membrane binding modes described in this study would affect the trafficking of GOLPH3 clients. We made use of a previously established reporter assay based on a chimeric construct^10^, which consists of the transmembrane domain of the type II plasma membrane enzyme sucrose isomaltase (SI), harboring GFP at its C-terminus and the cytosolic tail of LCS at the N-terminus. Under normal conditions, the chimera (LCS–SI–GFP) localizes to the Golgi in a GOLPH3-dependent manner^10^. To assess the extent of reporter mislocalization to the plasma membrane, we used an anti-GFP antibody on non-permeabilized cells, which selectively detects GFP exposed at the cell surface. This allowed us to quantify the fraction of the reporter that escapes Golgi retention, based on the ratio of surface-exposed to total cellular GFP.

**Fig. 5:**
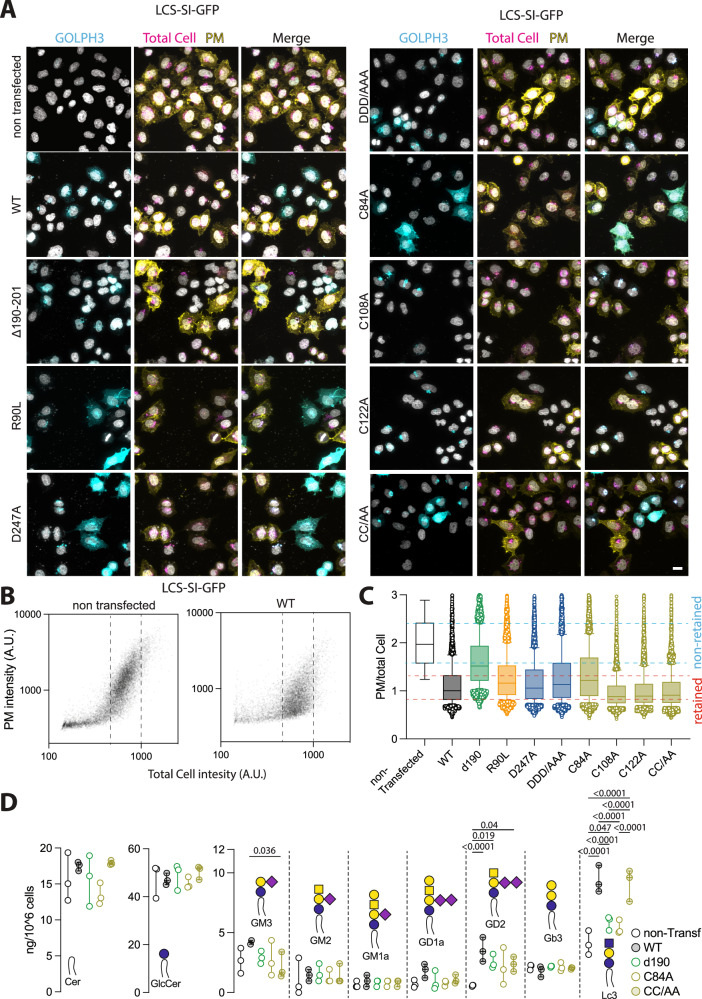
Regulated GOLPH3 interaction with Golgi membranes and clients drives GOLPH3-clients retention at the Golgi. **A** Quantitative immunofluorescence of LCS-SI-GFP Golgi retention in GOLPH3-KO HeLa cells transfected with various GOLPH3 variants. Scale bar: 10 µm. Staining: anti-GOLPH3 (cyan), anti-GFP before permeabilization (yellow), LCS-SI-GFP (magenta). Data are representative images from experiments performed on four independent biological replicates per condition. **B** Scatterplot showing total LCS-SI-GFP fluorescence (Total Cell intensity) and surface-exposed signal (PM intensity). Cells were classified as transfected or non-transfected based on GOLPH3 signal. Dashed lines indicate the intensity range used for downstream analysis. **C** Quantification of LCS-SI-GFP Golgi retention in cells expressing different GOLPH3 mutants, as in (**B**); Whiskers show 2.5th–97.5th percentile; outliers as dots; *n* > 1000. **D** Quantification of glycosphingolipid intermediates and products via LC-MS/MS. In GOLPH3-KO HeLa cells expressing different GOLPH3 mutants; the experiment was performed once on three independent biological replicates per condition; *p*-values are calculated by two-way ANOVA.

Mutants defective in either PtdIns(4)*P* binding (GOLPH3-R90L), membrane association (GOLPH3-Δ190–201), LCS tail-binding (GOLPH3-D247A and the triple mutant GOLPH3-D247A/D258A/D262A, hereafter referred to as GOLPH3-DDD/AAA), or S-acylation (GOLPH3-C84A, GOLPH3-C108A, GOLPH3-C122A, and the double mutant GOLPH3-C108A/C122A) were transfected into GOLPH3-knockout cells. Mutants that impaired GOLPH3 recruitment to the Golgi, particularly the β-hairpin deletion mutant GOLPH3-Δ190–201, showed markedly reduced retention of the LCS–SI–GFP chimera compared to GOLPH3-WT. Mutations that disrupted LCS binding also diminished retention, although incompletely. The residual activity suggests that, within the two-dimensional context of the membrane, the broad interaction surface between GOLPH3 and its clients is sufficient to maintain partial incorporation into COPI vesicles even when binding is weakened (Fig. 5A, B).

Interestingly, the effects of S-acylation mutants on LCS retention were divergent. GOLPH3-C84A, which exhibits defective Golgi localization, led to a reduction in retention activity. In contrast, GOLPH3-C108A, GOLPH3-C122A, and the double mutant GOLPH3-C108A/C122A fully retained the reporter at the Golgi, indicating that these modifications do not impair, and may even promote client retention (Fig. 5A–C).

To assess the functional consequences of GOLPH3 regulation, we performed targeted LC-MS/MS analysis of GSLs in GOLPH3-KO HeLa expressing GOLPH3-WT, the severely impaired GOLPH3-Δ190–201 mutant, GOLPH3-C84A, or GOLPH3-C108A/C122A. We found that the C84A mutant is less effective than wild-type GOLPH3 at promoting lipid glycosylation, whereas the C108A/C122A mutant displays active behavior (Fig. 5D).

While the physiological relevance of GOLPH3 S-acylation in regulating glycosylation and associated biological processes will require further in vivo investigations, our findings converge on a mechanistic model. Effective binding of GOLPH3 to its client proteins requires membrane association and specific orientation. In this membrane-bound state, the interaction with LCS spans an extended surface of GOLPH3, such that mutations disrupting binding, while sufficient to abolish interaction in biophysical assays, still allow partial functionality in cells. Moreover, S-acylation regulates two functionally distinct states of GOLPH3: acylation at C84 promotes stable Golgi membrane binding and client retention, whereas acylation at C108 and C122 counteracts Golgi localization and opposes LCS retention.

## Discussion

Glycosylation is a non-templated polymerization process^3^ in which the final glycan structures are largely determined by the sequence of glycosyltransferase encounters with cargoes. Given that the cellular glycome varies across cell states, types, and tissues, a central question in the field is how glycosylation programs are coordinated. Specifically, to what extent is transcriptional control of glycogenes sufficient, and do post-transcriptional mechanisms play an equally critical role? This is particularly relevant in light of our recent findings showing that glycolipids actively shape cell transcriptomes^43,44^.

The Golgi apparatus plays a central role in glycosylation, organizing the spatial and temporal sequence of enzymatic modifications^1^. This organization depends on the active retention of glycosyltransferases via retrograde COPI-coated vesicles. GOLPH3 is a key COPI adapter that anchors a specific subset of Golgi enzymes by binding their cytosolic tails^45^. Modulating GOLPH3 activity, therefore, presents a potential mechanism to selectively reprogram the glycome. This raises critical questions: Are COPI adapters like GOLPH3 themselves subject to regulation, and what are the consequences of their dysfunction?

### In vivo loss of GOLPH3 alters the glycome and causes growth and skeletal phenotypes

In this study, we found that systemic GOLPH3 loss of function in mice is associated with a global change in the protein and lipid glycosylation machinery albeit the specific glyco-enzymes driving these changes are yet to be established. In the liver, GOLPH3 deficiency causes a reduction of T/Tn–bearing O-glycopeptides and accumulation of GlcCer, changes that do not appear to be explainable by transcriptional reprogramming of glycogenes. While broader glycoform profiling and dedicated studies are needed to assign which exact enzymatic steps are directly affected by GOLPH3 loss of function, these data support a model in which GOLPH3 loss primarily impacts glycosylation post‑transcriptionally, by compromising Golgi retention and stability of multiple enzyme clients.

This interpretation is consonant with prior cellular studies from our group and others showing that GOLPH3 interacts with and stabilizes a broad spectrum of Golgi residents^10,11^. In mammalian cells, GOLPH3 and its paralog GOLPH3L bind at least ~20 glycosyltransferases and other Golgi proteins spanning multiple pathways^10,11^. For many of these clients, GOLPH3 depletion leads to their mislocalization to endolysosomal compartments and enhanced degradation^10,11^. The liver glycoproteomic and lipidomic phenotypes observed here, therefore, likely reflect the cumulative consequence of losing multiple enzyme clients across several pathways rather than a single enzymatic deficit.

GOLPH3 deficiency causes partially penetrant embryonic lethality and, in surviving mice, severe growth retardation with disproportionate shortening of long bones, reduced cortical and trabecular bone mass (more severe in males), and craniofacial anomalies. Human and mouse studies have established that mutations in individual glycogenes, including PMM2 (PMM2‑CDG), EXT1/EXT2 (multiple hereditary exostoses), GALNT3, GALNT2 and others, produce short stature, bone fragility, exostoses, or craniofacial dysmorphisms, underscoring the sensitivity of endochondral ossification and bone remodeling to glycosylation defects^46,47^. In particular, loss‑of‑function GALNT2 variants cause a congenital disorder of O‑linked glycosylation with distinctive skeletal anomalies^23^, and EXT1 is a central enzyme in heparan sulfate proteoglycan biosynthesis, which is critical for growth plate signaling^48^. Crucially, GALNT2 and EXT1 are both GOLPH3 clients^11^. Moreover, the Human Genetic Evidence Calculator (HuGE) identifies a very strong association between GOLPH3 and height variation in the human population through rare variant associations^49^, suggesting potential human correlates of the phenotypes observed in our study. Finally, our cellular studies demonstrate that GOLPH3 S-acylation is relevant for regulating glycosylation. Testing the role of GOLPH3 S-acylation on bone physiology directly in primary cells or in rescue experiments in knockout mice would further strengthen its relevance.

### Dual regulation of GOLPH3 by PtdIns(4)*P* and S‑acylation: a coincidence detector for the *trans*‑Golgi

Our structural, MD, and cell biological data together support a model in which GOLPH3 is regulated by two coupled inputs (i.e., phosphoinositide composition and reversible site‑specific S‑acylation) that together act as a coincidence detector for the *trans*‑Golgi.

Coarse‑grained MD simulations using Golgi‑like membranes reveal that GOLPH3 has an intrinsic tendency to approach membranes via the surface harboring its β‑hairpin and the canonical PtdIns(4)*P*‑binding site (W81, R90, R171, R174). At physiological PtdIns(4)*P* levels (low mol%), this orientation predominates and positions a broad, negatively charged groove facing the membrane. This is the same surface that recognizes the basic cytosolic tails of client glycosyltransferases such as LCS. At higher surface densities of PtdIns(4)*P*, or in the presence of PtdIns(4,5)P₂, GOLPH3 can instead adopt an inverted topology, engaging membranes through a positively charged patch on the opposite side of the protein.

We then found that GOLPH3 undergoes S‑acylation at three cysteines (Cys84, Cys108, Cys122), catalyzed in a site‑specific manner by distinct ZDHHC palmitoyltransferases: ZDHHC13 (and ZDHHC3) target Cys84, whereas ZDHHC5 modifies Cys108/Cys122. More than 95% of GOLPH3 molecules are acylated on at least one cysteine at steady state through active cycles of acylation and deacylation. Thus, GOLPH3 is not sparsely or irreversibly modified; rather, its entire population is dynamically palmitoylated at specific sites.

The functional consequences of these events are asymmetric. Cys84 lies on the β‑hairpin/PtdIns(4)*P*‑binding face and, when acylated, stabilizes the orientation in which this surface embeds into Golgi membranes and presents the client‑binding groove at the membrane interface. Consistent with this, Cys84A mutants show reduced Golgi localization, heightened sensitivity to PI4KIIIβ inhibition (PIK93), and diminished ability to retain the LCS‑SI‑GFP reporter and to restore GSL biosynthesis in GOLPH3-KO cells. By contrast, Cys108 and Cys122 reside on the opposite side of the fold and are associated with the alternative, positively charged membrane‑binding face. Their acylation correlates with reduced Golgi association and poorer retention activity, while C108A/C122A mutants are more strongly Golgi‑localized, relatively insensitive to PIK93, and fully competent for LCS‑SI‑GFP retention and GSL rescue.

Together with the distinct localizations of ZDHHC13 (enriched at the *trans*‑Golgi) and ZDHHC5 (broadly distributed), these data support a model in which: (i) initial Golgi recruitment of GOLPH3 is driven by β‑hairpin insertion and PtdIns(4)*P* recognition; (ii) at the *trans*‑Golgi, ZDHHC13/3 install Cys84 acylation, locking GOLPH3 into the PtdIns(4)*P*‑bound, client‑competent topology; and (iii) when GOLPH3 cycles to other membranes (e.g., plasma membrane), encounter with ZDHHC5 promotes Cys108/Cys122 acylation and engagement of the opposite face with PtdIns(4,5)P₂‑rich membranes, positioning the client‑binding surface away from the membrane and functionally disengaging GOLPH3 from Golgi enzyme retention.

In this model, PtdIns(4)*P* binding and Cys84 S‑acylation operate consecutively as a coincidence detector: both are needed to establish and maintain the active, Golgi‑resident state. This is supported experimentally by the differential response of WT, R90L (PtdIns(4)*P*‑binding deficient), C84A and C108A/C122A variants to PIK93. WT GOLPH3, once Golgi‑bound, is relatively resistant to acute PtdIns(4)*P* depletion, implying that C84 acylation compensates when PtdIns(4)*P* is limiting. R90L fails to populate the Golgi properly and is largely insensitive to PIK93, as expected for a mutant that no longer depends on PtdIns(4)*P*. C84A becomes more dependent on PtdIns(4)*P* for Golgi association, whereas C108A/C122A shows strong Golgi residency even when PtdIns(4)*P* is reduced, consistent with a model where preventing “inactivating” acylation biases GOLPH3 toward the client‑competent orientation.

### Client recognition and sorting

Using NMR chemical shift perturbation mapping, we identify a negatively charged and partially hydrophobic groove on GOLPH3 as the client‑binding surface. Basic residues in the LCS tail (notably R2, R4, R9, R12, R13) interact with acidic residues in GOLPH3 (D247, D258, D262, E159 and neighbors), and leucines within the LCS tail pack into shallow hydrophobic pockets. Mutations in central contact residues on either side (R9A/R12A in LCS; D247R and D247R/D258R/D262R in GOLPH3) weaken or abrogate binding in solution ITC and NMR assays, and reduce retention of the LCS‑SI‑GFP reporter in cells. In the course of this study, the same GOLPH3 groove has been identified by mutational analysis as responsible for the binding and Golgi retention of other GOLPH3 clients, substantiating our results^50^.

Importantly, this negatively charged groove is presented to the cytosol only when GOLPH3 is in the β‑hairpin/PtdIns(4)*P*/Cys84‑acylated orientation. This provides a post‑transcriptional mechanism to remodel the glycome: rather than changing glycogene expression, cells can alter where and for how long specific glycosyltransferases and lipid enzymes reside in the Golgi stack by modulating PI4KIIIβ activity, ZDHHC13/3/5 expression or localization, or APT2 activity. The consequences are expected to propagate through secreted and membrane glycoproteins, glycolipids, and proteoglycans, influencing cell signaling, adhesion, and matrix interactions.

This regulatory logic is relevant in development and in disease. In cancer, GOLPH3 is frequently amplified or overexpressed and has been linked to mTOR activation, altered Golgi morphology and increased GSL synthesis^51^. Our findings suggest that GOLPH3 hyperactivation could also stabilize subsets of glycosyltransferases and lipid enzymes at the Golgi, driving pro‑oncogenic glycome changes that enhance receptor signaling and immune evasion^52^. Conversely, dysregulation of ZDHHC13/5 or APT2 could perturb the balance between active and inactive GOLPH3 orientations, leading to aberrant enzyme retention and glycosylation patterns even without changes in GOLPH3 abundance.

Altogether, in this study, we identify GOLPH3 as a key node linking Golgi lipid composition to enzyme retention and glycosylation capacity, and show that its regulation by PtdIns(4)*P* and S‑acylation constitutes a previously unrecognized layer of control over the mammalian glycome and developmental programs.

## Methods

### Mice

The generation of GOLPH3^−/−^ mice was done by CRISPR/Cas-mediated genome engineering, generated in collaboration with Cyagen Biosciences, Santa Clara, CA, USA. Exons 2 and 3 were selected as the target site, and Cas9 and gRNA were co-injected into fertilized eggs of C57BL6/J mice. The pups were genotyped by PCR followed by sequencing analysis. The heterozygous progeny (GOLPH3^+/-^) were crossed and used to generate the experimental model used in this study, which are the full systemic GOLPH3 knockout mice (GOLPH3^−/−^).

Notably, exons 2 and 3 encompass residues 74–158. While the vast majority of mouse GOLPH3 transcripts include these exons, our excision strategy does not prevent the expression of isoforms that skip them. Based on our structural and cellular studies, deletion of residues 74–158 is expected to produce a largely non-functional protein. Furthermore, our proteomics and Western blot analyses indicate the absence of full-length GOLPH3 in liver tissue, along with a > 75% reduction in the abundance of GOLPH3 tryptic peptides in GOLPH3^−/−^ mice.

All cohorts were inbred at the EPFL breeding facility, and were maintained in a temperature-controlled environment with a 12-h light/12-h dark cycle and free access to standard chow diet and water according to the Swiss Animal Protection Ordinance (OPAn). Male and female animals were used in this study. Body composition (fat and lean mass) was measured using EchoMRI technology with the EchoMRI™ qNMR system (Echo Medical Systems, Houston, TX, USA), as well as whole skeleton morphology and bone mineral density (BMD) by micro-CT using Quantum Fx CT at the Center of PhenoGenomics (CPG) of the EPFL. Unless stated otherwise, animals were sacrificed at room temperature, by cervical dislocation, and all tissues were collected, either snap frozen, fixed for histological analysis. All animal care and treatment procedures were performed in accordance with the Swiss guidelines and were approved by the Canton of Vaud SCAV (authorization VD 3730.c).

### Liver tissue differential O-glycoproteomics

Approximately 30–50 mg of frozen, pulverized liver tissue (Golph3 WT or KO) was homogenized in 500 µL buffer. Samples were heated at 95 °C for 5 min, sonicated (5 × 5 s), and centrifuged at 21,000 × *g* for 20 min. Proteins were reduced with dithiothreitol and alkylated with iodoacetamide (Sigma), then digested overnight at 37 °C with trypsin (Roche) at a 1:40 ratio. Digests were acidified with trifluoroacetic acid (TFA) for 1 h at 37 °C to inactivate trypsin and quench RapiGest, and desalted using Sep-Pak C18 1cc columns (Waters).

Peptides were quantified using a colorimetric kit (Thermo Fisher), and 200 µg from each sample was labeled with 1.6 mg TMT sixplex (Thermo Fisher). TMT-labeling of peptide samples was prepared as previously described^53^. Labeled samples were pooled in equal amounts and treated with 0.2 U/mL neuraminidase from Clostridium perfringens (Sigma) to remove sialic acids. Samples were diluted in 2 mL Tris-HCl (175 mM, pH7.4), and O-glycopeptides (T and Tn antigens) were enriched via lectin weak affinity chromatography (LWAC) using jacalin-agarose beads (Vector Labs), as described in ref. ^54^. Bound glycopeptides were eluted with 3 × 1.0 M D-galactose and desalted using in-house StageTips (C18/C8, Empore 3 M). A 50 µg aliquot of the LWAC flow-through was fractionated for proteomic analysis. Both glycoproteome and proteome samples were analyzed by LC-MS/MS.

### Mass spectrometry-based proteomics

The LC-MS/MS analysis was performed by using an EASY-nLC 1000 UHPLC (Thermo Scientific) interfaced via a PicoView nanoSpray ion source (New Objectives) to an Orbitrap Fusion mass spectrometer (Thermo Scientific). Nano-LC was operated in a single analytical column setup using PicoFrit Emitters (New Objectives, 75-μm inner diameter) packed in-house with Reprosil-Pure-AQ C18 phase (Dr. Maisch, 1.9-μm particle size, ∼19-cm column length), with a flow rate of 200 nl min^−1^. All samples dissolved in 0.1% formic acid were injected onto the column and eluted in a gradient from 2 to 25% acetonitrile in either 95 (for glycoproteomic samples) or 155 min (for proteomic samples), from 25 to 80% acetonitrile in 10 min, followed by isocratic elution at 80% acetonitrile for 15 min (total elution time 120 or 180 min, respectively). The nanoSpray ion source was operated at 2.1-kV spray voltage and 300 °C heated capillary temperature. A precursor MS1 scan (m/z 350–1700) of intact peptides was acquired in the Orbitrap at a nominal resolution setting of 120,000. For glycoproteomic samples, the five most abundant multiply charged precursor ions in the MS1 spectrum at a minimum MS1 signal threshold of 50,000 were triggered for sequential Orbitrap HCD MS2 and ETD MS2 (m/z of 100–2000). MS2 spectra were acquired at a resolution of 50,000 for HCD MS2 and 50,000 for ETD MS2. Activation times were 30 and 200 ms for HCD and ETD fragmentation, respectively; isolation width was 4 mass units, and 1 microscan was collected for each spectrum. Automatic gain control targets were 1,000,000 ions for Orbitrap MS1 and 100,000 for MS2 scans, and the automatic gain control for the fluoranthene ion used for ETD was 300,000. Supplemental activation (20%) of the charge-reduced species was used in the ETD analysis to improve fragmentation. Dynamic exclusion for 60 s was used to prevent repeated analysis of the same components.

For proteomic samples, the 10 most abundant multiply charged precursor ions in the MS1 spectrum at a minimum MS1 signal threshold of 100,000 were triggered for sequential Orbitrap HCD MS2 at a resolution of 60,000. In addition, for some proteomic samples, a synchronous-precursor selection MS3 method was used for quantitative analysis^55^. Polysiloxane ions at m/z 445.12003 were used as a lock mass in all runs. Raw data have been deposited to the ProteomeXchange Consortium^56^ via the PRIDE partner repository with the data set identifier PXD010155.

### Mass spectrometry data analysis

Data processing was performed using Proteome Discoverer version 1.4 software (Thermo Scientific) using Sequest HT Node as described previously^57^ with minor changes. Briefly, all spectra were initially searched with full cleavage specificity, filtered according to the confidence level (medium, low, and unassigned), and further searched with the semi-specific enzymatic cleavage. In all cases, the precursor mass tolerance was set to 6 ppm and fragment ion mass tolerance to 20 milli-mass units. Carbamidomethylation on cysteine residues was used as a fixed modification. Methionine oxidation and Hex(1)HexNAc(1), HexNAc attachment to serine, threonine, and tyrosine were used as variable modifications for ETD MS2. All HCD MS2 data were preprocessed as described^58^ and searched under the same conditions mentioned above using only methionine oxidation as a variable modification.

For the quantitative analysis, only HCD MS2 spectra were used. In the case of ETD MS2 spectra, the group of TMT reporter fragment ions (m/z range of 126–132) the quantitative data were extracted from the adjacent HCD MS2 paired spectrum (the same precursor ions), and later used for quantification. Processing of the TMT MS3 data was performed using Proteome Discoverer version 2.1 software (Thermo Scientific).

All spectra were searched against a concatenated forward/reverse human-specific database (UniProt, January 2013, containing 20,232 canonical entries and another 251 common contaminants) using a target false discovery rate of 1%. False discovery rate was calculated using the target decoy peptide–spectrum match validator node. The resulting list was filtered to include only peptides with glycosylation as a modification.

### LC-MS based Bulk Lipidomics

For lipid extraction, samples were prepared following the MTBE protocol^59^. Briefly, mouse tissues were resuspended in 100 μL of MS-grade water and 360 μL of methanol (MeOH), then homogenized using a handheld homogenizer. A mixture of internal standards (SPLASH® II LIPIDOMIX®; Avanti Polar Lipids, Inc., Alabama, USA) was added. Subsequently, 1.2 mL of MTBE was added, and the samples were vortexed vigorously at maximum speed for 1 h at 4 °C.

Phase separation was induced by adding 200 μL of MS-grade water, followed by centrifugation at 1000 × *g* for 10 min. The upper (organic) phase was collected, and the lower (aqueous) phase was re-extracted with 400 μL of a MTBE/MeOH/H₂O mixture (10:3:1.5, v/v/v). This extraction was repeated once more. The combined upper phases were dried under a nitrogen stream and stored at −80 °C.

Eluents consisted of 5 mM ammonium acetate and 0.1% formic acid in water (solvent A), and in isopropanol/acetonitrile (2:1, v/v) (solvent B). Dried lipid extracts were resuspended in a mixture of 20% LC-MS-grade chloroform/methanol (1:1, v/v) and 80% solvent B. Lipid analyses were performed using a Shimadzu Prominence UFPLC XR system (Tokyo, Japan) equipped with a reversed-phase Accucore C30 column (150 × 2.1 mm, 2.6 μm) and a 20 mm guard column (Thermo Fisher Scientific), coupled to a hybrid Orbitrap Elite mass spectrometer (Thermo Fisher Scientific, Bremen, Germany). The instrument was operated in positive ion mode using a heated electrospray ionization (HESI) source.

Lipid quantification was conducted using Skyline (v. 21.1.0.146; MacCoss Lab, University of Washington, Seattle, USA). A transition list was initially generated using LipidCreator, a free and open-source tool integrated with Skyline, and subsequently modified based on experimental data. The transition list included the lipid molecule name, precursor name, precursor m/z, chemical formula, and adduct. Peak areas were normalized to the corresponding internal standards and expressed as a percentage of the total lipid content (mol%).

### GSL extraction and analysis

For lipid extraction, cells or tissues were harvested into glass tubes (13 × 100 mm) containing 1.5 mL of methanol (Honeywell, Germany). The solution was homogenized using a sonic probe (approximately 20 strong pulses). Mass spectrometry (MS) standards (Avanti Polar Lipids, Alabaster, AL, USA; and Cayman Chemical Company, Ann Arbor, MI, USA) were then added to the samples prior to total lipid extraction.

Following sonication, 0.75 mL of chloroform (Honeywell, Germany) was added. The solution was briefly homogenized again with the sonic probe and left to extract overnight at room temperature. After extraction, the samples were briefly sonicated once more, placed in a heating box, evaporated under a gentle stream of nitrogen (N₂), and then reconstituted in 400 µL of a methanol/chloroform (1:1) mixture. The samples were subsequently transferred to vials, centrifuged, and analyzed via HPLC-MS/MS.

### Liquid chromatography separation and tandem mass spectrometry

Sphingolipid species (excluding gangliosides) were separated by reversed-phase HPLC, as previously described^60^, using a Dionex Ultimate 3000 system (Thermo Scientific, USA) with a Gemini C18 column, 5 µm, 250 × 4.6 mm (Phenomenex, USA), at a flow rate of 0.7 mL/min. A gradient of mobile phases RA (methanol/water, 60:40) and RB (methanol) was used, starting at a 60:40 ratio, with a total run time of 69 min. Formic acid and ammonium formate were used as eluent additives.

Glucosylceramide and galactosylceramide were separated by normal-phase HPLC on a Spherisorb 5 µm Silica column, 2.1 × 250 mm (Waters Corporation, Ireland), using a Dionex Ultimate 3000 system (Thermo Scientific, USA). The flow rate was 0.3 mL/min, with a gradient of mobile phases NA (acetonitrile/methanol, 99:1) and NB (acetonitrile/methanol/water, 40:47:13), and a total run time of 48 min. Formic acid and ammonium formate were used as eluent additives.

Gangliosides were separated by reversed-phase HPLC on an ARION® C8 column, 4.6 × 300 mm (Chromservis s.r.o., Czech Republic), at a flow rate of 0.8 mL/min. A gradient of mobile phases RA (water/methanol, 90:10) and RB (methanol/isopropanol, 1:1) was applied, starting at a 61:39 ratio, with a total analysis time of 60 min. Ammonium formate was used as the eluent additive.

Tandem mass spectrometry was performed using a hybrid triple quadrupole QTRAP 4500 system (AB Sciex, Canada), operating in positive electrospray ionization (ESI) mode. Drying gas, collision energy, and fragmentor voltage were optimized for each species. The instrument was run in multiple reaction monitoring (MRM) scan mode.

### Bone microarchitecture

To evaluate bone microarchitecture, a SkyScanner 1276 (Bruker, Belgium) was used with a 0.25 mm filter, voltage of 200 kV and a current of 55 mA. Samples were wrapped in PBS-soaked paper towels and scanned inside a drinking straw sealed on both ends to avoid drying. Voxel size was set at 10 × 10 × 10 μm^3^. Bone microarchitecture was evaluated according to the ASBMR guidelines^61^ using a custom CTan (Bruker, Belgium) script for automatic segmentation of trabecular bone in the distal femoral and proximal tibia VOIs. To account for the marked differences in bone length between the groups, we set the number of slices and the offset with respect to the anatomical landmarks relative to bone length instead of using an absolute number.

The trabecular VOI for femora was defined as the region spanning 15% of the bone length at 7.5% of the bone length away from the distal metaphysis, which resulted in a VOI of about 200 slices with a 100 slice distance from the distal metaphysis for wild-type animals, and VOI of 150 slices 75 slices away from the metaphysis for the GOLPH3-KO mice. The cortical VOI for the femora spanned 10% of the femoral length centered around the midshaft slice, resulting in a VOI of about 130-140 slices for wild-type animals and 100 slices for GOLPH3-KO mice.

Similarly, the trabecular VOI for tibias was defined as the region spanning 12.5% of the bone length at 2.5% of the bone length away from the proximal metaphysis, which resulted in a VOI of about 200 slices with a 40 slice distance from the distal metaphysis for wild-type animals, and VOI of 150-160 slices 30 slices away from the metaphysis for the GOLPH3-KO mice. The cortical VOI for the tibia spanned 5% of the femoral length centered around the slice 20% of the bone length away from the tibio-fibular junction. This results in VOI of about 80 slices 320 slices away from the anatomical landmark for wild-type mice and 60 slices 250 slices away for GOLPH3-KO animals.

The threshold used to binarize the calcified tissue was 40 on a 0-255 scale for trabecular bone and 110 for cortical. Reconstruction of the scans was performed using NRecon (Bruker, Belgium) and further analysis were performed using CTan (Bruker, Belgium) with the minimum for CS to image conversion set at 0 and maximum set at 0.14.

### Molecular dynamics simulations

All MD simulations were set up with the CHARMM-GUI^62,63^ server produced with Gromacs 2022 or 2024, and analyzed in VMD assisted with custom scripts plus tools of the WebMDA app at the PDB Manipulation Suite (^2^). Within CHARMM-GUI, coarse-grained systems were parametrized with the MARTINI 2.2p^64^ forcefield starting from the GOLPH3 crystal structure PDB 3KN1^12^.

For CG simulations aimed at probing spontaneous binding of GOLPH3 to various membranes, the protein was initially positioned such that its closest bead was 28-30 Å from the nearest membrane bead. The membrane was composed of a mixture of approximately 30% POPC, 20% DOPC, 12% POPE, 7% DOPE, 5% POPS, and 16% cholesterol and a variable lipid component consisting of either 0%, 5%, 10%, 20%, or 40% PtdIns(4)*P*, or 10% PtdIns(4,5)P₂, CL, or PS, as explained for each simulation and in each case proportionally reducing the fractions of the first set of lipids. All lipid compositions are expressed as mol%. The membrane was parameterized with standard MARTINI 2 lipids, and the system was solvated with the polarizable water and 150 mM NaCl. Each system was minimized and equilibrated with the standard CHARMM-GUI protocol, that is by slowly removing restraints in the NVT ensemble using Particle Mesh Ewald for the electrostatic contributions and velocity rescale for temperature coupling up to 310 K. The production phase was carried out in the NPT ensemble using a Parrinello-Rahman semiisotropic coupling algorithm for maintaining the pressure constant at 1 bar, 310 K as the target temperature, and 20 fs integration time steps. Five independent replicas were run for all simulations.

For CG simulations of GOLPH3 S-acylated at C84 on the membrane, we added the 4 beads that represent the palmitate group in the MARTINI 2.2p framework extending away from the C84’s sidechain bead, and applied the parameters optimized in ref. ^65^.

For CG simulations of the GOLPH3 – LCS complex on the membrane, we took the NMR-consistent model of GOLPH3 and LCS generated with AlphaFold-Multimer and extended the LCS towards its C-terminus with a canonical helix of the corresponding amino acid sequence, projecting into the membrane. The parametrization of this system proceeded as explained above, including the same procedure to model the acylated form of C84; and the system was minimized, equilibrated and produced in MD with the same protocols.

### Generation of stable cell lines expressing LCS-SI-GFP

The LCS-SI-GFP plasmid was previously used and described in ref. ^10^. HeLa cells were transfected with the plasmid using JetPrime transfection reagents (Polyplus) following the manufacturer’s instructions. Stable clones were selected using 800 μg/mL of G418 antibiotic. Expression of the GFP-tagged proteins in the selected clones was confirmed by Western blot analysis using an anti-GFP antibody (Ref 11814460001, Roche).

### Generation of GOLPH3-KO/LCS-SI-GFP clones

GOLPH3-KO cell line was generated from stable LCS-SI-GFP HeLa cells using CRISPR/Cas9 technology. These cells were transfected with GOLPH3 CRISPR plasmids (sc-412973, Santa Cruz Biotechnology) by using JetPrime transfection reagents (Polyplus) following the manufacturer’s instructions. Single-cell clones were isolated by limiting dilution in 96-well plates. The resulting clones were screened for GOLPH3-KO by Western blot analysis using an anti-GOLPH3 antibody (ab98023, Abcam).

### Acyl-Resin Assisted Capture (Acyl‑RAC) assay

Protein S-acylation was assessed by the Acyl‑RAC assay as described by ref. ^66^, with some modifications. HeLa or HEK-293 cells were lysed with a buffer containing 0.5% Triton-X100, 100 mM Hepes pH 7.4, PBS, 1 mM EDTA, 0.2 mM SDS and protease inhibitor cocktail). In order to block free SH groups with 1 M N-Ethylmaleimide (NEM), 200 ml of blocking buffer (100 mM Hepes, 1 mM EDTA, 87.5 mM SDS and 50 mM NEM) was added to cell lysate and incubated for 2 hours at 40 °C. Subsequently, 3 volumes of ice-cold 100% acetone was added to the blocking protein mixture and incubated for 20 min at 20 °C and then centrifuged at 5000 g for 10 min at 4 °C to pellet precipitated proteins. The pellet was washed five times in 1 ml of 70% (v/v) acetone and resuspended in a buffer (100 mM Hepes, 1 mM EDTA, 35 mM SDS). For treatment with hydroxylamine (HA) and capture by Thiopropyl Sepharose beads, 2 M HA was added together with the beads (previously activated for 15 min with water) to a final concentration of 0.5 M HA and 10% (w/v) beads. As a negative control, 2 M Tris was used instead of HA. These samples were then incubated for 3 h at room temperature on a rotating wheel. The beads were washed, the proteins were eluted from the beads by incubations in 40 μl SDS sample buffer with beta-mercapto-ethanol for 5 min at 95 °C. Finally, samples were submitted to SDS-PAGE and analysed by immunoblotting. The same protocol was used to identify the palmitoyltransferases responsible for GOLPH3 S-acylation through an siRNA screening of ZDHHC enzymes. HeLa cells were transfected with siRNAs against six different mixes of ZDHHC enzymes. Mix 1 included ZDHHC 1, 3, 7, 13, and 17, Mix 2: ZDHHC 2, 4, 6, and 16, Mix 3: ZDHHC 5, 8, 9, and 20, Mix 4: ZDHHC 12, 15, and 18, Mix 5: ZDHHC 11, 23, and 24, and Mix 6: ZDHHC 14, 19, 21, and 22.

### PEGylation assay

The level of protein S-palmitoylation was assessed as described in ref. ^67^, with minor modifications. HeLa or HEK-293 cells were lysed with the following buffer 0.5% Triton-X100, 100 mM Hepes pH 7.4, PBS, 1 mM EDTA, 0.2 mM SDS and protease inhibitor cocktail. After centrifugation at 100,000 g for 15 min, supernatant proteins were reduced with 25 mM TCEP for 30 min at room temperature, and free cysteine residues were alkylated with 1 M NEM for 2 h at room temperature to be blocked. After chloroform/methanol precipitation, resuspended proteins in PBS with 4% SDS and 5 mM EDTA were incubated in buffer (1% SDS, 5 mM EDTA, 1 M NH2OH, pH 7.0) for 1 h at 37 °C to cleave palmitoylation thioester bonds. As a negative control, 1 M Tris-HCl, pH 7.0, was used. After precipitation, resuspended proteins in PBS with 4% SDS were PEGylated with 20 mM mPEG-5 for 1 h at 37 °C to label newly exposed cysteinyl thiols. After precipitation, proteins were resuspended with SDS sample buffer and boiled at 95 °C for 5 min.

Detection of protein S-acylation by mPEG Click-chemistry is adapted from JBC (2020) 295(21)7501-7515. Cells are washed twice with serum-free medium with 1 mg/ml BSA and incubated indicated times with either 100 µM palmitic acid azide or 100uM unlabeled palmitic acid (control). Cells are washed 3 times with cold PBS and lysed in 50 mM Tris-HCl (pH 8.0) and 0.5% SDS. Cell lysates are subjected to click reaction with 5 kDa mPEG-Alkyne, CuSO4, TBTA and ascorbic acid for 1 h at room temperature. Samples are boiled with 4x SDS with BME sample buffer, and analyzed by WB on reducing condition on 4–20% SDS-PAGE gels.

### Radiolabeling experiments

To detect palmitoylation, HeLa WT or GOLPH3-KO cells were transfected or not with different GOLPH3 constructs, incubated for 3 h in IM (Glasgow minimal essential medium buffered with 10 mM Hepes, pH 7.4) with 200 mCi/ml ^3^H palmitic acid (9,10-^3^H(N)) (American Radiolabeled Chemicals, Inc.). The cells were washed and directly lysed for immunoprecipitation with the anti-GOLPH3 rabbit antibody. For all radiolabeling experiments, after immunoprecipitation, washed beads were incubated for 5 min at 90 °C in reducing sample buffer prior to 4–12% gradient SDS-PAGE. After SDS-PAGE, the gel was incubated in a fixative solution (25% isopropanol, 65% H_2_O, 10% acetic acid), followed by a 30-min incubation with signal enhancer Amplify NAMP100 (GE Healthcare). The radiolabeled products were revealed using Typhoon phospho-imager and quantified using the Typhoon Imager (ImageQuanTool, GE Healthcare). Quantification of radioactive experiments was quantified using specific screens by autoradiography. The images shown for ^3^H-palmitate labeling were however obtained using fluorography (indirect detection of radioactive emission by stimulated light emission from a fluorophore (Amplify) on film).

### Reporting summary

Further information on research design is available in the Nature Portfolio Reporting Summary linked to this article.

## Supplementary information

Supplementary information

Description of Addtional Supplementary Files

Supplementary Data 1

Supplementary Data 2

Supplementary Data 3

Supplementary Data 4

Supplementary Data 5

Reporting Summary

Transparent Peer Review file

## Source data

Source Data1

Source Data2

Source Data3

Source Data4

Source Data5

## Data Availability

The transcriptomics data generated in this study have been deposited in the GEO database (https://www.ncbi.nlm.nih.gov/geo/) under accession code GSE327245. Lipidomics data are available in the form of supplementary files. Proteomics and gyco-proteomics data are available at the Proteomics Identification Database PRIDE under the accession code PXD064301; token BzUmwnW1EveT. The partial backbone assignments for human GOLPH3 are available at the BMRB under the ID 53516. Source data are provided with this paper.
