# Supplementary material for: Molecular regulation and physiological role of GOLPH3-mediated Golgi retention
Source: Nat Commun. 2026 Jun 11;17:7426. doi: 10.1038/s41467-026-74133-6 (PMC13408441; doi:10.1038/s41467-026-74133-6)
Supplement: Supplementary file 1 — Supplementary information [file 41467_2026_74133_MOESM1_ESM.pdf]

Supplemental Material: Molecular Regulation and Physiological Role of GOLPH3-mediated Golgi retention

Table of contents

1. Supplemental Methods
2. Supplemental Figures and Figure Legends

1. Supplemental Methods

SDS-PAGE and Western Blotting

Proteins from different tissues were extracted using mammalian protein extraction reagent (MPER, Pierce) supplemented with halt phosphatase and halt protease inhibitors (Pierce) as per the manufacturer's instructions, separated by gel electrophoresis and analyzed using the antibodies anti-GOLPH3/MIDAS (ab98023, Abcam), anti-Calnexin (MAB3126, Millipore) and anti-α-tubulin (ab18027, Abcam).

RTqPCR

Tissues were powdered manually in liquid nitrogen. RNA was isolated with Tri-Reagent (T9424, Sigma-Aldrich). After centrifugation to remove debris (12,000 g, 10 min, 4°C), chloroform was used for phase separation. The aqueous phase was recovered and precipitated with isopropanol, and centrifuged (12,000 g, 10 min, 4°C), and then, RNA pellets were washed with 75% ethanol. Pellets were resuspended in Milli-Q water. RNA concentrations were determined using NanoDrop and reverse-transcribed using 1,000 ng of RNA and Superscript II enzyme (18064014, Invitrogen) according to the manufacturer's instructions. qPCR analysis was performed using SYBR Green detection (04913914001, Roche) on a 7900HT Fast Real-Time PCR System (Applied Biosystems) according to the manufacturer's instructions. Relative mRNA expression was calculated from the comparative threshold cycle (Ct) values of the gene of interest relative to RS9 mRNA.

The specific primer sequences that were used are as follows:

Golph3 (F′-ATAGAATTCATTATGACCTCGCTGACCCAGCGCA, R′-ATAGGATCCATTTTACTTGGTGAACGCCGCCACCAT) and Rs9 (F′-CGGCCCGGGAGCTGTTGACG, R′-CTGCTTGCGGACCCTAATGTGACG).

RNA Sequencing

Total RNA was isolated from the pulverized liver tissues by following the manufacturer’s protocol (RNeasy, Qiagen). The extracted RNA amount was quantified by using a spectrophotometer (Thermo Scientific NanoDrop One). Subsequently, RNA quality was measured by using the TapeStation 4200 system (Agilent). Four WT or four GOLPH3-KO RNA samples having RNA integrity score at least 7 was used for further bulk RNA sequencing.

Library prep and sequencing were performed in the Gene Expression Core Facility at EPFL, Switzerland. A Watchmaker mRNA library prep, starting from 500 ng of RNA was performed according to Watchmaker mRNA Library Prep Kit, Version 2.2.1224. Resulting libraries were quantified by qubit DNA HS and profile analysis was done by TapeStation 4200 (Agilent). Paired end (2 × 75 bp) sequencing was performed on the AVITI24 platform, producing approximately 1 billion reads per run. Reads in FASTQ format were generated by using Sanger/Illumina 1.9, including trimming by Bases2Fastq v2.3.0.

Reads in FASTQ format were aligned to the mouse reference genome GRCm39 with Ensembl release 115 gene annotations using STAR v2.7.11b in 2-pass mode. Gene-level read counts were quantified using featureCounts v2.1.1 (Subread) with reverse-strand specificity, counting paired reads with both mates mapped and excluding chimeric fragments. The differential gene expression analysis was performed by DESeq2 package on R Studio.

Tissue Staining

Serial 4 μm paraffin sections were prepared using a rotary microtome and stained with either standard Hematoxylin and Eosin (H&E) to assess general tissue morphology or Alcian Blue (pH 2.5) to visualize cartilage. All staining procedures were performed using a Tissue-Tek Prisma® Automated Slide Stainer (Sakura).

For H&E staining, tissue sections were deparaffinized in xylene, rehydrated through graded ethanol to distilled water, and stained with Harris Hematoxylin solution (Gill II, PAP1; Bio-Optica) for 5 minutes. After rinsing in tap water, sections were briefly differentiated in 1% acid-alcohol (700 mL absolute ethanol, 10 mL 37% HCl, 290 mL H_2O), followed by a 10-minute wash in running tap water for nuclear blueing. Cytoplasmic structures were stained with a working solution of Eosin-Phloxine for 1 minute, prepared by combining 50 mL of 1% Eosin Y

(Sigma, E4382), 5 mL of 1% Phloxine B (Fluka, 28550), 360 mL of 95% ethanol, and 2 mL of glacial acetic acid. Slides were then washed in distilled water, dehydrated, cleared in xylene, and mounted using Eukitt mounting medium (Sigma-Aldrich).

For Alcian Blue staining, sections were deparaffinized, rehydrated, and incubated for 25 minutes at room temperature in 1% Alcian Blue (Sigma-Aldrich, A5268) dissolved in 3% acetic acid (pH 2.5). The solution was filtered prior to use and prepared by dissolving 5 g of Alcian Blue in 500 mL of 3% glacial acetic acid. After staining, slides were washed in running tap water for 5 minutes and briefly rinsed in distilled water. Nuclei were counterstained with 0.1% nuclear fast red (Vector Laboratories, H-3403) for 5 minutes, followed by rinsing, dehydration, clearing, and mounting with Eukitt. Acidic mucopolysaccharides and mucins stained blue, while nuclei appeared red.

Immunofluorescence and confocal microscopy

HeLa GOLPH3-KO cells transfected with the different GOLPH3 constructs were plated at approximately 50% confluency post-transfection. Cells were washed with PBS, fixed in 4% paraformaldehyde (PFA) for 20 minutes, washed with PBS, quenched with 50 mM NH_4Cl for 10 minutes, and washed again with PBS. Cells were permeabilized with 0.05% saponin in PBS for 5 minutes. Subsequently, cells were washed and blocked with 3% BSA and 0.05% saponin in PBS for 30 minutes. Primary antibodies (anti-GOLPH3 and anti-GM130) and secondary antibodies were diluted in the same buffer. After blocking, coverslips were washed and incubated with primary antibodies overnight at 4 °C, washed in PBS, and incubated for 45 minutes with secondary antibodies. Cells were then washed and stained with Hoechst for nuclear visualization. Finally, coverslips were mounted in Prolong Glass Antifade Mountant. Images were acquired using a Zeiss LSM 980 Inverted microscope with a 63X objective, a pinhole size of 1 AU and at least 2 line-averaging or alternatively using using Oympus Evident FV4000. Super-resolution images were taken using Zeiss LSM980 with Airyscan2 module, with 63×oil-immersion objectives. Images were processed using Fiji (ImageJ). Cis-to-trans Golgi linescans were recorded using the “Color Profiler” plugin (Color_Profiler. jar, https://imagej.nih.gov/ij/plugins/color-profiler.html) in Fiji software.

LCS-SI-GFP retention assay

HeLa GOLPH3-KO/LCS-SI-GFP cells were transfected with the different GOLPH3 constructs. Forty-eight hours after transfection, cells were harvested by trypsinization, fixed 10 minutes in 4% PFA/PBS, washed, and resuspended in PBS containing 1% BSA.

To assess plasma membrane (PM) localization of luminal GFP chimeras containing the transmembrane domain of sucrase–isomaltase and the corresponding N-terminal LCS cytosolic tails, cells were incubated with 4 µg/ml anti-GFP mouse monoclonal antibody (Ref. 11814460001, Roche) and 4 µg/ml of anti-mouse Alexa Fluor 568 (A-10037, Thermo Fisher Scientific) for 1 hour at RT without permeabilization.

After incubation, cells were extensively washed with PBS/1% BSA, fixed 10 minutes in 4% PFA/PBS again and washed. For selective detection of cells expressing GOLPH3 constructs, a rabbit polyclonal anti-GOLPH3 antibody (2 µg/ml; Abcam, ab98023) and an anti-rabbit Alexa Fluor 647 (2 µg/ml; A-31573 Thermo Fischer Scientific) were applied in permeabilization conditions (PBS/1% BSA and 0,05% Saponin) for 1 hour at RT. Cells were then washed and stained with Hoechst for nuclear staining.

Negative controls included cells stained with secondary antibodies only and unstained cells. Data acquisition was performed using an LSRII SORP flow cytometer (BD Biosciences).

Bacterial protein expression and purification

The human GOLPH3 (Uniprot sequence Q9H4A6, residues 52-298) expression plasmid carries the gene for the GOLPH3 protein lacking the first 51 amino acids, with an N-terminal 10xHistidine tag and a Twin-Strep-tag, followed by a TEV (Tobacco Etch Virus) cleavage site. GOLPH3 was cloned into the bacterial expression vector pET_HST and mutations were introduced using the GenScript's Site-Directed Mutagenesis Services (GenScript). The coding sequences for all GOLPH3 variants, except for the GOLPH3 WT and GOLPH3 D247A mutants, were optimized for Escherichia coli (*E. coli*) expression (Genscript). The plasmids were transformed into BL21(DE3) competent cells. Protein expression was induced with 0.5 mM isopropyl b-D-1-thiogalactopyranoside (IPTG) when cells reached an optical density (OD at 600 nm) of 0.6, with a subsequent growth overnight at 20 °C. Cell pellets were resuspended in lysis buffer (500 mM NaCl, 50 mM Tris pH 8.0, 1 mM TCEP, 10% glycerol) and were lysed by sonication. After centrifugation at 20,000 xg for 60 minutes at 4 ˚C, the supernatant was filtered and applied to a HisTrap excel column (Cytiva) at room temperature. After elution of recombinant GOLPH3 with a continuous gradient over 30 column volumes of elution buffer (500 mM NaCl, 50 mM Tris pH 8.0, 1 mM TCEP, 10% glycerol, 500 mM imidazole), pure fractions were buffer exchanged into final buffer (250 mM NaCl, 50 mM Tris pH 8.0, 1 mM TCEP) to remove imidazole, using a HiPrep Desalting column (GE Healthcare). The N-terminal purification tags were cleaved by overnight incubation with TEV protease at room temperature. Subsequently, the cleaved tags and TEV protease were removed with another His affinity chromatography and the flowthrough was collected, concentrated, and additionally purified by Size Exclusion Chromatography (HiLoad 16/600 Superdex 200 pg, Cytiva) in final

buffer. Pooled fractions were concentrated to the required concentration using a MWCO concentrator (Millipore) with a cutoff of 10 kDa.

Size exclusion chromatography coupled to multi-angle light scattering

The molecular weights of the tested constructs were determined by size exclusion chromatography coupled to multi-angle light scattering (SEC-MALS). The mass measurements were performed on a UltiMate3000 HPLC system equipped with a 3 angles miniDAWN TREOS static light scattering detector (Wyatt Technology). The sample volumes of 5−10 µl at a concentration of 12 mg/mL, were applied to a Superdex 200 5/150 GL column (Cytiva) previously equilibrated with 250 mM NaCl, 50 mM Tris pH 8.0, 1 mM TCEP at a flow rate of 0.08 mL/min. The data were analyzed using the ASTRA 6.1 software package (Wyatt technology), using the absorbance at 280 nm and the theoretical extinction coefficient for concentration measurements.

Nuclear Magnetic Resonance (NMR) Spectroscopy

All proteins used for NMR experiments were designed on the GOLPH3-4G background due to its higher stability and solubility. Recombinant proteins were prepared with the isotope enrichment scheme appropriate for each experiment (i.e. ^{15}N-only or ^{15}N, ^{13}C) in 20 mM MES buffer pH 6.5 with 250 mM NaCl and 10% $^{2}H_2O$. Lower salt, even by 10%, yielded much degraded spectra. Protein concentrations were around 300 µM for all NMR experiments, limited by protein stability and solubility, which as described in the text was optimal at around 35 Celsius, each sample lasting for 1.5-2 days and then substituted by freshly prepared sample when required.

All NMR experiments were carried out in a 18.8 T (800 MHz ^{1}H Larmor frequency) Bruker spectrometer equipped with a CPTC ^{1}H, ^{13}C, ^{15}N 5 mm cryoprobe and an Avance Neo console. The temperature was set to 308 K in all experiments, as spectra were substantially better than at the more standard 298 K yet without compromising the limited stability. Backbone (H, N, CA, and C) and CB resonances were assigned using a standard procedure based on conventional 3D HNCA, HN(CO)CA, HNCO, HN(CA)CO, CBCA(CO)NH and HNCACB spectra. All spectra were acquired and processed using Bruker's Topspin 4.0 software. Backbone assignments were obtained through manual spectral analysis assisted by the program CARA. As explained, the protein assignment is only partial, and assisted by HSQC spectra acquired on single-point mutants. The NMR chemical shifts for all confidently assigned residues are provided in **Table S1** and deposited in the BMRB under ID 53516.

^{15}N T_1 and T_2 to probe protein oligomerization state in solution were recorded with the regular sensitivity-enhanced pseudo-3D versions of the pulse programs available off the shelf in Topspin 4 for the Avance Neo console. They were measured with 128 ^{15}N increments processed with 256 points and a total recycle time of 4 seconds, with the following delays: 8, 64 (twice), 232, 472, 664, 800 (twice), 1000, 1200, 1600 and 2000 ms (for T_1) and 16, 32 (twice), 64, 96, 128, 160, 192 (twice), 224, 256 and 288 ms (for T_2). Spectra were acquired and processed in Bruker TopSpin and then analyzed with the relaxation module in Sparky-NMRFAM. Since NMR measurements on GOLPH3 were only feasible at 308 K but calibration curves relating NMR-determined correlation times to protein molecular weight are available at 298 K, we adjusted the fitted relaxation parameters to derive values at 298 K. Briefly, the data obtained at 308 K showed an average and median T_2 across all measurements of 51 and 38 ms, respectively, while the average and median T_2 across residues with confirmed assignments are 49 and 35 ms. For T_1 the average and medians are 1150 and 1200 ms (all residues) and 1180 and 1200 ms (assigned residues). These values yield central R_2/R_1 ratios in the range 28-31.5 (unitless) which corresponds to global correlation times (τ_c) in the range 12.6-13.4 ns according to the formula linking these values[68]. Direct interpolation would suggest a molar mass of around 21 kDa, which is under the molar mass expected for monomeric Golph3 (28.2 kDa). However, adjusting the correlation time from 308 to 298 K by using not only the temperatures but also the tabulated viscosity coefficients for water (0.89 mPa s at 298K and 0.72 mPa s at 308K) into the ratio of the Stokes-Einstein-Debye equation, results in a correlation time estimated around 16.2 – 17.2 ns at 298 K. On calibration curves at this temperature, this matches a molecular weight of 25.9-27.4 kDa which fits much better with the molecular weight expected for the monomeric protein as prepared (28.2 kDa).

Residue	H	N	CA	CB	C	CSP (H,N)
G76	7.7302	104.4004	43.1271			0
Y77	7.3368	120.7746	52.6107	39.3925		0
G195	8.2864	108.7969	42.6103			0
G196	8.3448	108.9055	42.6175			0
G197	8.3115	108.75	42.6223		171.2667	0
D198	8.2825	120.2743	51.655	38.5638	173.8144	NA

M199	8.4254	120.4332	53.0886	29.9318	173.8309	NA
T200	8.2657	114.4234	59.7786	67.1529	171.9781	0
T201	8.0514	115.3135	58.8964	67.4606		0
H202	8.2315	122.0959	51.5815	27.7911	170.2667	0
A245	7.5637	119.252	51.9491	16.8112	175.965	0.023
S246	7.0681	102.7262	58.198		171.6638	0.080056
D247	7.6041	123.2618	51.7653			0.054781
D258	8.5439	120.8278	55.6249	37.8208	174.9889	0.096664
E259	9.1364	118.5201	57.1687	26.2028	176.7921	0.069971
Q260	7.3913	117.8191	56.1395	26.6791	174.1618	0.120814
Y261	8.7091	121.7632	59.9624			0.103942
D262	8.3054	120.0985	54.8162			NA
L263	7.2088	120.1409	54.8897			NA
T265	7.6116	107.8329	63.1971	66.048		0.115741
K266	7.7896	123.9872	57.132	29.3794	176.7756	0.108167
V293	7.949	117.3537	65.1085			NA
A294	8.5961	122.1431	52.5004	14.8086	176.6763	0.023324
A295	8.1231	120.1343	51.655	14.6878	176.1139	NA
F296	7.7406	114.0837	56.0292	37.0001	173.9136	0.023324
T297	7.8556	114.0999	60.5505	67.4982	171.1509	0.124724
K298	7.902	129.6113	54.853	30.708	178.7442	0.096519

Table S1. Backbone chemical shifts (in p.p.m.) for Golph3-4G and Chemical Shift Perturbations (in p.p.m) by the final titration point. As explained in the methods these values are all in MES pH 6.5, 250 mM NaCl, 308K.

Isothermal titration calorimetry

Isothermal titration calorimetry (ITC) experiments were performed on a MicroCal PEAQ-ITC (Malvern Panalytical), with GOLPH3 variants being in a buffer containing 250 mM NaCl, 50 mM Tris pH 8.0 and 1 mM TCEP. LCS peptides were synthesised and delivered as lyophilised powder (Genscript). The peptides were dissolved in the protein buffer and their concentrations were calculated based upon their absorbance at 280 nm and their corresponding molar extinction coefficient. Experiments consisted of titrations of a single 0.4 µL injection followed by 19 injections of 2 µl of LCS peptides, at approximately 2.5 mM, into the cell containing GOLPH3 proteins at a 100 µM concentration. Experiments were performed at 25 °C in triplicate (n = 3), with injection duration of 4 s, injection spacing of 150 s, stir speed of 750 rpm, and reference power of 7 µcal/s. Data were processed and plotted using MicroCal PEAQ-ITC Analysis Software and fitted to a one-site binding model.

Circular Dichroism Spectroscopy

Circular Dichroism Spectra of GOLPH3 proteins (at 10 µM in 100 mM NaCl, 50 mM Tris pH 7.4 and 1 mM TCEP) were collected with a Chirascan V100 (AppliedPhotophysics), using a 1 mm path length cuvette, at 20 °C, in the 200-260 nm range. Thermal denaturation experiments were performed by heating the sample from 20 to 95 °C and collecting CD data at 207 nm every 1 °C, with a temperature ramp speed of 1 °C/min and an equilibration time of 15 seconds.

Table S2

	**WT**	**G4**	**D247A**	**D247R**	**DDDAAA**	**DDDRRR**
% alpha	41	36	36	38	40	38
% beta-antiparallel	7	13	8	0	0	7
% beta parallel	0	0	0	0	0	0
% turn	16	15	12	10	13	15
% others	36	36	44	52	47	40

Table S2: Fits of CD spectra to fractions of secondary structure for Golph3 WT and various mutants of it, carried out with the BestSel server

2. Supplemental Figures and Figure Legends

Figure S1

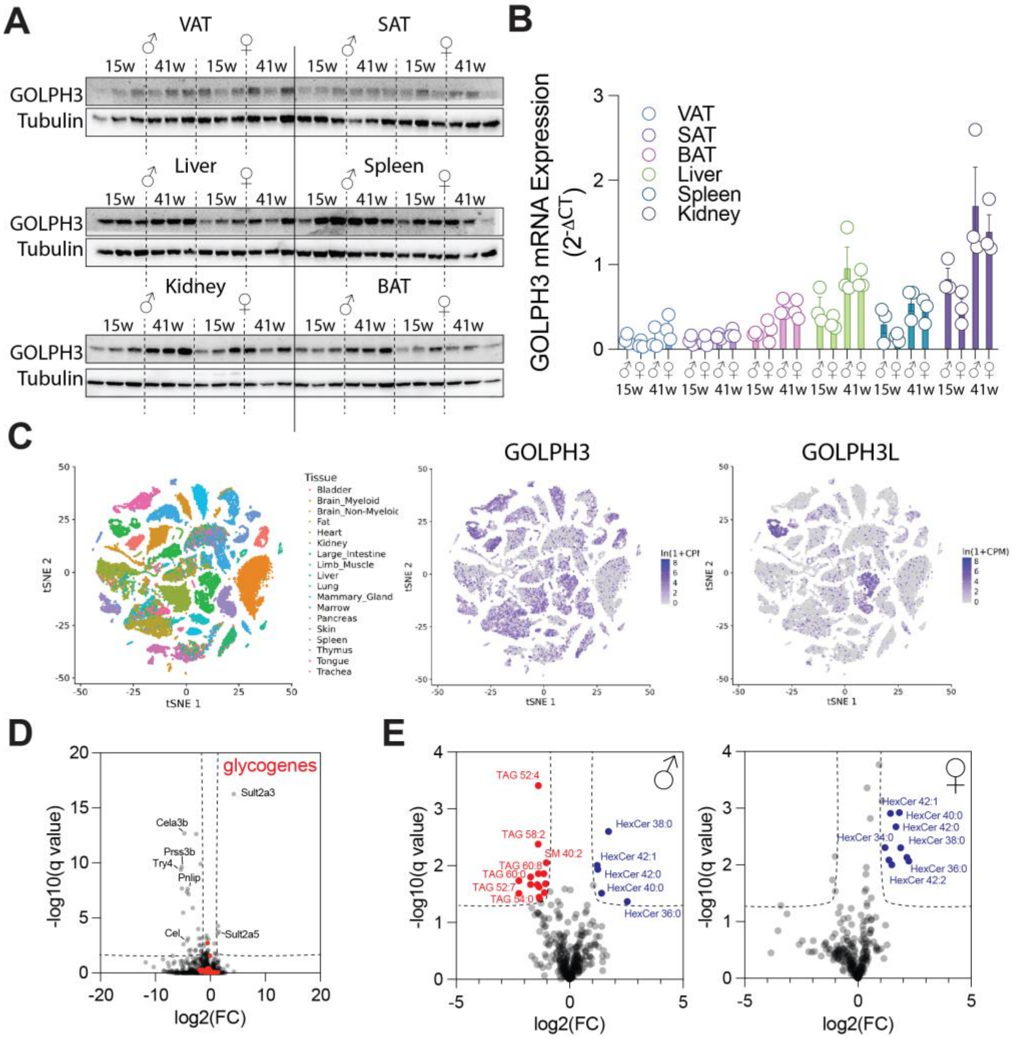

Figure S1: GOLPH3-KO causes glycosylation defects *in vivo*

A. Western blot analysis of lysates from visceral adipose tissue (VAT), subcutaneous adipose tissue (SAT), liver, spleen, kidney, and brown adipose tissue (BAT) from young (15 weeks) and old (41 weeks) male and female mice. GOLPH3 and tubulin (loading control) protein levels were detected using specific antibodies.

B. Quantitative PCR (qPCR) analysis of GOLPH3 mRNA levels in VAT, SAT, liver, spleen, kidney, and BAT from the same mouse cohorts.

C. t-SNE plot derived from the Tabula Muris single-cell RNA sequencing dataset[22], showing the expression patterns of GOLPH3 and GOLPH3L across multiple organs and tissues.

D. Volcano plot illustrating genes that differ significantly in abundance between livers of GOLPH3$^{+/+}$ and GOLPH3$^{-/-}$ mice (n = 4 per group), based on RNA sequencing. Genes encoding for components of the glycosylation machinery (glycogenes) are shown in red.

E. Volcano plot illustrating lipid species that differ significantly in abundance between livers of GOLPH3$^{+/+}$ and GOLPH3$^{-/-}$ mice (n = 5 per group), based on liquid chromatography–mass spectrometry (LC-MS)–based lipidomics. Lipid species reduced in GOLPH3$^{-/-}$ livers are shown in red; those increased are shown in blue.

Figure S2

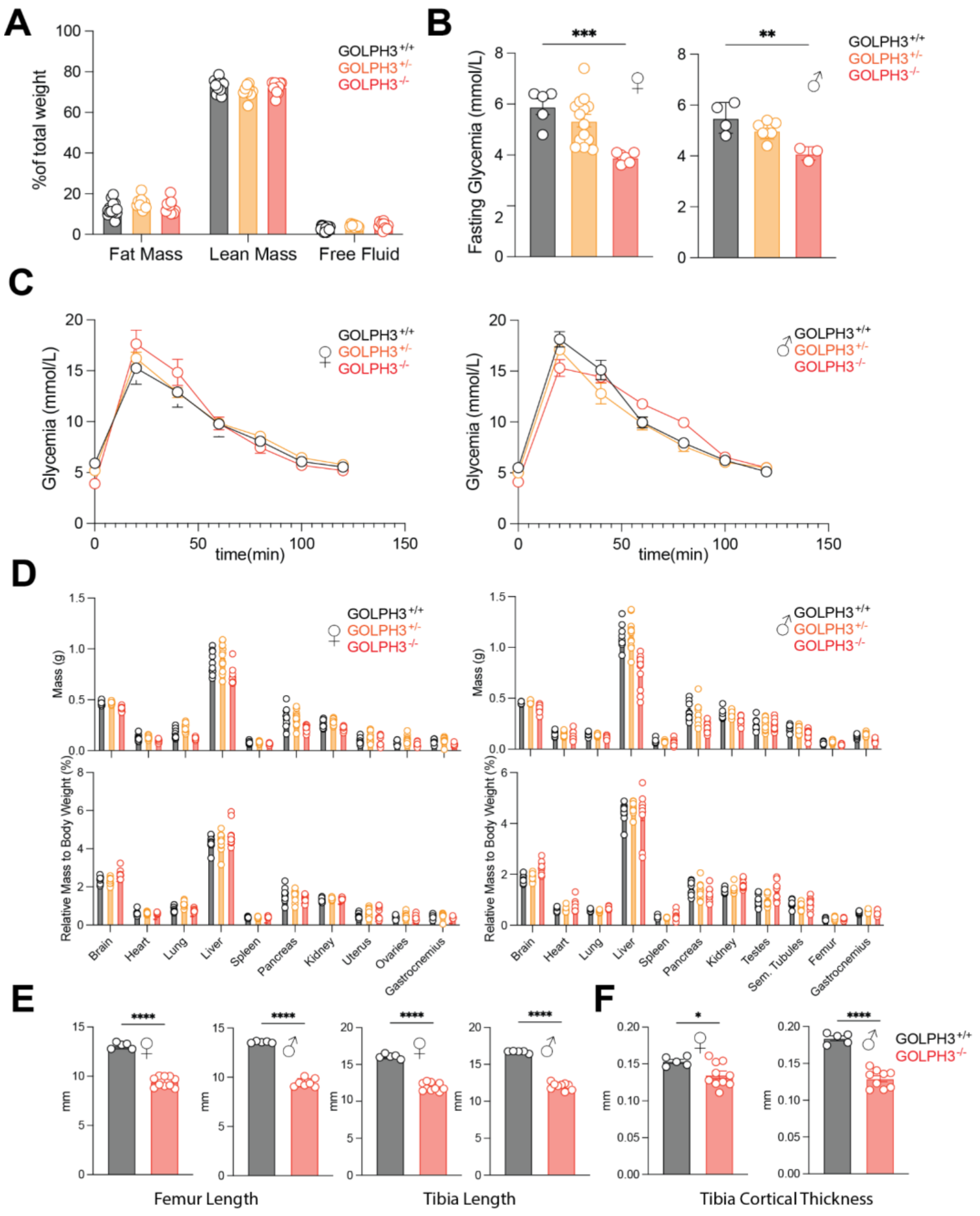

Figure S2: Metabolic and skeletal phenotyping of GOLPH3-deficient mice

A. Histogram showing body composition in 9-week-old GOLPH3$^{+/+}$, GOLPH3$^{+/-}$, and GOLPH3$^{-/-}$ mice, as determined by Echo-MRI. Data reflect the percentage of total body weight attributable to fat and lean mass. Values represent mean ± SEM.

B. Fasting blood glucose levels in 9-month-old GOLPH3$^{+/+}$, GOLPH3$^{+/-}$, and GOLPH3$^{-/-}$ mice. Data are presented as mean ± SEM (two-way ANOVA, **p < 0.001; ***p < 0.001).

C. Blood glucose concentrations measured over a 2-hour period following glucose administration in 9-month-old GOLPH3$^{+/+}$, GOLPH3$^{+/-}$, and GOLPH3$^{-/-}$ mice. Scatter plot shows mean ± SEM.

D. Histograms displaying the absolute (top) and relative (bottom) weights of internal organs in female (left) and male (right) GOLPH3$^{+/+}$, GOLPH3$^{+/-}$, and GOLPH3$^{-/-}$ mice. Data represent mean ± SEM.

E. Bone length measurements of the femur and tibia in 9-week-old male and female GOLPH3$^{+/+}$ and GOLPH3$^{-/-}$ mice. Data are shown as mean ± SEM (unpaired t-test, ****p<0.0001).

F. Tibial cortical thickness in 9-week-old male and female GOLPH3$^{+/+}$ and GOLPH3$^{-/-}$ mice, quantified by µCT analysis. Values represent mean ± SEM (unpaired t-test, *p < 0.05; ****p<0.0001).

Figure S3

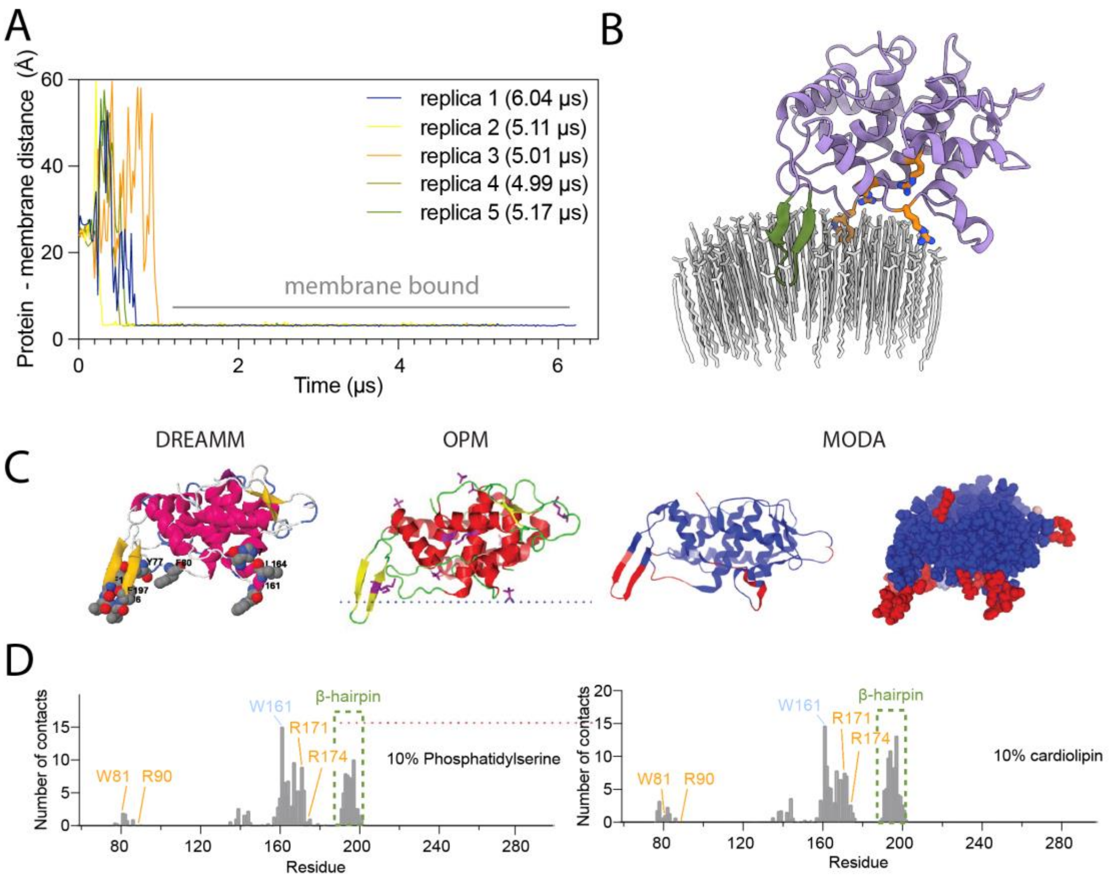

Figure S3. Predicted membrane orientation of GOLPH3

A. Minimum distance over time between any coarse-grained (CG) bead of GOLPH3 and the closest membrane bead across five independent CG-MD simulations with membranes containing 5% PtdIns(4)*P*. Five independent coarse-grained MD replicas are shown, simulation lengths for each replica are indicated.

B. GOLPH3 orientation relative to membrane mimics (oleic, palmitic, and myristic acids, grey sticks) predicted by AlphaFold3, using the maximum number of lipid molecules allowed.

C. Computational predictions of GOLPH3 membrane orientation. Left (DREAMM): cartoon of GOLPH3 with predicted membrane-interacting loops and side chains shown as space-fill; key hydrophobic/aromatic residues are labeled. Centre (OPM): protein orientation predicted by the OPM server (red cartoon) with the calculated membrane plane indicated by the blue dotted line. Right (MODA): backbone representation (blue) and corresponding solvent-accessible surface view; MODA-predicted membrane-contacting residues are highlighted in red. All panels are shown in the same overall orientation to facilitate comparison of predicted membrane-facing regions.

D. Average number of contacts per residue between GOLPH3 and membranes containing 10% phosphatidylserine (top) or 10% cardiolipin (bottom), based on CG MD trajectories.

Figure S4

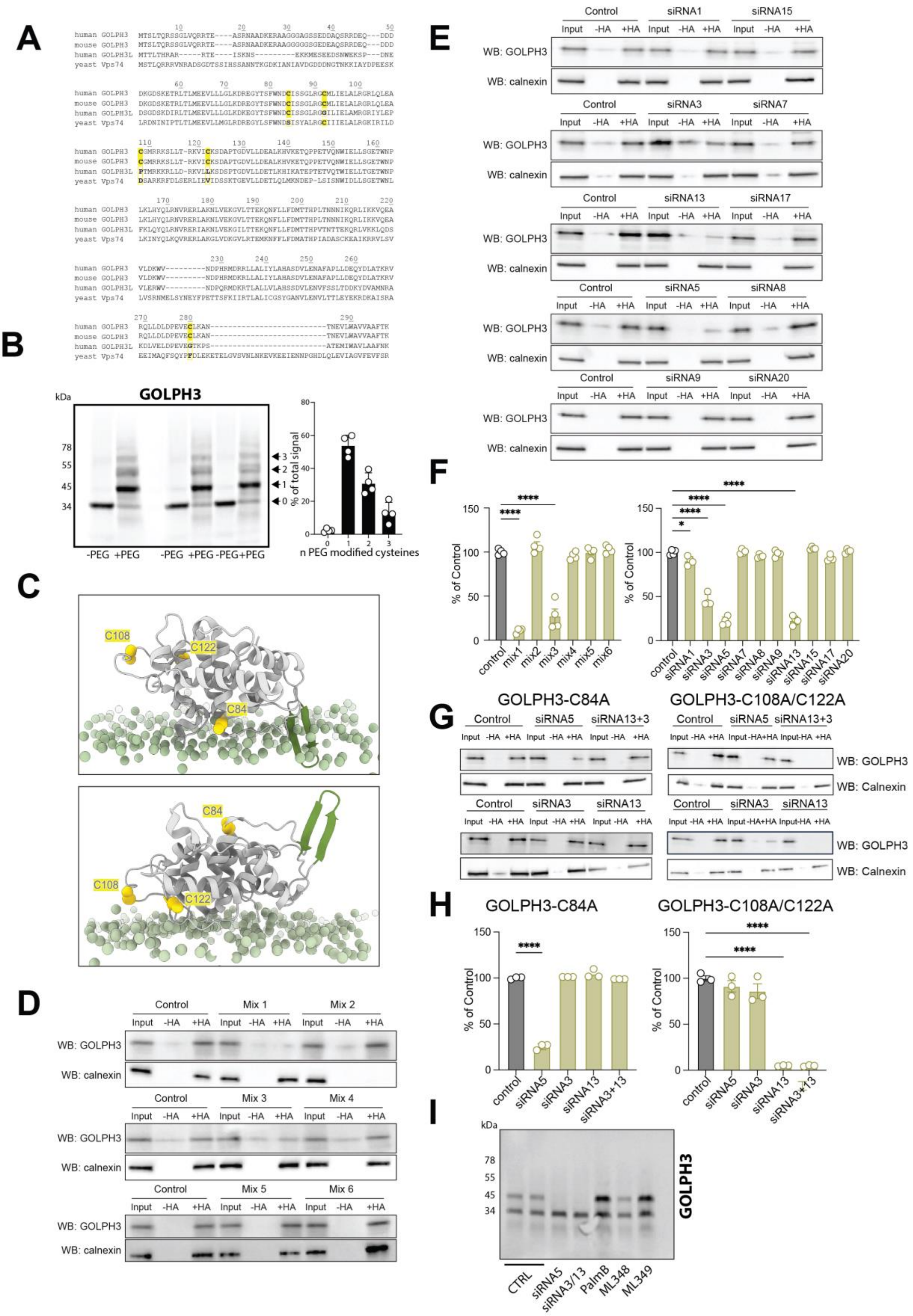
A
B
C
D
E
F
G
H
I
GOLPH3
GOLPH3-C84A
GOLPH3-C108A/C122A
WB: GOLPH3
WB: calnexin
n PEG modified cysteines
% of Control
% of total signal

A. Sequence alignment of human GOLPH3, mouse GOLPH3, human GOLPH3L, and yeast Vps74, highlighting the five cysteine residues in human GOLPH3 (yellow). Alignment was performed using Clustal Omega.

B. Stoichiometry of GOLPH3 palmitoylation determined in HeLa via PEG-5 labeling and anti-GOLPH3 western blotting (left) and its densitometry-based quantitation (right). Means ± SD, n=4.

C. Positions of the three palmitoylated cysteines on GOLPH3 relative to the membrane, shown in two MD-derived orientations: via the β-hairpin (top) and via the positively charged surface (bottom).

D–E. S-acylation of endogenous GOLPH3 in HeLa cells following siRNA knockdown of ZDHHC enzymes (C: siRNA mixes; D: individual siRNAs). Palmitoylation detected after hydroxylamine treatment (+HA); immunoblotting with anti-GOLPH3. Calnexin served as loading control.

F. Quantification of endogenous GOLPH3 S-acylation from (C–D). Mean ± SEM, n = 4. One-way ANOVA vs. control: : *p < 0.05; ****p < 0.0001.

G. S-acylation of GOLPH3-C84A and GOLPH3-C108A/C122A mutants in GOLPH3-KO HeLa cells treated with ZDHHC-targeting siRNAs.

H. Quantification of mutant GOLPH3 S-acylation from (F). Mean ± SEM, n = 3. One-way ANOVA vs. control: : ****p < 0.0001.

I. Dynamic S-acylation of endogenous GOLPH3 in HeLa cells assessed by click-PEG assay (see methods for details) under control conditions (CTRL), upon silencing of ZDHHC5 or ZDHHC3/13, and upon treatment with Palmostatin B (PalmB; 50µM), ML348 (10µM), or ML349 (10µM) for 4 hours.

Figure S5

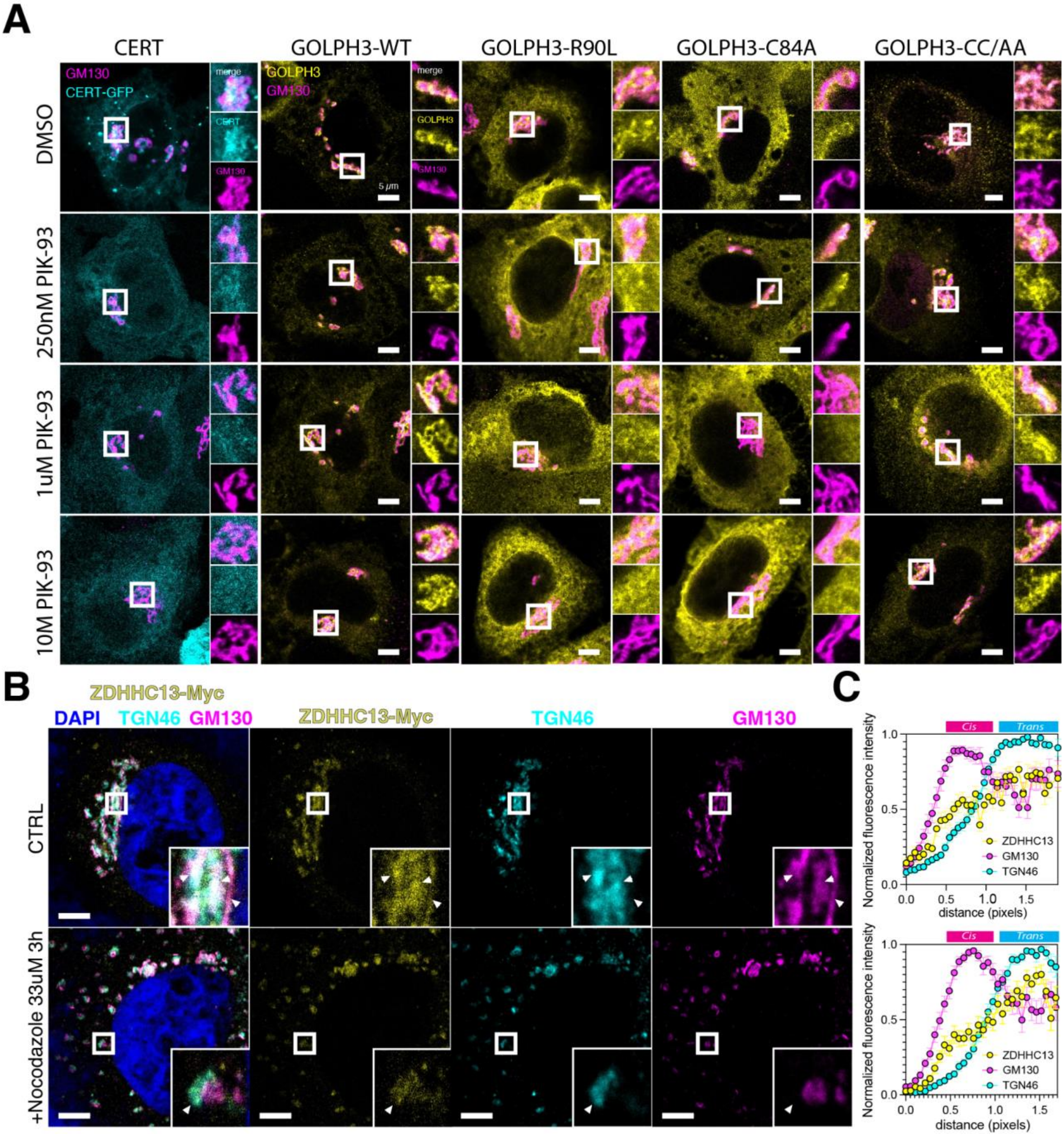

Figure S5. GOLPH3 regulation by S-acylation

A. Immunofluorescence analysis of GOLPH3 Golgi localization upon acute PtdIns(4)*P* depletion. GOLPH3-KO HeLa cells expressing CERT-GFP (cyan) or GOLPH3 variants (yellow) were treated with increasing concentrations of PIK93 for 1 h. Golgi localization, assessed by colocalization with GM130 (magenta), is highlighted in the insets. Scale bar, 5 µm.

B. Left: Super-resolution images of ZDHHC13-myc (yellow) in HeLa cells within the Golgi stack marked by the *cis*-Golgi marker GM130 (magenta) and the *trans*-Golgi marker TGN46 (cyan). Cells were either untreated (control) or treated for 3 h with nocodazole (33 µM) to induce the formation of ministacks, in

which *cis* and *trans* Golgi poles are better separated. Scale bar, 1 µm. Right: Line-scan quantification of ZDHHC13-myc intensity across the *cis*-to-*trans* Golgi axis in control and nocodazole-treated cells. Mean ± SEM, n = 35.

Figure S6

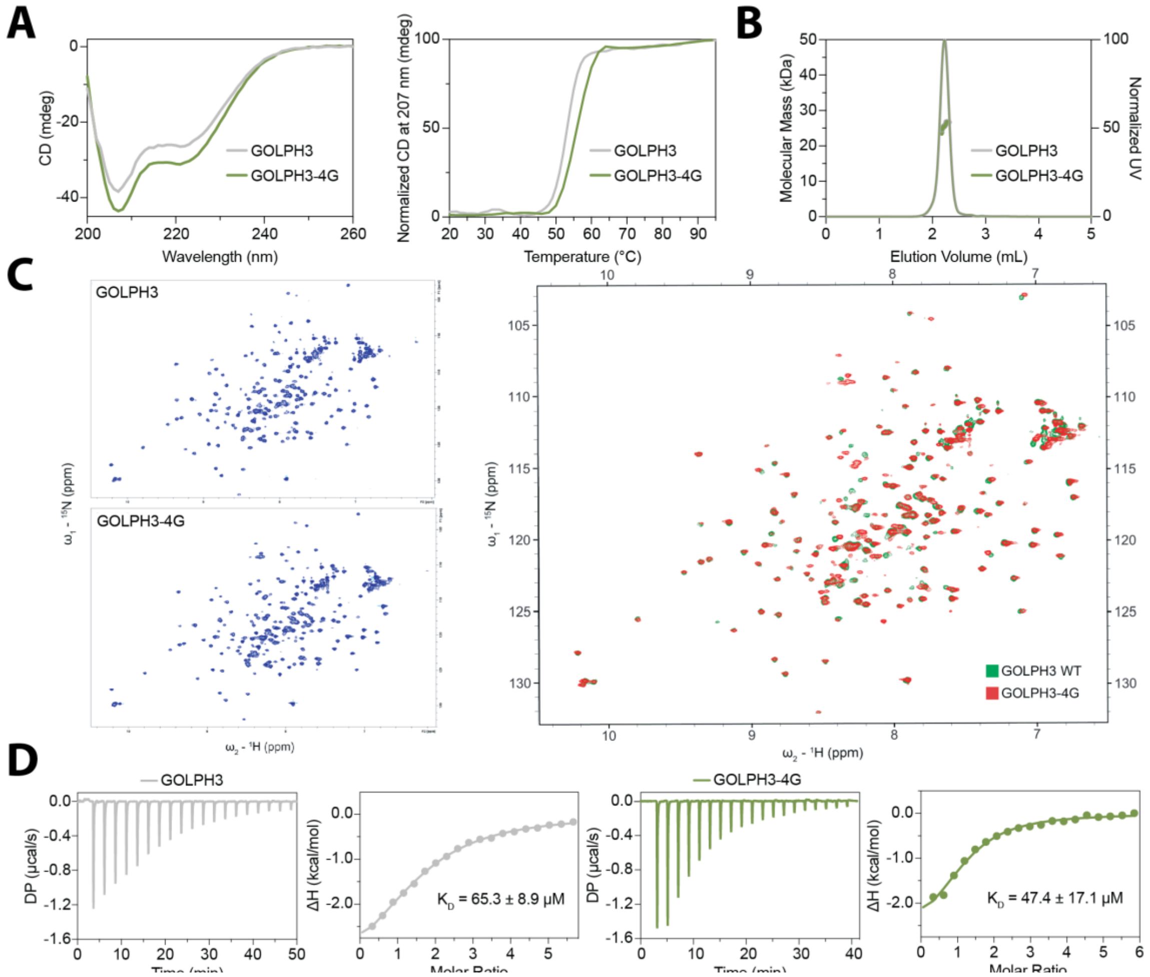

Figure S6. Validation of GOLPH3-4G as a surrogate for WT GOLPH3

A. CD spectra of GOLPH3-4G reveal secondary structure identical to WT. Right: normalized thermal denaturation monitored at 207 nm; Tm = 52.5 °C (WT) and 55 °C (4G).

B. SEC-MALS confirms that both GOLPH3 WT and 4G are monomeric.

C. Left: 2D ^{1}H-^{15}N HSQC spectra of ^{15}N-labeled GOLPH3 WT and ^{15}N-labeled GOLPH3-4G mutant. Right: overlay of the 2D ^{1}H-^{15}N HSQC spectra GOLPH3 WT (green) GOLPH3-4G mutant (red).

D. ITC measurements of LCS binding to GOLPH3 WT and 4G. Representative traces shown; K_D values are means from n = 3 technical replicates.

Figure S7

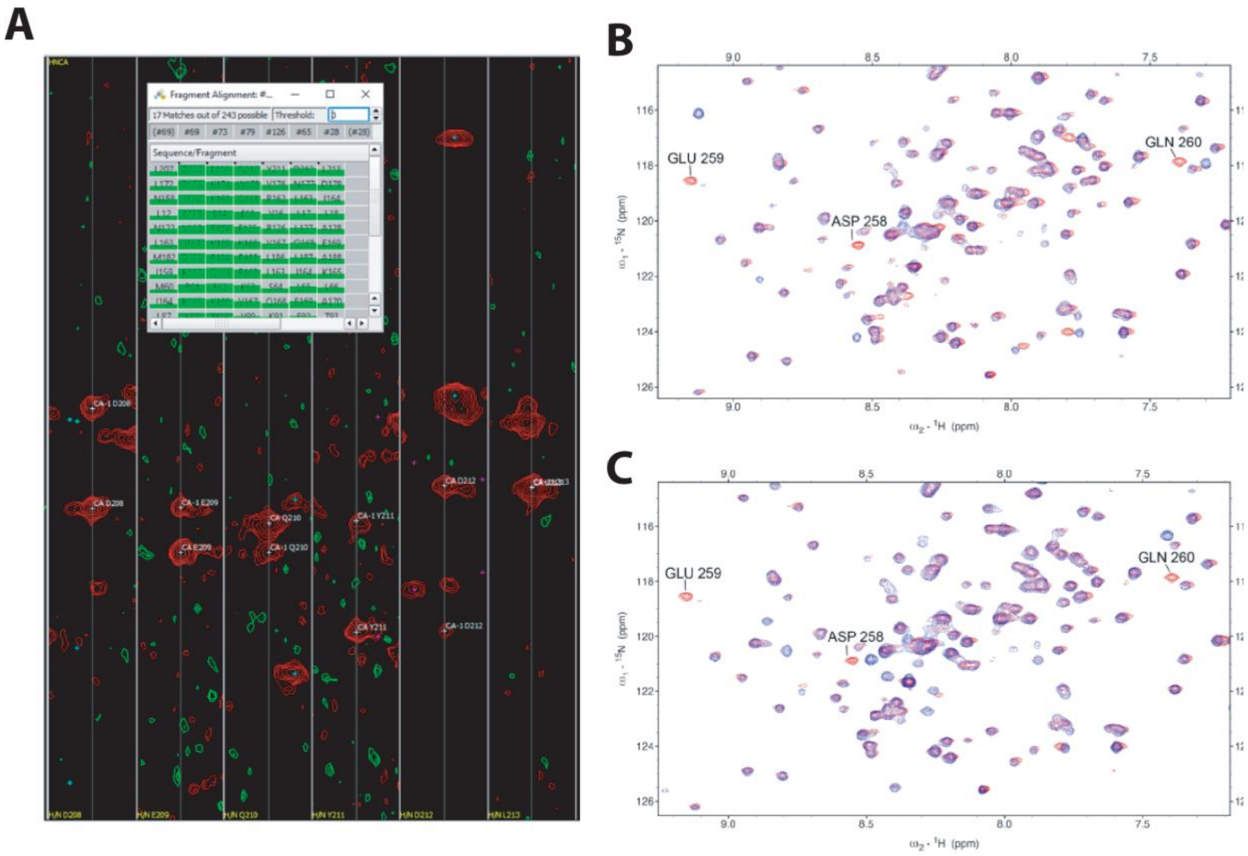

Figure S7. Confirming assignments for GOLPH3-4G's segment D258 to L263

A. HNCA strips showing a fragment of linked spin systems in the software CARA, best matching the sequence $D_{258}EQYDL_{263}$ (noting a -50 residue offset in the software), though chemical shifts could also fit other sequence segments.

B. HSQC spectra of GOLPH3-4G (red) and D258A (blue) mutants. Substantial shifts in D258, E259, and Q260 confirm the assignment.

C. HSQC spectra of GOLPH3-4G (red) and E259A (blue) mutants. Mutations cause clear shifts in E259, D258, and Q260.

Together, these confirm the assignment of the D258–L263 segment.

Figure S8

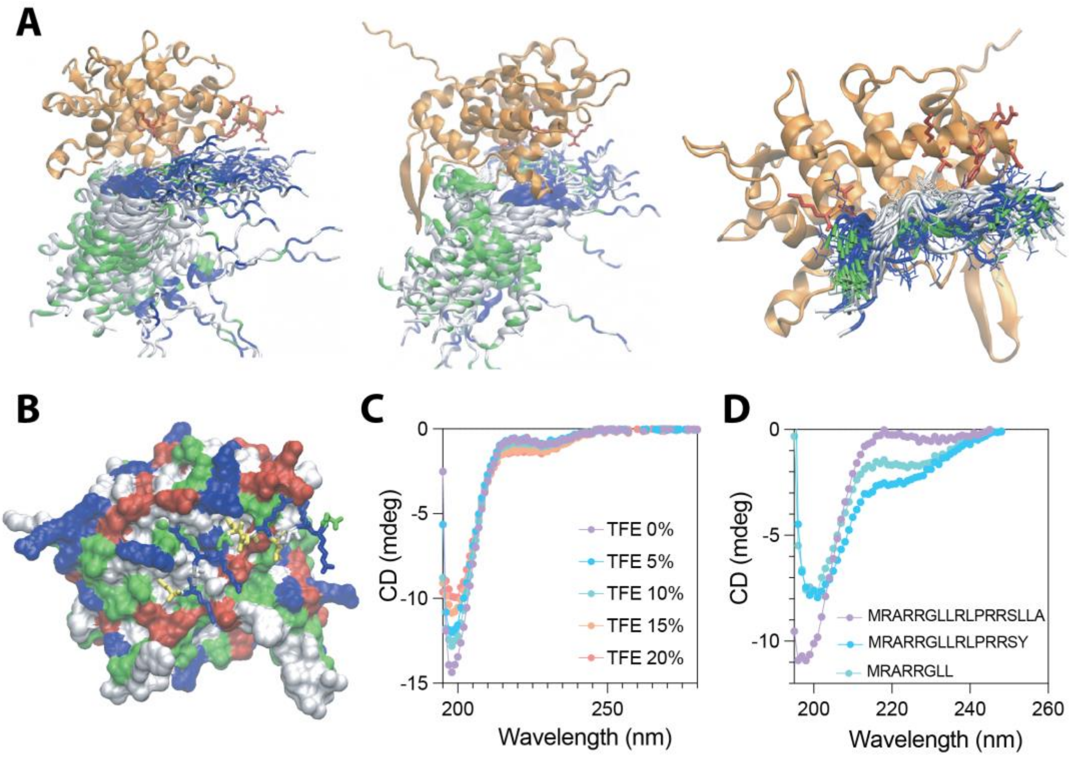

Figure S8. LCS tail binding to GOLPH3

A. Three views of models of GOLPH3 bound to the LCS peptide sampled from 1,000 AlphaFold3 runs with different random seeds (cartoon representation with sticks for positively and negatively charged interface residues).

B. Example pose in which GOLPH3 is shown as a surface to highlight insertion of hydrophobic residues from LCS (yellow) into hydrophobic clefts. Other colors follow standard VMD conventions: grey, hydrophobic; green, polar; blue, positively charged; red, negatively charged.

C. CD spectra of the LCS peptide in the presence of increasing concentrations of 2,2,2-trifluoroethanol (TFE).

D. CD spectra of the full-length LCS peptide (aa 1–17) and two truncated variants (aa 1–15 and aa 1–8).

Figure S9

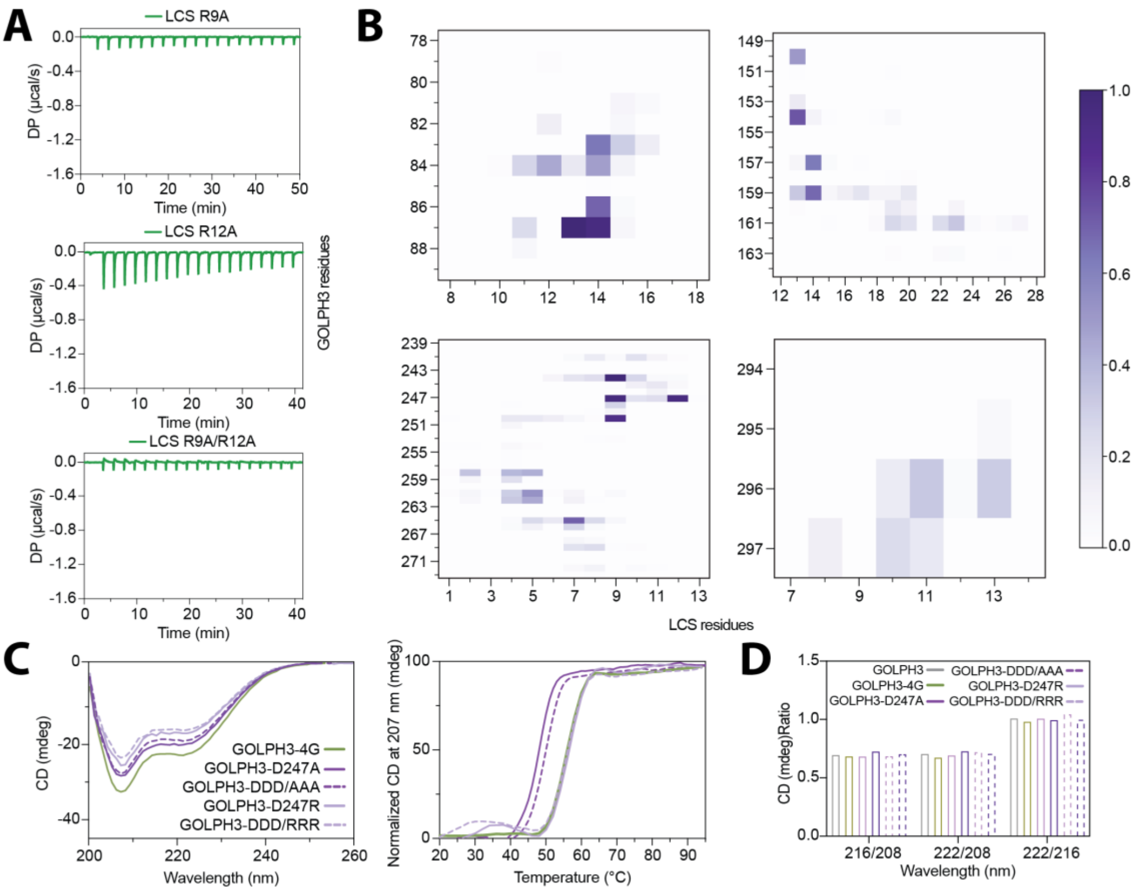

Figure S9. LCS-GOLPH3 binding interface

A. ITC traces showing binding of LCS mutants R9A (top), R12A (middle), and R9A/R12A (bottom) to GOLPH3 WT (n = 2 replicates).

B. Normalized heatmap showing residue-level contacts between S-acylated C84 GOLPH3 and LCS during MD simulations. Scale bar: contact intensity.

C. CD spectra of GOLPH3 mutants confirm WT-like secondary structure. Right: melting temperatures 4G (55 °C), D247A (48.5 °C), D247R (57 °C), D247A/D258A/D262A (49.5 °C), D247R/D258R/D262R (57 °C).

D. Ratios of ellipticity values of GOLPH3 mutant at 222, 216 and 208 nm.
