# Supplementary material for: Molecular regulation and physiological role of GOLPH3-mediated Golgi retention
Source: Nat Commun. 2026 Jun 11;17:7426. doi: 10.1038/s41467-026-74133-6 (PMC13408441; doi:10.1038/s41467-026-74133-6)
Supplement: Supplementary file 2 — Description of Addtional Supplementary Files [file 41467_2026_74133_MOESM2_ESM.pdf]

Supplementary Data File Description:

Supplementary Data File 1: Raw data from TMT proteomics experiments on liver samples from *GOLPH3* +/+ and *GOLPH3* -/- mice used to generate Figure 1C.

Supplementary Data File 2: Raw data from LWAC-TMT proteomics experiments on liver samples from *GOLPH3* +/+ and *GOLPH3* -/- mice used to generate Figure 1D.

Supplementary Data File 3: Raw data from LC-MS lipidomics experiments on liver samples from *GOLPH3* +/+ and *GOLPH3* -/- mice used to generate Figures 1G and H.

Supplementary Data File 4: Raw data from LC-MS/MS sphingolipidomics experiments on liver samples from *GOLPH3* +/+, *GOLPH3* +/-, and *GOLPH3* -/- mice used to generate Figures 1I and J.

Supplementary Data File 5: Raw data from LC-MS/MS sphingolipidomics experiments on *GOLPH3*-KO HeLa cells transfected with *GOLPH3* mutants used to generate Figure 5D.
