# Supplementary material for: Molecular regulation and physiological role of GOLPH3-mediated Golgi retention
Source: Nat Commun. 2026 Jun 11;17:7426. doi: 10.1038/s41467-026-74133-6 (PMC13408441; doi:10.1038/s41467-026-74133-6)
Supplement: Supplementary file 14 — Source Data5 [file 41467_2026_74133_MOESM14_ESM.pdf]

Figure 1B

WB golph3 and Tubulin

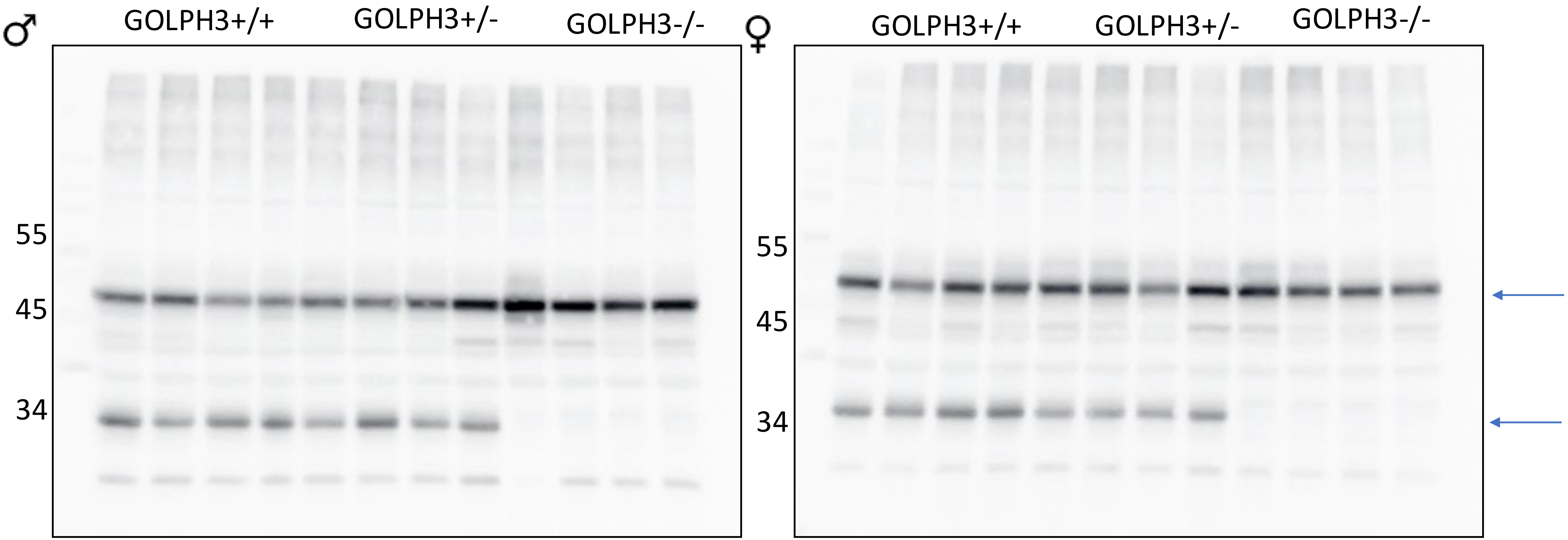

Figure S1A

Golph3

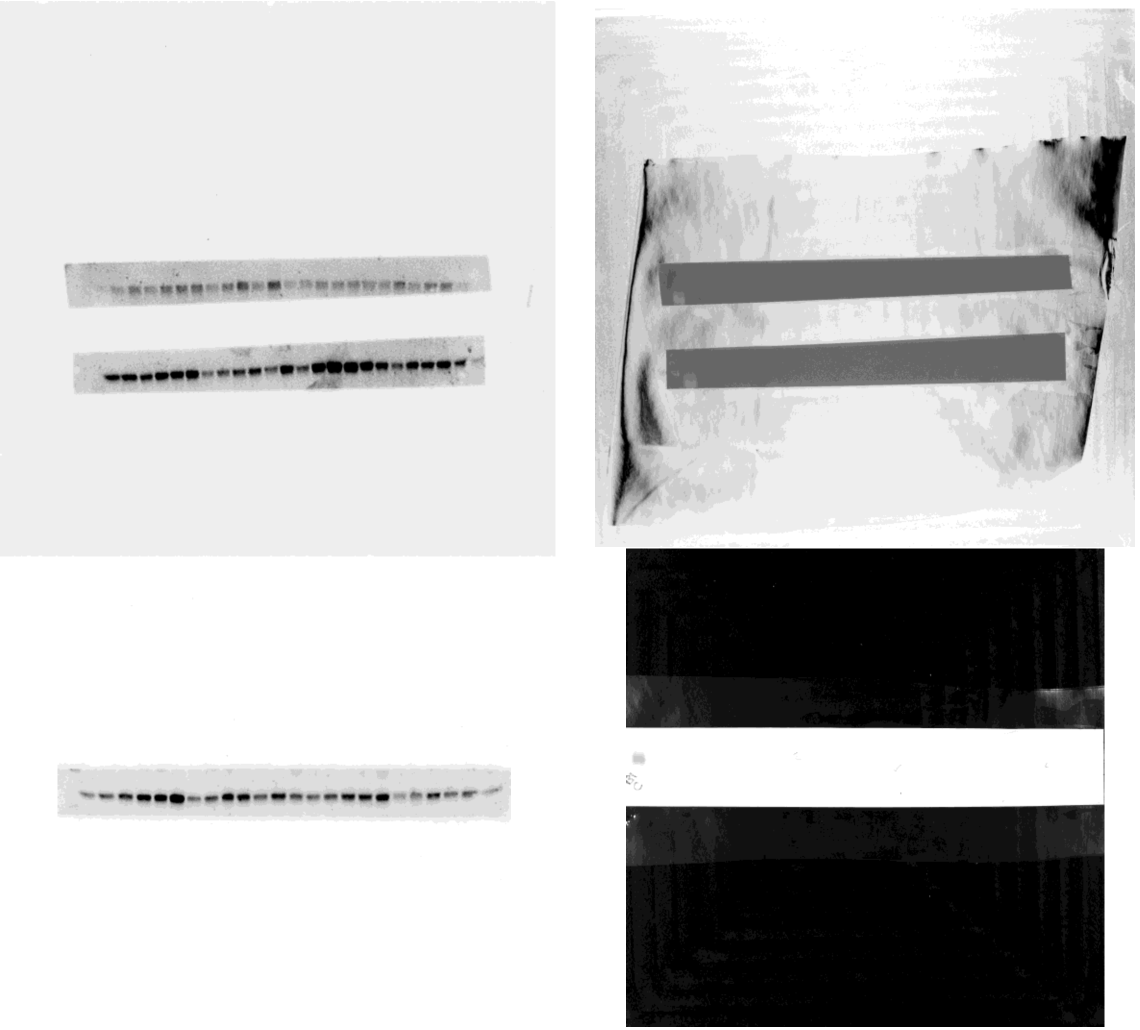

Tubulin

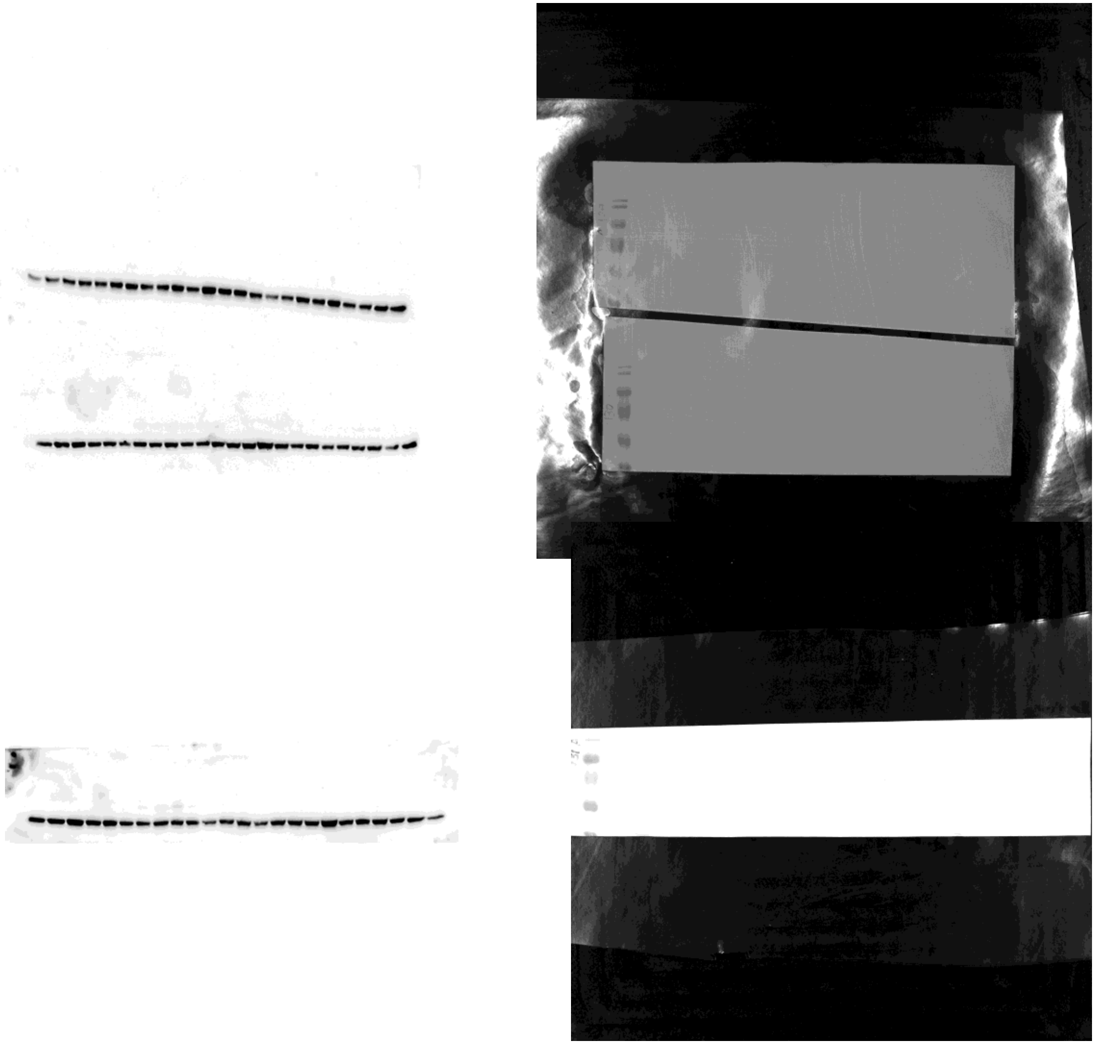

Figure 3E

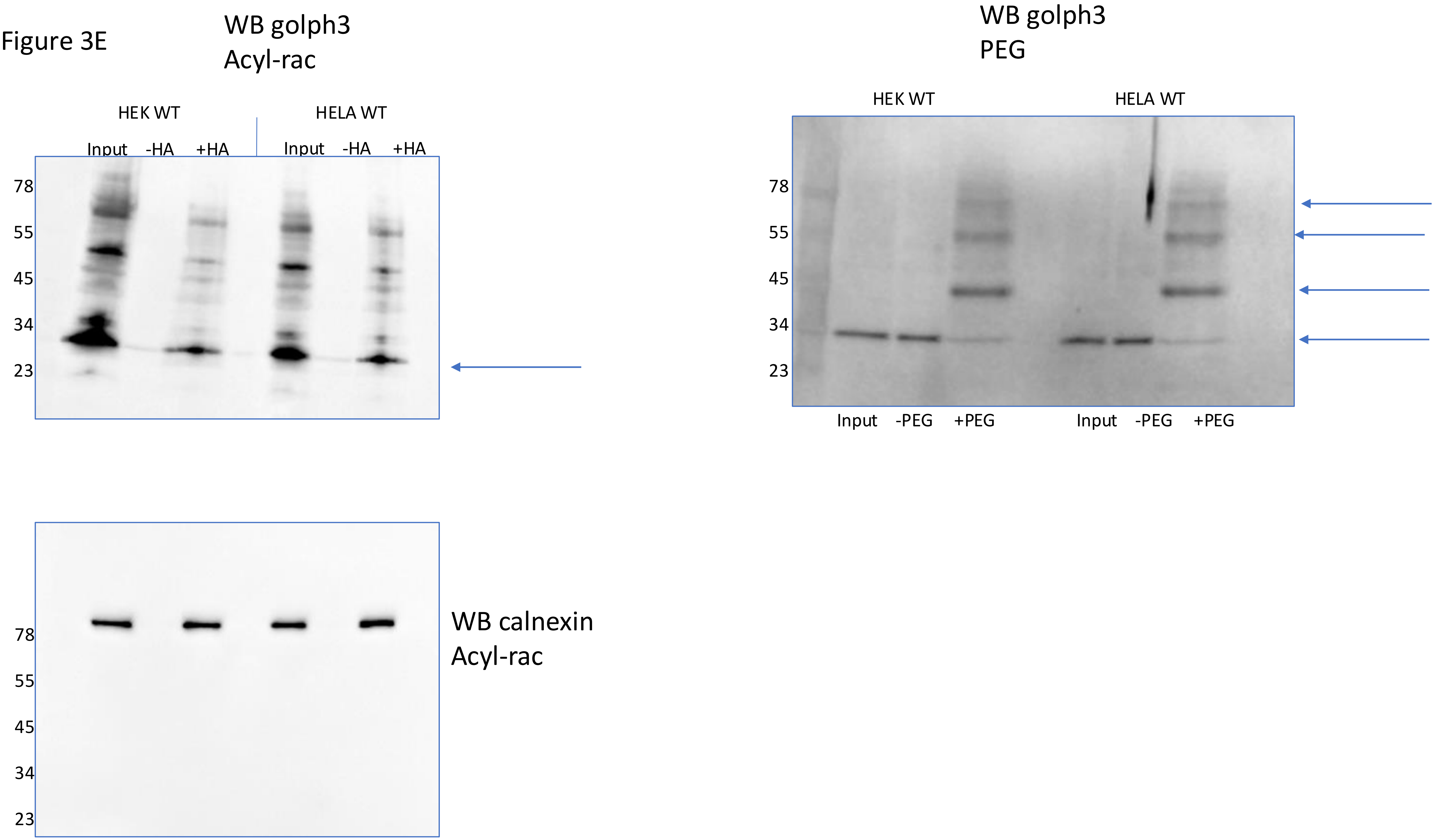
WB golph3
Acyl-rac
HEK WT
HELA WT
Input
-HA
+HA
Input
-HA
+HA
78
55
45
34
23
WB calnexin
Acyl-rac
78
55
45
34
23
WB golph3
PEG
HEK WT
HELA WT
78
55
45
34
23
Input
-PEG
+PEG
Input
-PEG
+PEG

Figure S4B

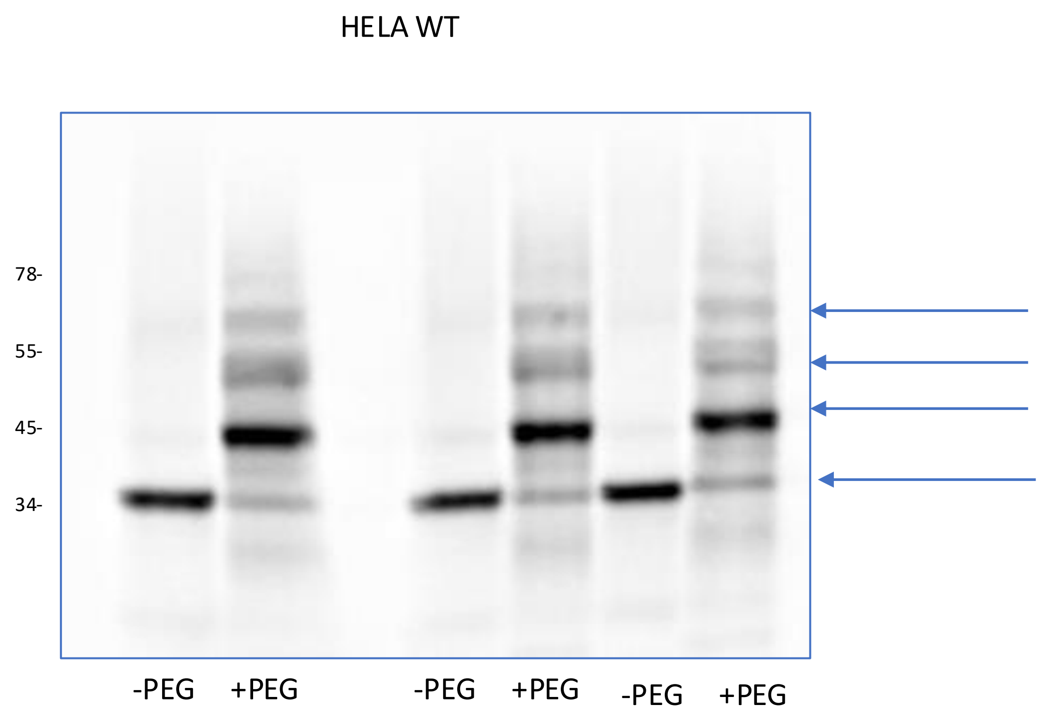

WB Golph3 endogenous

Figure S4D

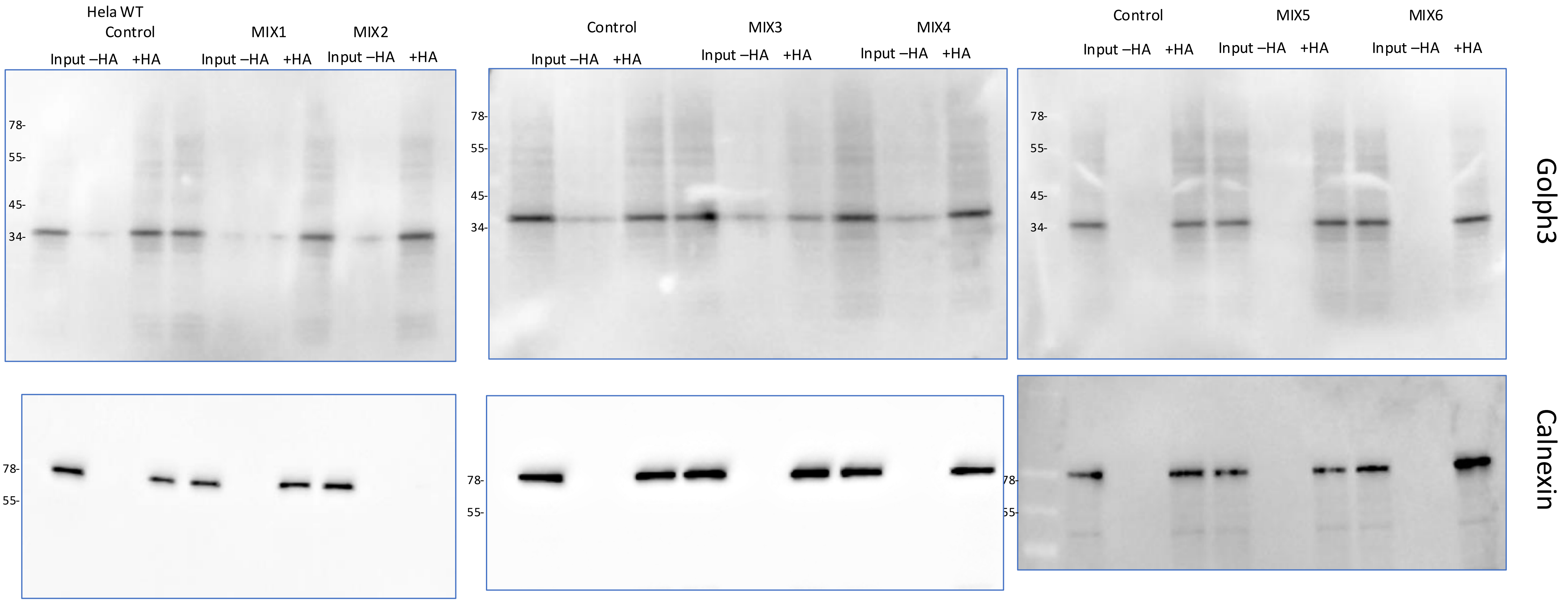

Hela WT
Control
MIX1
MIX2
Input –HA +HA
Control
MIX3
MIX4
Control
MIX5
MIX6
78-
55-
45-
34-
Golph3
Calnexin

Figure S4E

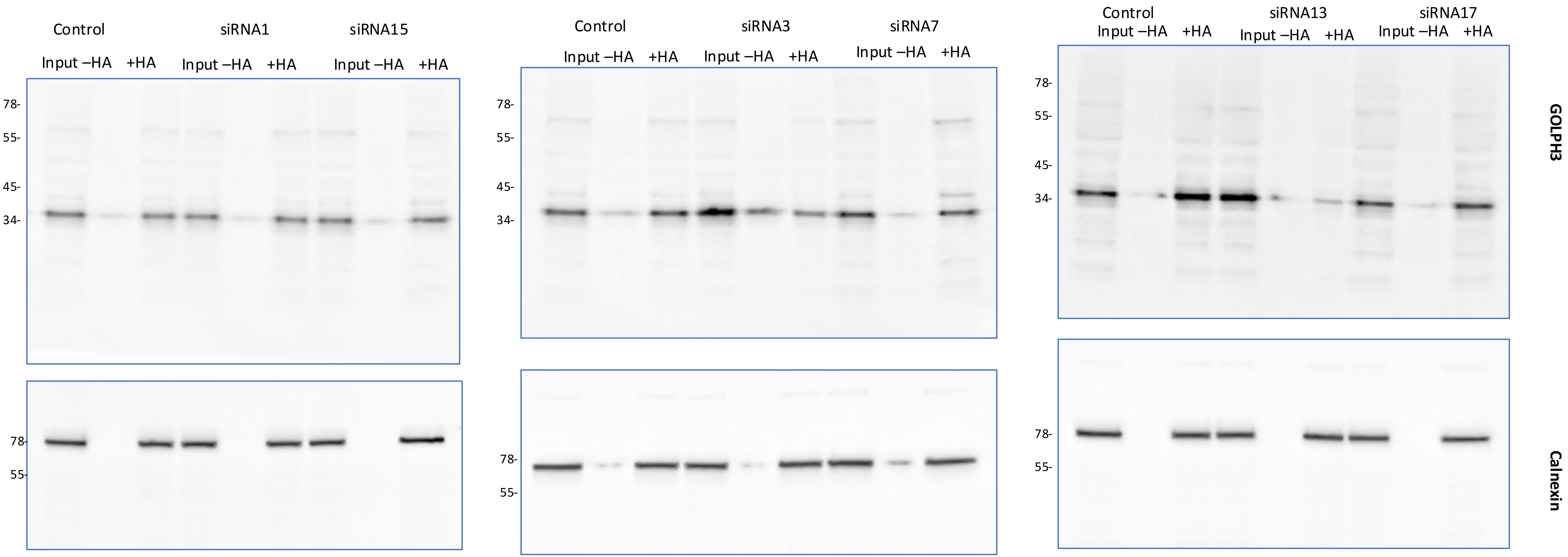
Control
siRNA1
siRNA15
Input –HA +HA
Input –HA +HA
Input –HA +HA
78-
55-
45-
34-
Control
siRNA3
siRNA7
Input –HA +HA
Input –HA +HA
Input –HA +HA
Control
siRNA13
siRNA17
Input –HA +HA
Input –HA +HA
Input –HA +HA
GOLPH3
Calnexin

Figure S4E

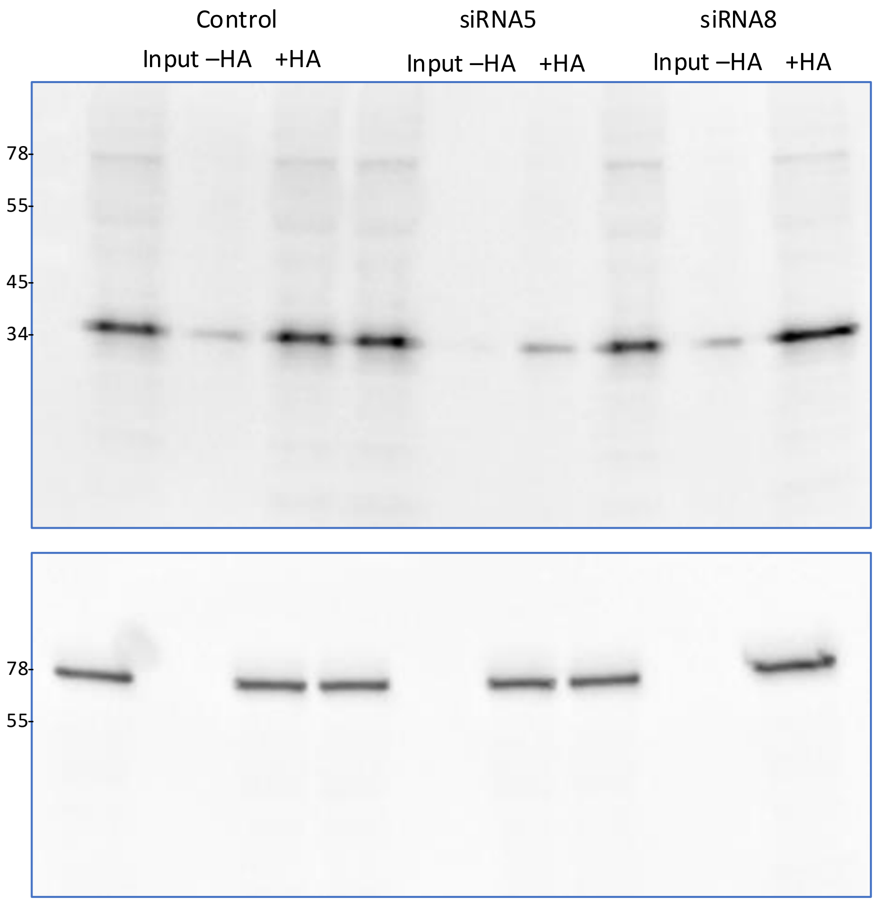

Control
Input –HA +HA
siRNA5
Input –HA +HA
siRNA8
Input –HA +HA
78-
55-
45-
34-
78-
55-

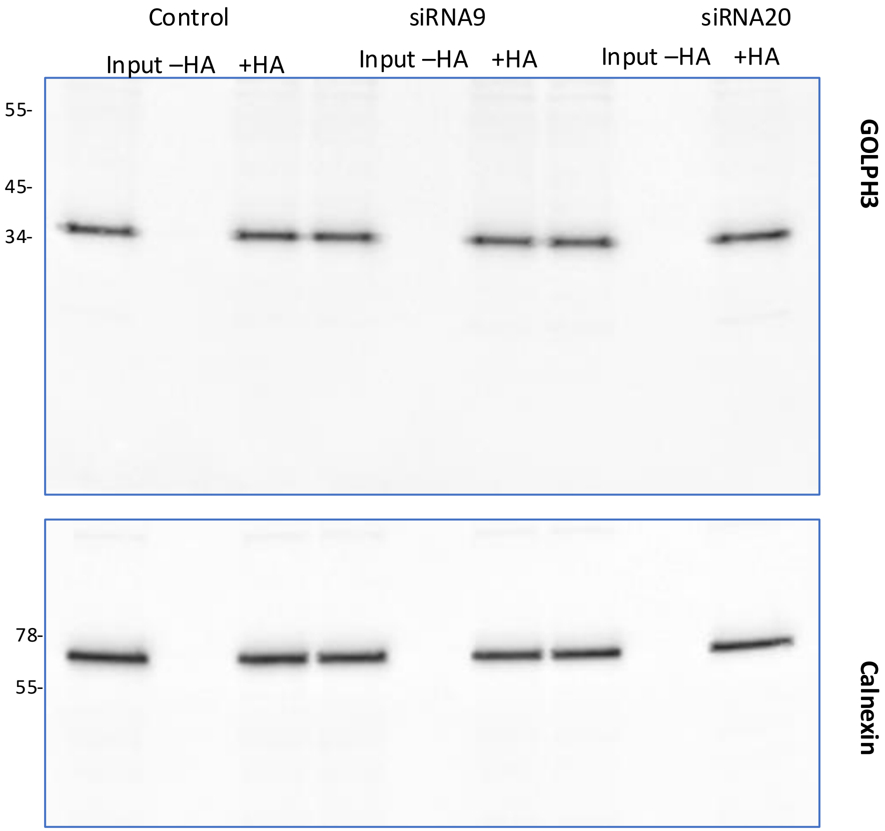

Control
Input –HA +HA
siRNA9
Input –HA +HA
siRNA20
Input –HA +HA
55-
45-
34-
78-
55-
GOLPH3
Calnexin

Figure S4G **Cys84A**

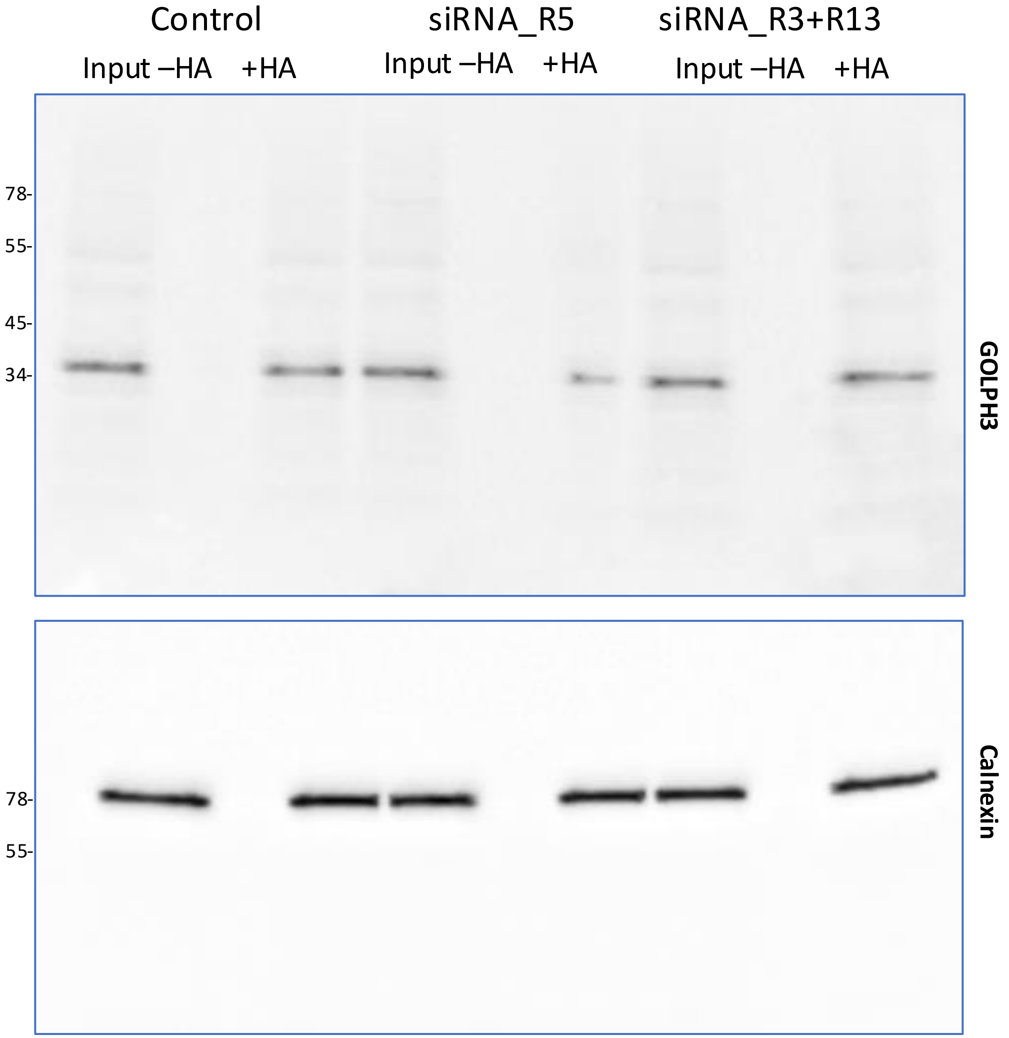

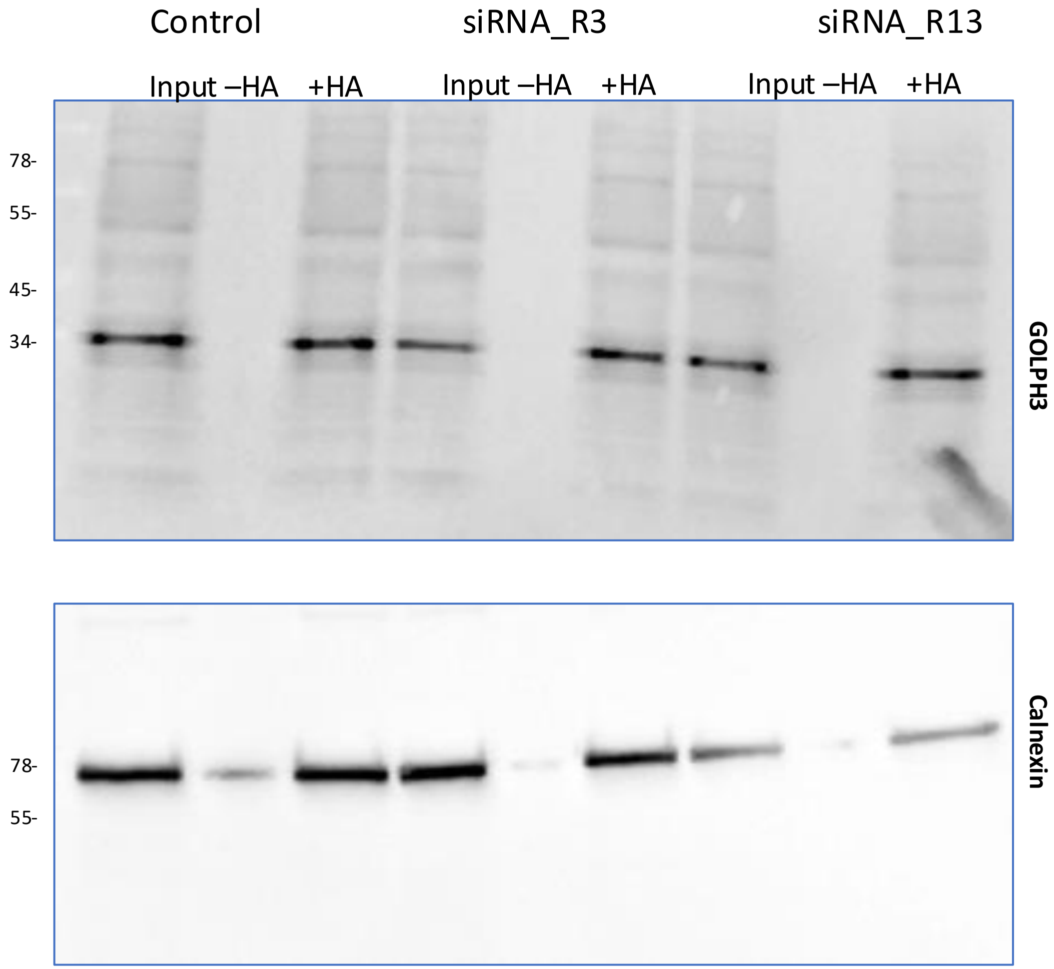

Figure S4G **CYS108A-CYS121A**

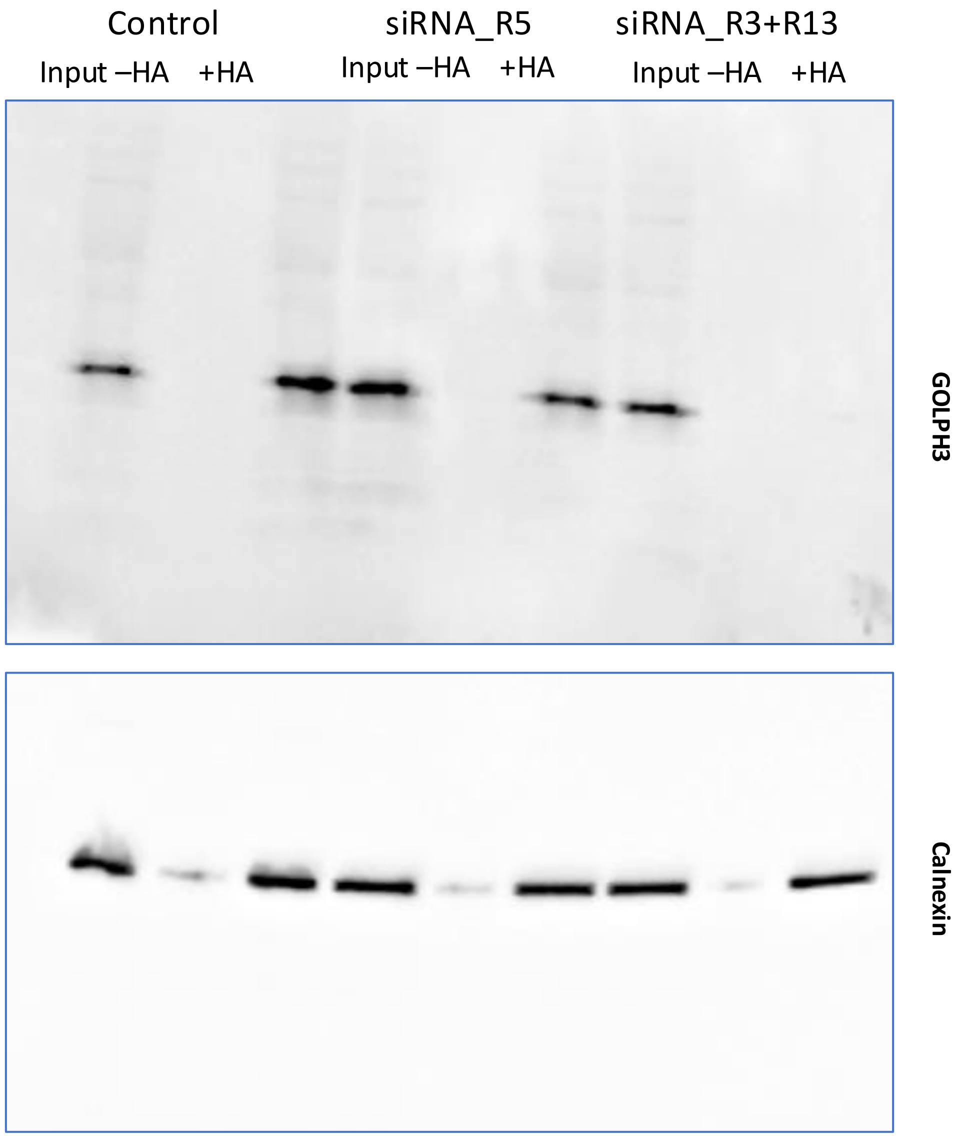

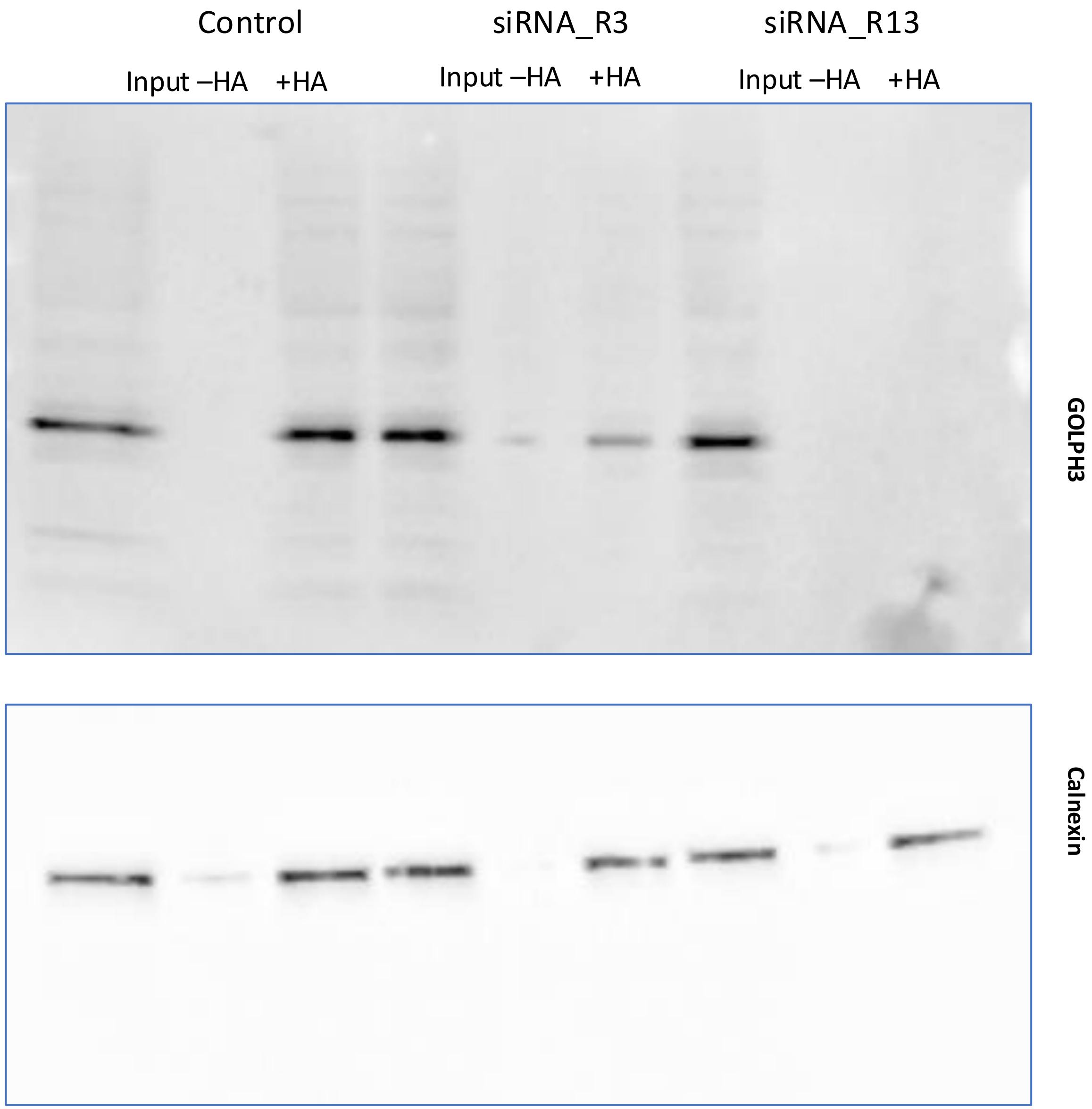

Figure S4I

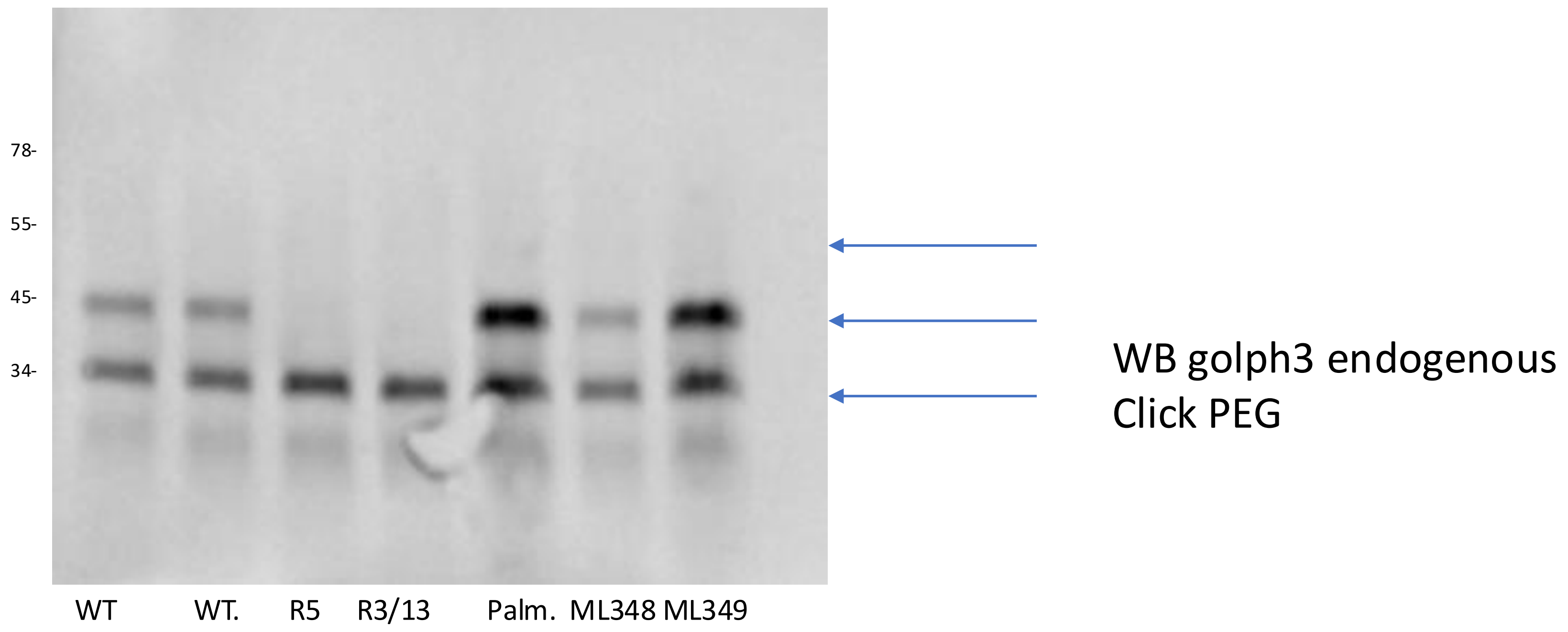

78-
55-
45-
34-
WT
WT.
R5
R3/13
Palm.
ML348
ML349
WB golph3 endogenous
Click PEG
