# Supplementary material for: Molecular regulation and physiological role of GOLPH3-mediated Golgi retention
Source: Nat Commun. 2026 Jun 11;17:7426. doi: 10.1038/s41467-026-74133-6 (PMC13408441; doi:10.1038/s41467-026-74133-6)
Supplement: Supplementary file 9 — Transparent Peer Review file [file 41467_2026_74133_MOESM9_ESM.pdf]

nature portfolio

Peer Review File

Molecular Regulation and Physiological Role of GOLPH3-mediated Golgi retention

Corresponding Author: Professor Matteo Dal Peraro

This file contains all reviewer reports in order by version, followed by all author rebuttals in order by version.

Version 0:

Reviewer comments:

Reviewer #1

(Remarks to the Author)
This manuscript addresses the physiological role and molecular regulation of GOLPH3, a Golgi adaptor implicated in retrograde trafficking of glycosyltransferases. Using a combination of mouse knockout models, glycoproteomics, lipidomics, structural biology (NMR, MD simulations), and cell-based assays, the authors reveal a dual S-acylation–mediated regulatory mechanism that governs GOLPH3's orientation and client-binding capacity. The study connects these mechanistic insights with in vivo effects such as glycosylation defects, embryonic lethality, etc. Overall, the work is well executed, using a multidisciplinary approach and is highly relevant to the niche fields of glycosylation and membrane trafficking. The study would be a useful addition in the literature provided that the following revisions are performed:

Major Revisions

1. Mutants (C84A, C108A/C122A, etc.) are used in cell lines. It would strengthen the manuscript to demonstrate partial or full rescue of glycosylation/phenotypic defects in knockout mice or primary cells. This would establish direct physiological relevance of the regulatory mechanism.

2. The different orientations depending on PIP4 lipid composition are interesting. How do these orientations compare with available web-servers that predict the GOLPH3 membrane orientation such as DREAMM, OPM, MODA, and PMIpred? The authors should present this comparison and include it in Figure S3.

3. The membrane composition states composition percentages, but it does not specify whether they are %mol, %v/v, %w/w. Please clarify.

4. What is the duration of each of the five independent replicas for each of the CG MD systems? It is not mentioned explicitly in the manuscript, but one can tell from Figure S3A that they have varying duration.

5. The manuscripts overall lacks report and discussion of the statistical variance, especially in the calculations. For example, all the CG results should report standard deviations from the 5 replicas (for example, standard deviations should be added in the following sentence: "GOLPH3 embedded ~5 Å deeper into the membrane and made ~20 546 % more contacts"), but also in any other places where quantitative results are presented. Moreover, more detail on the statistical methods used for lipidomics/proteomics analyses (e.g., FDR correction, replicate number) would enhance transparency.

6. The connection between glycosylation defects and bone phenotypes could be elaborated, potentially referencing known glycosylation disorders with skeletal involvement. Moreover, a short discussion/comment could be added in the context of GOLPH3 being frequently overexpressed in cancer. The Discussion section could connect the regulatory model presented here with potential mechanisms underlying oncogenesis or altered glycomes in tumors.

7. Terms such as "inactive state" and "alternative orientation" could be better defined for clarity.

Reviewer #2

(Remarks to the Author)
The mechanism by which glycosylation enzymes are retained in distinct subcompartments of the Golgi is a longstanding question in cell biology. In this study, the authors generated GOLPH3 KO mice and report that GOLPH3 depletion disrupts both protein and lipid glycosylation, causes partially penetrant embryonic lethality, and severely impairs growth and bone mineralization. Using biophysical and biochemical approaches, they further show that GOLPH3 topology and membrane association are regulated by palmitoylation and PI4P levels, and that GOLPH3 binds the cytosolic tail of LCS (B4GALT5), which is required for its Golgi retention.

Overall, the study is of high quality and significance, and the manuscript is clearly written.

However, the evidence remains relatively limited to support the strong conclusion that GOLPH3 is a central regulator of Golgi enzyme retention.

Major concerns:
1. The GOLPH3 knockout in mice appears incomplete. The manuscript does not explain what residual protein or fragments remain and how these might influence the phenotypes described. Relatedly, in Figure 3E, a band is still visible in the [^{3}H]-palmitate autoradiograph in the GOLPH3 KO lane, similar to that observed in the GOLPH3-5CA lane. The authors should clarify the identity of this band.

2. The evidence for GOLPH3's role in retaining Golgi enzymes is limited to LCS. Do other glycosyltransferases interact with GOLPH3, and is the interaction regulated by PI4P levels? Currently, the lack of broader analysis (e.g., co-IP or pulldown assays) weakens the general conclusion that GOLPH3 mediates retrograde trafficking of multiple Golgi-resident enzymes. In addition, could cell-surface biotinylation/streptavidin pulldown followed by MS analysis reveal which Golgi enzymes are mislocalized in GOLPH3 KO cells compared with WT cells?

3. In the MD simulations, the authors conclude that PI4P is not strictly required for GOLPH3 membrane association, but modulates binding orientation in a concentration-dependent manner. Is there an experimental way to validate this prediction? For instance, what are the actual PI4P levels in the TGN in the relevant conditions? Where is ZDHHC13 localized within the Golgi, and how does palmitoylation interplay with PI4P levels to regulate GOLPH3 membrane association and enzyme interaction?

4. The link between LCS mislocalization and the developmental/growth defects observed in mice is insufficiently addressed. In addition to confirming that LCS is mislocalized in GOLPH3 KO mice, the authors should also provide a more direct explanation or mechanistic model for how loss of LCS retention leads to the complex in vivo phenotypes.

Reviewer #3

(Remarks to the Author)
Summary Comments on Paper:
The purpose of this paper is to understand the biological role of GOLPH3 in intra-Golgi trafficking. To achieve these aims, the manuscript used a variety of approaches, ranging from gene deletion of GOLPH3 in mice models, to detailed molecular delineation of the possible mechanisms of GOLPH3 function marrying computer simulations with detailed designed of experimental proofs of the results from these simulations; this combination of computer-aided studies with experimental proofs makes this paper interesting and is a good example of how these two approaches could be used in a complementary manner to make new discoveries. A very considerable amount of work is included in this paper with many groups involved. Much of the work were done in sufficient detail and with logical approaches to arrive at an interesting story.
The manuscript contributes well to increasing our knowledge of the importance of GOLPH3 in intra-Golgi trafficking through its abilities to simultaneously bind to Golgi membrane and cargo proteins in a structurally viable manner.

Major General Comments
The manuscript requires revision to improve its clarity, especially in the discussion section; obviously there had been significant amount of work involving many groups from different disciplines. The discussion section does not do justice to the efforts expended in the arriving at the conclusions. There should be more links between the results obtained and how this work has advanced our understanding of biological function of GOLPH3, rather than making general statements most of which are not linked to the current studies. Two examples are:
Line 709: ". These modifications toggle GOLPH3 between an active, Golgi-localized, client-binding–competent state and an inactive, plasma membrane–bound state with inverted topology"- What makes the GOLPH3 "active" or "inactive"; why does binding to the Golgi make the protein active whereas binding to the plasma membrane make the protein inactive. Which specific results demonstrate this?
Line 717: "Our findings reveal a new layer of control in glycosylation" - How did the results show this? Need to refer back to the specific findings.

More specific Comments on Specific Sections:

NMR Comments
1. Are there enough peaks in the 2D HSQC spectra to match the number of residues of GOLPH3? Please include number of resolved backbone amide peaks.
2. The NMR peaks do not seem to have similar intensities, especially signals from the folded region of the spectrum (most

obvious in Figures S6 and S7 where there are fewer overlays of spectra making it more obvious that the peaks are not of similar intensities). These resonance intensities vary by considerable amounts to cause concern. This is particularly curious given that GOLPH3 does not have an unusual shape for its structured core that might give rise to unequal signal intensities. A contributory factors could be that under NMR conditions and concentrations (unlike verification using SECMALS conditions) some degree of aggregation or multimerization or even disulphide bond formation had occurred. Can the authors comment on this resonance variation and include a spectrum without overlays of with GOLPH3-WT or GOLPH3-4G in the supplementary.
3. A quick T2 experiment could be carried out to verify that the NMR protein sample concurs with a monomeric form.
4.Using point mutants to confirm the assignments of a protein is a standard approach. However, the concern here is that mutation from D/E to A are nonconservative mutations carried out on residues that occur within structured helical regions of GOLPH3. There is a risk that structural changes could have occurred which alter the shifts of many other resonances (Lines 631 and 633, Fig S7B and C) and influence the robustness of using these point mutants to confirm the triple resonance assignments. Quantification of the number of peak perturbations in the different mutants should be presented in the supplementary.
5. Interactions with LCS peptide:
(a) Summarise all the chemical shift perturbations with magnitude of changes.
(b) AlphaFold Multimer Model: Only one structure was presented as the only result but is this so? There are insufficient details given about the output statistics, how many poses were obtained, did they converged, how was the best pose selected, etc. This is a new generation of AI tool. Were others generative AI tools attempted? What model system was tried to verify the AF-Multimer approach?
(c) The structures shown in Figure 4C should be deposited in a public database such as Modelarchive.
6. Mutations of GOLPH3 for invitro Assays:
(a) it is not clear for the NMR, ITC, CD and interactions with LCS peptides whether site-directed mutants were performed on GOLPH3 or GOLPH3-4G. Please clarify – this is particularly important for understanding the effects of mutations on the melting temperatures.
(b) the mutations on the protein did produce measurable effects on the secondary structures of GOLPH3. As many of the point mutants were carried out on residues located in stable secondary structures, as well as the mutations were not conservative ones, some perturbations of protein secondary structures are expected and from the ellipticity calculations it is possible to quantify the extent of these perturbations, which in turn could affect the interpretation of some of the data; this should be done.
(c) Have point mutation effects on the secondary structures of LCS peptide been assessed either computationally or experimentally?

Other more specific comments:
Line 366 and Figure 3: The data and description does not quantitatively address S-palmitoylation in the cells. All these data are showing is the presence of S-palmitoylation rather than the actual level of this post-translational modification. Secondly, this PTM is dynamic since S-palmitoylation is reversible. Hence, these data and the description of the results is a snapshot of the level of PTM which could be quite small; this cannot be assessed from the current data since they are normalised to the WT level rather than an independent measure with, for example a quantifiable standard. Hence, the important and significance of the S-palmitoylation as an added mechanism in its role for GOLPH3 intra-Golgi trafficking need to be tempered.
Line 366 and Figure 3E : What is the difference between lanes 1 and 3- they seem to be both from WT HeLa cells. Please clarify in figure legend.
Line 429: A rationale should also be given as to why the 5 cysteine residues are not S-palmitoylated to the same degree. Is this a structural or sequence or both issue?
Line 433-There is a need to have more details about the structure of GOLPH3 based on PDB 3KN1; although the crystal coordinates were released in 2009, a detailed description of the structure has not been published. At the very least some calculations of the electrostatic surface of GOLPH3 based in PDB 3KN1 should be presented both in the text and in the figures where references are made to the positive and negative surfaces of the protein. FigS5 Panel D should feature more prominently in the main figures rather than tucked away in FigureS5 since the positive and negative surfaces are important for the function of the protein and discussed much earlier in the text.
Line506: State the % sequence specific assignments achieved.
Line 427: Explain the functional significant of C108 and C122 being less S-acylated than C84.
Line 441: Explain the significance/rationale of ZDHHC13 specificity for C84 and ZDHHC5 for C108 and C122.

Reviewer: Lu-Yun Lian

Reviewer #4

(Remarks to the Author)
The study explores molecular and cellular changes accompanying body-wide deletion of functional GOLPH3 in mice. Supported by various -omics analyses, an aspect this reviewer specifically was encouraged to look at, one conclusion is that disruption of GOLPH3 induces “glycosylation defects” both in terms of mucin-type O-glycosylation and GSL glycosylation. While such associations indeed appear to be supported, the manuscript does not well enough point out that evidence for a direct causal relationship between GOLPH3 dysfunction and altered glycosylation has not yet been provided. Conceivably, in addition to the involvement in retention and trafficking of GTs in the Golgi, GOLPH3 disruption may have unknown indirect functions that can’t be disregarded such as for example by impacting the expression of nucleotide sugar transporters or glycosyltransferases/hydrolases or altering the glycosylation machinery in other unexpected ways through indirect

pathways. As the glycoenzymes are often low abundant most of these would typically not be observed by proteomics and therefore require transcript level assessment. Further, the statement that “loss of GOLPH3 disrupts glycosylation” is not accurate as the authors only presented data for the reduction of one or two form(s) of mucin-type glycosylation (T and/or Tn antigen) in GOLPH KOs, and thereby neither looked at if other glycoforms or the non-glycosylated forms were concomitantly increased nor identified which specific GTs were impacted by GOLPH ablation. Without such evidence, the conclusions should be better presented as “GOLPH3 loss appears to be associated with a global change in the protein and lipid glycosylation machinery albeit the specific glyco-enzymes driving these changes are yet to be established”. Finally, “Our findings reveal a new layer of control in glycosylation: regulated intra-Golgi trafficking that tunes the retention or release of specific glycosyltransferases” was this actually shown in this work? I don’t recall seeing data for Golgi trafficking of GTs in the manuscript.
Figure 1C-D: Direction of change in volcano plot is not clear from labelling of volcano plots. Did the reduction of glycopeptides of Kng1, Apoc4, Lamp1 and Man1b1 in KOs lead to a relative higher level of the non-glycosylated form or other glycoforms that can support the observation and give clues to which part of the O-glycosylation machinery is impacted by GOLPH-3 disruption? The figure gives the impression that only T-glycopeptides have been compared whereas T and Tn were mentioned in the Methods, which is correct? Regardless, given the narrow view of the O-glycoproteome is there evidence for the following statement? “Several known GALNT2 substrates (e.g., Kng1, Apoc4, Lamp1) show reduced glycosylation in GOLPH3-/- livers.”. I suggest ‘…relative reduction in T antigens in GOLPH3-/- livers’ to be more specific.
Figure 1I-K: “Further sphingolipidomic profiling confirmed elevated GlcCer levels, while 131 downstream metabolites of LCS activity remained unchanged (Figure 1J). These findings indicate reduced LCS activity in the absence of GOLPH3, resulting in a metabolic bottleneck and GlcCer accumulation”. If this is the case, wouldn’t it be expected that the products of this enzymatic reaction also should be reduced?
Methods: “Methionine oxidation and HexNAc attachment to serine, threonine, and tyrosine were used as variable modifications for ETD MS2”. Was HexNAc the only glycan composition in the glycan search space? The manuscript mentions that both T and Tn antigens were analyzed while the figures indicate that only T antigens were plotted. Please describe more clearly how the glycoproteomics data was searched and plotted so this critical part can be reproduced and fully understood. Were any normalization steps (other than peptide amount in each channel) performed for the TMT reporter ions before plotting the data? As well as the raw (glyco)proteomics data, the authors are encouraged to provide lists of the identified proteins and (glyco)peptides in the SI to enable ready access to the underpinning data.
Minor: GalNac/GlcNac should be GalNAc and GlcNAc. Should ‘clients’ be ‘protein substrates’ or “binding partners”? Is ‘molecular logic’ the most accurate phrase?

Version 1:

Reviewer comments:

Reviewer #1

(Remarks to the Author)
I thank the authors for mostly addressing the reviewer comments. However, while the authors have addressed most of the comments, the quality of the responses and the corresponding revisions in the manuscript require revisions:

Previous Comment 1: "Mutants (C84A, C108A/C122A, etc.) are used in cell lines. It would strengthen the manuscript to demonstrate partial or full rescue of glycosylation/phenotypic defects in knockout mice or primary cells. This would establish direct physiological relevance of the regulatory mechanism."

Please include this answer and discussion in the main text including the relevant literature.

Previous Comment 2: "The different orientations depending on PIP4 lipid composition are interesting. How do these orientations compare with available web-servers that predict the GOLPH3 membrane orientation such as DREAMM, OPM, MODA, and PMIpred? The authors should present this comparison and
include it in Figure S3."

Thank you for adding this discussion in the main text, however, you did not properly cite the tools that you used: "OPM, DREAMM, and MODA". I also did not find a proper citation for AlphaFold3 as well as for many other previous works.
I urge the authors to carefully review the manuscript line by line and ensure that appropriate citations are included throughout. Given the length of this computational/experimental study, the current number of references (49) appears insufficient to properly acknowledge prior work and the state of the art.

Previous Comment 3: "What is the duration of each of the five independent replicas for each of the CG MD systems? It is not mentioned explicitly in the manuscript, but one can tell from Figure S3A that they have varying duration."

The sentence "(and run them in multiple replicas, each at least 2 μs long." is not sufficiently explaining what was done. What is the duration of each of the five independent replicas for each of the CG MD systems? Please add a table, explain why

simulations have different lengths, and include the relevant information in the manuscript and fix the several typos.

Previous Comment 7: "Terms such as “inactive state” and “alternative orientation” could be better defined for clarity."

The term “alternative orientation” remains in the text with no appropriate explanation with respect to the active and inactive state. Please clarify in the main text.

Reviewer #3

(Remarks to the Author)
The authors have adequately and satisfactorily addressed the major queries made by this reviewer (reviewer 3). The extra work done in response to these queries are noted and appreciated. There are some minor corrections to be made:
Minor Corrections (line numbers refer to the tracked changes version)
1. Line 1575: should the temperature be 308K rather than 318K.
2. Line 1579: replace semi-colon “;” with comma “,”
3. Line 1585: replace word “plugging” with “using” as plugging is colloquial
4. Line 1595: remove the word “too” in “in p.p.m too”

Reviewer #4

(Remarks to the Author)
The authors have done a good job responding to the points raised and their efforts have elevated the quality of the manuscript. However, a minor revision is required before I can move to recommend this work for publication.

Line 937: "In the liver, GOLPH3 deficiency causes a reduction of T/Tn-antigen–bearing O-glycopeptides and accumulation of GlcCer...". I still do not see any data for Tn as Figure 1D appears to be exclusively T glycopeptides. Please include Tn data.

Line 1221: "Raw data have been deposited to the ProteomeXchange Consortium 43 via the PRIDE partner repository with the data set identifier PXD010155." Is this the correct identifier for the generated (glyco)proteomics data for this study? Lipidomics and transcriptomics data should also be shared in line with community standards.

Open Access This Peer Review File is licensed under a Creative Commons Attribution 4.0 International License, which permits use, sharing, adaptation, distribution and reproduction in any medium or format, as long as you give appropriate credit to the original author(s) and the source, provide a link to the Creative Commons license, and indicate if changes were made.
In cases where reviewers are anonymous, credit should be given to 'Anonymous Referee' and the source.
The images or other third party material in this Peer Review File are included in the article's Creative Commons license, unless indicated otherwise in a credit line to the material. If material is not included in the article's Creative Commons license and your intended use is not permitted by statutory regulation or exceeds the permitted use, you will need to obtain permission directly from the copyright holder.
To view a copy of this license, visit https://creativecommons.org/licenses/by/4.0/

Reviewer #1

This manuscript addresses the physiological role and molecular regulation of GOLPH3, a Golgi adaptor implicated in retrograde trafficking of glycosyltransferases. Using a combination of mouse knockout models, glycoproteomics, lipidomics, structural biology (NMR, MD simulations), and cell-based assays, the authors reveal a dual S-acylation–mediated regulatory mechanism that governs GOLPH3's orientation and client-binding capacity. The study connects these mechanistic insights with in vivo effects such as glycosylation defects, embryonic lethality, etc. Overall, the work is well executed, using a multidisciplinary approach and is highly relevant to the niche fields of glycosylation and membrane trafficking. The study would be a useful addition in the literature provided that the following revisions are performed:

We thank this reviewer for the positive evaluation of our work.

Major Revisions

1. Mutants (C84A, C108A/C122A, etc.) are used in cell lines. It would strengthen the manuscript to demonstrate partial or full rescue of glycosylation/phenotypic defects in knockout mice or primary cells. This would establish direct physiological relevance of the regulatory mechanism.

We agree that demonstrating rescue of the glycosylation phenotype provides additional physiological validation of the proposed mechanism. However, performing such experiments in mice is currently not feasible because of major regulatory constraints. The GOLPH3 knockout line exhibits a severity that has led authorities to prohibit maintaining a homozygous ($GOLPH3^{-/-}$) colony. Given the low fertility rate of $GOLPH3^{+/-}$ mice and the non-Mendelian yield of $GOLPH3^{-/-}$ offspring from heterozygous crosses, the optimization process, and the number of conditions to test ($GOLPH3^{-/-}$; $GOLPH3^{-/-}$ + GOLPH3-WT; $GOLPH3^{-/-}$ + GOLPH3-C84A; $GOLPH3^{-/-}$ + GOLPH3-C108A/C122A), generating a sufficient number of biological replicates would require sacrificing several hundred mice to achieve statistical power.

*To address the reviewer's concern within feasible experimental limits, we instead performed experiments in GOLPH3 knockout HeLa expressing the various GOLPH3 mutants and considered their impact on glycosylation (here we used GSL as a model glycosylation pathway). These experiments show that the C84A mutant is less effective than wild-type GOLPH3 at promoting lipid glycosylation (here seen as Lc3 production), whereas the C108A/C122A mutant displays active behavior, consistent with our proposed dual S-acylation regulatory mechanism (now also displayed in **Figure 5D** and mentioned in the main text). These results provide additional evidence supporting the physiological relevance of the mechanism described.*

2. The different orientations depending on PIP4 lipid composition are interesting. How do these orientations compare with available web-servers that predict the GOLPH3 membrane orientation such as DREAMM, OPM, MODA, and PMIpred? The authors should present this comparison and include it in Figure S3.

We followed this recommendation and compared our experimentally and computationally derived GOLPH3 membrane orientations with predictions from publicly available servers, including OPM, DREAMM, and MODA (PMIpred could not be evaluated because it is primarily optimized for curvature-sensing proteins rather than general membrane binding). The OPM database already includes a precomputed orientation for GOLPH3 (OPM ID: 7958), and our own runs using the current PPM3 version of the OPM server, tested across different membrane types (undefined, plasma membrane, Golgi, and ER), consistently identified residues 80, 161, and 194–197 as membrane-interacting, with an identical predicted binding free energy of −8.7 $kcal{\cdot}mol^{-1}$. Predictions

from DREAMM and MODA were overall consistent with OPM regarding the membrane-binding interface. None of these tools captured the lipid-composition–dependent orientation shifts that we observe in our molecular dynamics simulations. We have included this comparative analysis in the revised manuscript (see new panel in ***Figure S3D****).*

3. The membrane composition states composition percentages, but it does not specify whether they are %mol, %v/v, %w/w. Please clarify.

We have now clarified in the Methods section that all membrane compositions used in the simulations are expressed as molar percentages (mol%).

4. What is the duration of each of the five independent replicas for each of the CG MD systems? It is not mentioned explicitly in the manuscript, but one can tell from Figure S3A that they have varying duration.

Each of the five independent coarse-grained MD replicas was run for at least 2 μs; trajectories were cropped at this duration because membrane-binding behavior had reached apparent convergence by that point. In Figure S3A some traces may appear slightly shorter; this is due to alignment of all trajectories at approximately 5 Å (for example, note the brief upward fluctuation for replica 3, orange, around 1.75 μs). We have clarified this in the revised manuscript.

5. The manuscript overall lacks report and discussion of the statistical variance, especially in the calculations. For example, all the CG results should report standard deviations from the 5 replicas (for example, standard deviations should be added in the following sentence: "GOLPH3 embedded ~5 Å deeper into the membrane and made ~20 546 % more contacts"), but also in any other places where quantitative results are presented.

Moreover, more detail on the statistical methods used for lipidomics/proteomics analyses (e.g., FDR correction, replicate number) would enhance transparency.

While we believe we have provided sufficient information for most simulations, we acknowledge that we did not do so specifically for the simulations comparing the coarse-grained LCS-GOLPH3 complex in membranes with and without palmitoylation on GOLPH3's Cys84. This was because our initial exploration of the complex on that occasion involved only single runs without replicas. We have now run two additional replicas for each state (with and without palmitoylation on Cys84), and the combined results from all three replicas reveal no statistically significant effect. Consequently, we have removed the previous note regarding the potential role of palmitoylation in stabilizing GOLPH3 deeper into the membrane. We have now incorporated detailed information regarding the statistical methods used for both lipidomics and proteomics analyses, including replicate numbers and false discovery rate (FDR) correction, throughout the revised manuscript to enhance transparency."

6. The connection between glycosylation defects and bone phenotypes could be elaborated, potentially referencing known glycosylation disorders with skeletal involvement. Moreover, a short discussion/comment could be added in the context of GOLPH3 being frequently overexpressed in cancer. The Discussion section could connect the regulatory model presented here with potential mechanisms underlying oncogenesis or altered glycomes in tumors.

We thank the reviewer for encouraging us to contextualize our findings. We have expanded the Discussion to address these points. First, regarding the link between glycosylation and bone phenotypes, we now explicitly reference congenital disorders of glycosylation (CDGs) known to present with skeletal abnormalities. More specifically, we highlight that loss-of-function in key GOLPH3 client enzymes, such as EXT1 and GALNT2, has been directly linked to skeletal defects, providing a

mechanistic rationale for the phenotypes observed in our knockout mice. We have integrated a discussion on the implications of our regulatory model for cancer. Building on our previous work (Rizzo et al., EMBO J 2021), where we established a causal link between GOLPH3 gain-of-function, increased glycosphingolipid (GSL) synthesis, and aberrant mTOR activation. While direct mutations of GOLPH3's palmitoylation sites are rare in tumors, we note that dysregulation of upstream acyltransferases (ZDHHC13/5) is common and could similarly distort the glycome. Furthermore, referencing recent literature, we now discuss how this GOLPH3-driven surge in GSL production may not only fuel proliferation but also facilitate immune evasion, positioning GOLPH3-mediated glycosylation as a dual driver of oncogenic fitness.

7. Terms such as "inactive state" and "alternative orientation" could be better defined for clarity.

In the context of our study, the terms "inactive state" and "active state" refer to the two distinct membrane orientations that GOLPH3 adopts depending on its S-acylation status. Specifically, these orientations correspond to conformations that either prevent or enable effective interaction with client proteins. We have now revised the manuscript to clarify and better articulate this nomenclature, ensuring that the distinction between orientation and functional state is clearly explained in the text.

Reviewer #2

The mechanism by which glycosylation enzymes are retained in distinct subcompartments of the Golgi is a longstanding question in cell biology. In this study, the authors generated GOLPH3 KO mice and report that GOLPH3 depletion disrupts both protein and lipid glycosylation, causes partially penetrant embryonic lethality, and severely impairs growth and bone mineralization. Using biophysical and biochemical approaches, they further show that GOLPH3 topology and membrane association are regulated by palmitoylation and PI4P levels, and that GOLPH3 binds the cytosolic tail of LCS (B4GALT5), which is required for its Golgi retention. Overall, the study is of high quality and significance, and the manuscript is clearly written. However, the evidence remains relatively limited to support the strong conclusion that GOLPH3 is a central regulator of Golgi enzyme retention.

We thank this reviewer for the thorough and positive evaluation of our work. We appreciate the comment regarding the strength of the evidence supporting GOLPH3 as a central regulator of Golgi enzyme retention. We have carefully considered this point and addressed the referee concerns as detailed below.

Major concerns:

1. The GOLPH3 knockout in mice appears incomplete. The manuscript does not explain what residual protein or fragments remain and how these might influence the phenotypes described. Relatedly, in Figure 3E, a band is still visible in the [^{3}H]-palmitate autoradiograph in the GOLPH3 KO lane, similar to that observed in the GOLPH3-5CA lane. The authors should clarify the identity of this band.

The reviewer is correct that the CRISPR-mediated knockout in mice excises the genomic region corresponding to exons 2 and 3 of GOLPH3. Exon 2 encodes amino acids 78-119 and exon 3 encodes amino acids 120–158, leaving open the possibility of a residual transcript encoding a truncated GOLPH3 protein missing 80 of the 298 residues in the wild-type protein. These residues span important functional regions of GOLPH3, including domains involved in membrane and PtdIns(4)P interactions and are important for maintaining overall protein structure.

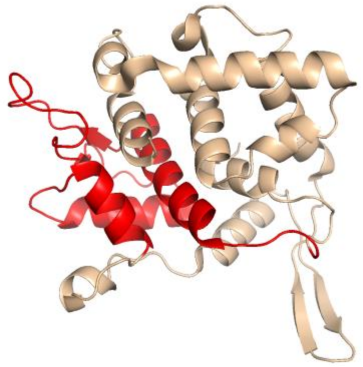

***PointbyPoint Figure 1**. In red here are residues corresponding to Exon2 and 3 excised by CRIPSR-CAS9 mice*

*By Western blot, we do not detect any specific GOLPH3 band in liver lysates from GOLPH3$^{-/-}$ mice; proteomic analysis, however, reveals a low-abundance GOLPH3-derived peptide (i.e., [R].AAGGGGGSGEDEAQSR.[R] aa 28-43) whose level is reduced by ~75% compared with wild type (**Figure 1C**). <u>This suggests that any truncated protein that is produced is both highly unstable and functionally compromised.</u>*

The HeLa knockout was generated using a GOLPH3 CRISPR/Cas9 KO plasmid that targets a 5' constitutive exon with three distinct 20-nt guide RNAs to ensure high knockout efficiency. No GOLPH3

*band is detected by Western blot, and no GOLPH3 signal is observed by immunofluorescence. Accordingly, the band seen in the [3H]-palmitate autoradiograph in the GOLPH3 KO lane (Fig. 3E) is unlikely to represent GOLPH3, since no protein of the expected molecular weight is detected in the corresponding Western blot (**Figure 3E, bottom**).*

We have clarified these points in the revised manuscript to make the nature of the mouse knockout and the interpretation of the residual signal explicit.

2. The evidence for GOLPH3's role in retaining Golgi enzymes is limited to LCS. Do other glycosyltransferases interact with GOLPH3, and is the interaction regulated by PI4P levels? Currently, the lack of broader analysis (e.g., co-IP or pulldown assays) weakens the general conclusion that GOLPH3 mediates retrograde trafficking of multiple Golgi-resident enzymes. In addition, could cell-surface biotinylation/streptavidin pulldown followed by MS analysis reveal which Golgi enzymes are mislocalized in GOLPH3 KO cells compared with WT cells?

*We apologize for not conveying this information clearly. The suggested experiments have in fact been previously performed by our group and independently by Sean Munro's team (see Rizzo et al., EMBO J 2021; Welch et al., JCB 2021), which show that GOLPH3 is a broad-spectrum COPI adaptor that interact with **>20 distinct glycosyltransferases** across multiple glycosylation pathways to mediate their Golgi retention. In addition to B4GALT5 (LCS), GOLPH3 has been shown to interact with and retain numerous enzymes, including ST3GAL5, A4GALT, ST8SIA1 and B4GALNT1 (**GSL pathway**); MAN1B1 and MGAT2 (**N-glycosylation**); C1GALT1, GALNT2, GALNT6, GALNT7 and GCNT1 (**mucin-type O-glycosylation**); EXT1 and FAM20B (**proteoglycan O-glycosylation**); POMGNT1 (**O-linked mannosylation**); PGAP4 (**GPI-anchor maturation**); TPST1 and TPST2 (**tyrosine sulfation**); the **orphan glycosyltransferases** GLT8D1 and FAM3C; and **other Golgi residents** such as CASC4, DIPK2A, GLG1, GOLIM4 and EBAG9.*

Wherever tested, Golgi retention of these clients depended on GOLPH3 PtdIns(4)P binding. Regarding functional assays (such as the surface surface-biotinylation assay suggested by the referee) it has to be noted that unlike the Golgi retention reporters used in this and previous studies (see Rizzo et al., EMBO J 2021; Welch et al., JCB 2021), full-length glycosyltransferases do not accumulate at the plasma membrane when not retained by GOLPH3 but are relocalized to lysosomes and degraded (see Rizzo et al., EMBO J 2021; Welch et al., JCB 2021). Accordingly, degradation assays and lysosomal localization studies have shown that loss of GOLPH3 leads to lysosomal relocalization and degradation of multiple Golgi resident enzymes (see Rizzo et al., EMBO J 2021; Welch et al., JCB 2021). We have now cited these published data and discussed the broader functional evidence supporting a general role for GOLPH3 in Golgi enzyme retention.

3. In the MD simulations, the authors conclude that PI4P is not strictly required for GOLPH3 membrane association, but modulates binding orientation in a concentration-dependent manner. Is there an experimental way to validate this prediction? For instance, what are the actual PI4P levels in the TGN in the relevant conditions? Where is ZDHHC13 localized within the Golgi, and how does palmitoylation interplay with PI4P levels to regulate GOLPH3 membrane association and enzyme interaction?

*Our MD simulations indicate that GOLPH3 possesses an intrinsic propensity to orient its PtdIns(4)P-binding site toward the membrane interface even in the absence of PtdIns(4)P, driven by hydrophobic interactions involving its beta-loop. However, the presence of physiological PtdIns(4)P levels (estimated at 1-5 mol% at the trans-Golgi / TGN[1]) stabilizes this configuration, as a GOLPH3 mutant unable to bind PtdIns(4)P (i.e., GOLPH3-R90L) shows defective Golgi binding (**Figure 3G, H** and **S5A**). We hypothesised that Cys84 S-acylation further stabilise this configuration by locking GOLPH3 to the Golgi membrane.*

To experimentally test this interplay, we assessed GOLPH3 localization under conditions where both regulatory inputs were perturbed. We treated cells expressing GOLPH3 S-acylation mutants with PIK93 (an inhibitor of PI4KIIIbeta) to deplete TGN PtdIns(4)P. Our data show that, compared to another TGN PtdIns(4)P-binding protein (CERT), GOLPH3 WT is significantly more resistant to PtdIns(4)P depletion, remaining Golgi-associated at PIK93 concentrations that completely relocate CERT to the cytosol. Conversely, mutation of the PtdIns(4)P-binding site (GOLPH3-R90L) largely abolishes Golgi localization, and its residual Golgi-associated fraction remains insensitive to PIK93. These data confirm the importance of PtdIns(4)P for initial Golgi recruitment, while they suggest that, once bound to Golgi, GOLPH3 does not strictly require PtdIns(4)P to persist on membranes likely because it gets stabilised there by S-acylation.

*Consistently, preventing S-acylation at Cys84 (GOLPH3-C84A) reduces Golgi localization and renders it sensitive to PIK93-induced PtdIns(4)P depletion, indicating that Cys84 acylation contributes to maintaining GOLPH3's Golgi association, especially when PtdIns(4)P is limiting. In stark contrast, mutating Cys108 and Cys122 resulted in a strongly Golgi-associated and Pik93-insensitive GOLPH3 form, highlighting the specific regulatory role of Cys84 (**Figure S5A**).*

*These data confirm that PtdIns(4)P binding and Cys84 S-acylation operate as a coincidence detection mechanism, likely functioning consecutively to orient and anchor GOLPH3 at the trans-Golgi. Supporting this spatial logic, our new super-resolution microscopy data (**Figure S5B**) demonstrate that ZDHHC13 (responsible for Cys84 S-acylation) is distributed across the Golgi stack but is significantly enriched at the trans-side. This places the acyltransferase precisely where it is needed to lock GOLPH3 into its PtdIns(4)P-bound, client-competent state.*

4. The link between LCS mislocalization and the developmental/growth defects observed in mice is insufficiently addressed. In addition to confirming that LCS is mislocalized in GOLPH3 KO mice, the authors should also provide a more direct explanation or mechanistic model for how loss of LCS retention leads to the complex in vivo phenotypes.

We again apologize for not making GOLPH3's broader physiological impact clear. As stated above GOLPH3 does not act solely through LCS: proteomic and functional data show GOLPH3 controls Golgi retention and stability of dozens of glycosyltransferases and Golgi residents. In this study LCS was used as an exemplary client to study the regulation of GOLPH3 with molecular resolution. Mechanistically, loss of GOLPH3 impairs PtdIns(4)P-dependent COPI recruitment, causing failure to retain multiple enzymes, rerouting them to endolysosomal compartments for degradation and reducing steady-state enzyme levels. The resulting combined deficits across N-, O-, glycosphingolipid, proteoglycan, GPI-anchor and sulfation pathways remodel the glycome in developing tissues, perturbing extracellular matrix composition, receptor signaling and cell–cell/ cell–matrix interactions that are critical for skeletal development and bone homeostasis. This cumulative glycosylation defect plausibly explains the complex developmental and growth phenotypes in mice and is consistent with human genetic evidence (see response to reviewer 1 n6). We have expanded the Discussion to present this mechanistic model, cite relevant literature linking disrupted glycosylation to skeletal abnormalities, and summarize the multiple pathways and client enzymes likely contributing to the in vivo phenotype.

Reviewer #3

Summary Comments on Paper:

The purpose of this paper is to understand the biological role of GOLPH3 in intra-Golgi trafficking. To achieve these aims, the manuscript used a variety of approaches, ranging from gene deletion of GOLPH3 in mice models, to detailed molecular delineation of the possible mechanisms of GOLPH3 function marrying computer simulations with detailed designed of experimental proofs of the results from these simulations; this combination of computer-aided studies with experimental proofs makes this paper interesting and is a good example of how these two approaches could be used in a complementary manner to make new discoveries. A very considerable amount of work is included in this paper with many groups involved. Much of the work were done in sufficient detail and with logical approaches to arrive at an interesting story.

The manuscript contributes well to increasing our knowledge of the importance of GOLPH3 in intra-Golgi trafficking through its abilities to simultaneously bind to Golgi membrane and cargo proteins in a structurally viable manner.

We thank the reviewer for their thoughtful and positive assessment. We appreciate the recognition of the multidisciplinary approach, combining mouse genetics, computational modeling, and targeted biochemical and cell-biological experiments, and agree that this integrated strategy strengthens the conclusions. We have revised the manuscript to clarify how these complementary lines of evidence converge on a model in which GOLPH3 couples PtdIns(4)P-dependent Golgi membrane binding with cargo recognition to mediate COPI-dependent intra-Golgi retention of multiple enzymes. We also expanded the Discussion to emphasize the physiological relevance of this mechanism for glycosylation pathways and development, and to better highlight the scope and limitations of our study.

Major General Comments

The manuscript requires revision to improve its clarity, especially in the discussion section; obviously there had been significant amount of work involving many groups from different disciplines. The discussion section does not do justice to the efforts expended in the arriving at the conclusions. There should be more links between the results obtained and how this work has advanced our understanding of biological function of GOLPH3, rather than making general statements most of which are not linked to the current studies.

We have now rewritten the Discussion to improve clarity and explicitly link our results to the biological functions of GOLPH3. The revised text highlights how each major finding (mouse genetics, proteomics, biochemical assays, and computational modeling) supports the proposed PtdIns(4)P and S-acylation regulated model of GOLPH3 mediated Golgi enzyme retention, and we more clearly discuss the physiological and disease relevant consequences of these mechanisms.

Two examples are:

Line 709: ". These modifications toggle GOLPH3 between an active, Golgi-localized, client-binding–competent state and an inactive, plasma membrane–bound state with inverted topology"- What makes the GOLPH3 "active" or "inactive"; why does binding to the Golgi make the protein active whereas binding to the plasma membrane make the protein inactive. Which specific results demonstrate this?

Our interpretation is guided by the membrane-orientation consequences of differential S-acylation: acylation at C84 promotes an orientation compatible with PtdIns(4)P engagement and presentation of the client-binding surface toward the Golgi membrane interface, enabling GOLPH3 to bind Golgi-localized clients (i.e., the "active" state). C84 acylation is mediated by Golgi-localized ZDHHCs (notably ZDHHC3 and ZDHHC13). By contrast, acylation at C108 and C122, mediated primarily by the

plasma-membrane-enriched enzyme ZDHHC5, forces an alternative membrane orientation that impairs PtdIns(4)P engagement and occludes the client-binding interface, rendering GOLPH3 unable to retain Golgi clients (the "inactive" state). We now explain this model more clearly in the text and link it to the experimental and localization evidence supporting each step (site-specific acylation, ZDHHC localization and specificity, and the correlation between acylation state, membrane binding orientation, client retention, and glycosylation outcome).

Line 717: "Our findings reveal a new layer of control in glycosylation" - How did the results show this? Need to refer back to the specific findings.

Controlling Golgi localization and client interactions, we hypothesize that GOLPH3's S-acylation directly alters cellular glycosylation capacity. To address this point experimentally, we measured glycosphingolipid content in HeLa cells expressing S-acylation mutants of GOLPH3 and found that the C84A mutant is less effective than wild-type GOLPH3 at rescuing the glycosylation phenotype, whereas the C108A/C122A mutant shows active behavior. These results support the proposed dual S-acylation regulatory mechanism and are now included and discussed in the revised manuscript.

More specific Comments on Specific Sections:

NMR Comments

1. Are there enough peaks in the 2D HSQC spectra to match the number of residues of GOLPH3? Please include number of resolved backbone amide peaks.

As detailed below, GOLPH3 samples are difficult to produce and unstable in solution over time. This prevents preparing highly concentrated samples and necessitates keeping experimental times as short as possible. In practice, we had to produce multiple preparations to achieve the reported backbone resonance assignments. Peak counting in the HSQC spectra is complicated by heterogeneous cross-peak intensities, with some barely above the noise, especially in titrations with the LCS peptide. In the long HSQC used for assignment, we count 243 cross-peaks. Approximately 10 pairs correspond to Asn/Gln side-chain NH2 signals, leaving about 220 resolved backbone amide cross-peaks. The construct contains 248 residues, including 8 prolines, yielding a theoretical maximum of 240 backbone correlations. Thus, roughly 20 residues are broadened beyond detection and/or severely overlapped. For a protein of this size, this is acceptable; however, given these limitations, the shorter HSQCs used in titrations show fewer intense, well-resolved cross-peaks suitable for tracking interactions, and many correlations are also lost in the 3D spectra required for assignments.

2. The NMR peaks do not seem to have similar intensities, especially signals from the folded region of the spectrum (most obvious in Figures S6 and S7 where there are fewer overlays of spectra making it more obvious that the peaks are not of similar intensities). These resonance intensities vary by considerable amounts to cause concern. This is particularly curious given that GOLPH3 does not have an unusual shape for its structured core that might give rise to unequal signal intensities. A contributory factors could be that under NMR conditions and concentrations (unlike verification using SECMALS conditions) some degree of aggregation or multimerization or even disulphide bond formation had occurred. Can the authors comment on this resonance variation and include a spectrum without overlays of with GOLPH3-WT or GOLPH3-4G in the supplementary.

We agree that the heterogeneity in peak intensities is concerning and appreciate the chance to clarify, consistent with the explanations above. GOLPH3 is challenging to produce and handle: it must be purified at room temperature (not on ice), requires ≥250 mM NaCl to remain soluble (near the upper

limit tolerated by our cryoprobe), cannot be concentrated beyond ~250–300 µM, and is unstable at higher (already at 318 K) or lower (even 298 K) temperatures under our NMR conditions. Accordingly, all NMR data are reported at 308 K, the optimized trade-off. These constraints limit sample lifetime and spectral quality and likely underlie the heterogeneous cross-peak intensities. Even the GOLPH3-4G construct, which behaved somewhat better, yielded imperfect spectra. The long HSQC used for assignment (308 K) provided our best data; shorter titration HSQCs required reduced acquisition times and thus show more peaks near the noise level, leaving fewer that can be reliably tracked. These practical limitations prevented complete assignments but still allowed us to identify resonances that shift upon titration.

To address the reviewer's request, We measured not just T_2 but also T_1, in order to get R_2/R_1 profiles and thus better assess the apparent molecular weight and oligomerization state of the protein in solution. Of note, we could record this data on a 320 uM sample prepared ad hoc, that happened to be amongst the best ^{15}N GOLPH3 samples we ever obtained, so this represents a "best case" scenario.

Here are the results of T_1 and T_2 fits to all backbone-looking crosspeaks of the HSQC spectrum (i.e. obvious indole, asparagine and glutamine sidechains not analyzed). Like in all our NMR studies on Golph3, the data was obtained at 308K. The total measurement time was of slightly over a day, and when we tried to record longer spectra the sample precipitated sometime within the next day, so we report the results on the first (day-long) dataset. We show labels only for the confidently assigned residues:

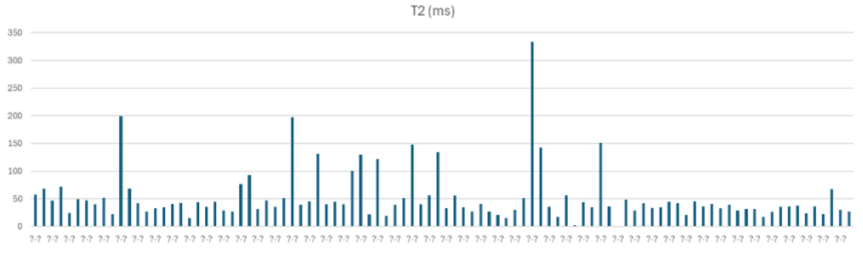

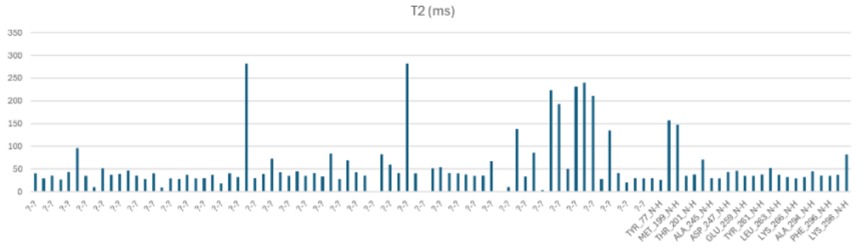

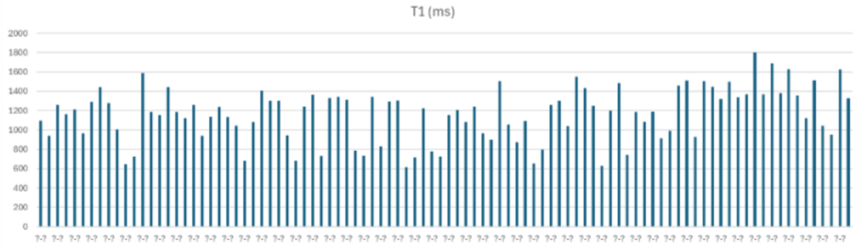

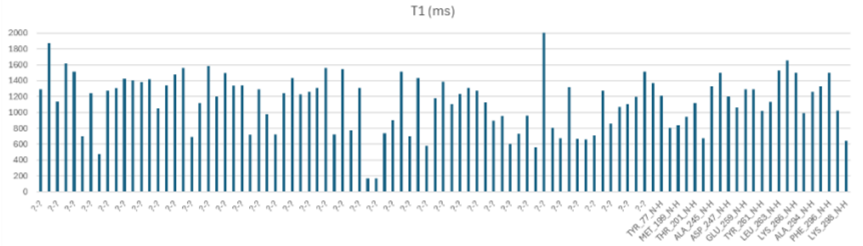

The average and median T_2 across all measurements are 51 and 38 ms, respectively; while the average and median T_2 across residues with confirmed assignments are 49 and 35 ms.

For T_1 the average and medians are 1150 and 1200 ms (all residues) and 1180 and 1200 ms (assigned residues).

These numbers yield central R_2/R_1 ratios in the range 28-31.5 (unitless). This corresponds to global correlation times (τ_c) in the range 12.6-13.4 ns according to the formula linking these values (Kay, L.E., Torchia, D.A. & Bax, A. Biochemistry 28, 8972-9 (1989)):

$$\tau_c = \frac{1}{4\pi\omega_N}\sqrt{6\frac{R_2}{R_1} - 7}$$

(where ω_N is the ^{15}N Larmor frequency, here around 80,000,000 Hz)

Calibration curves show linear correlations between τ_c and molar mass for folded globular proteins, for example [2]. Direct interpolation would suggest a molar mass of around 21 kDa, which is under the molar mass expected for monomeric Golph3 (28.2 kDa). However, these calibration data were obtained at 298K while one can expect a steep dependence with temperature because it affects rotational diffusion directly as well as through a decrease in viscosity:

$$\tau_c = \frac{4\pi\eta r^3}{3kT}$$

(where n is the solvent viscosity, that is lower at higher temperatures; k is Boltzmann's constant, r is the hydrodynamic radius of the protein, T is temperature in K)

Plugging values of water of 0.89 mPa s at 298K and 0.72 mPa s at 308K into the ratio of global correlation times at the two temperatures, we get:

$$\frac{\tau_{c,298K}}{\tau_{c,308K}} = \frac{\eta_{298K}308K}{\eta_{308K}298K} = \frac{0.89\ \ 308}{0.72\ \ 298} = 1.278$$

(viscosity data taken from https://www.engineersedge.com/)

From this ratio we get a global correlation time at 298 K in the range 16.2 – 17.2 ns. On calibration curves at 298 K, this means a molecular weight of 25.9-27.4 kDa, which fits much better with the molecular weight expected for the monomeric protein as prepared (28.2 kDa).

We finally show here a plot of the raw R_2/R_1 ratios for all crosspeaks analyzed, adding a central dashed red line close to its average and median value of around 30:

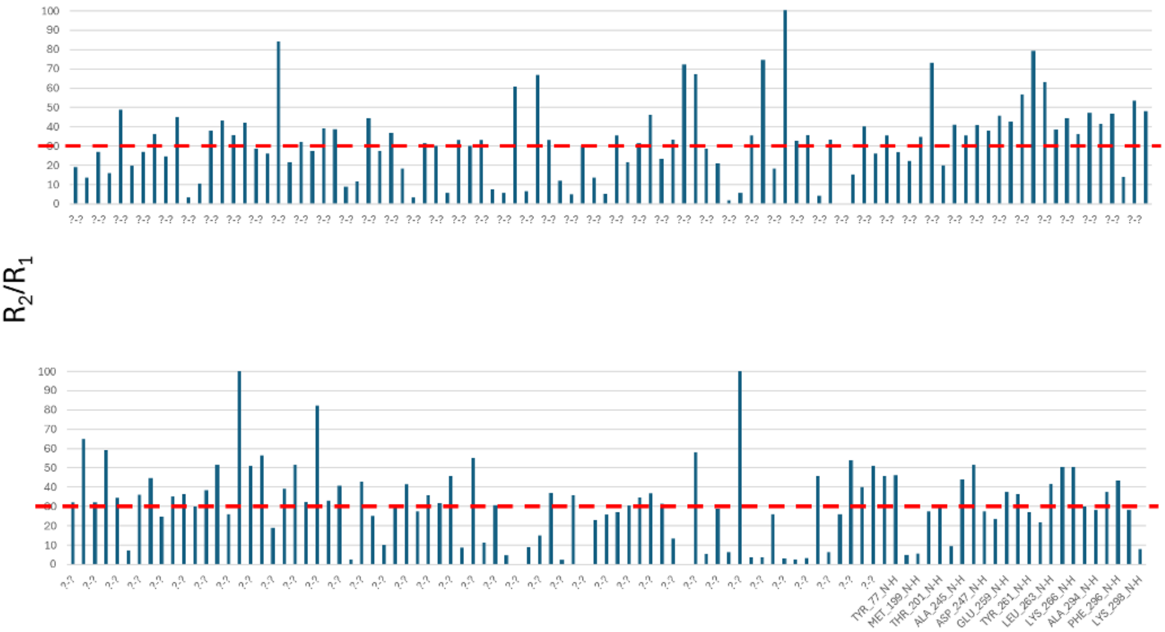

We note that although several crosspeaks display R2/R1 ratios around the central value of 30, there are also some 10% of crosspeaks with increased values which indicate residues undergoing either slow conformational exchange and/or reversible aggregation, both of which usually complicate assignment procedures. Besides, as stressed above this was a particularly good sample, and even being such, it wore off sometime after around 1.5-2 days of measurement (which is consistent with our need to utilize multiple samples for the 3D experiments that allowed us to achieve the presented assignments in the first place).

Conclusion: Overall, the best GOLPH3 preparations are largely monomeric while they remain stable, which means only a couple of days, and a fraction of residues undergo slow exchange even in the good sample.

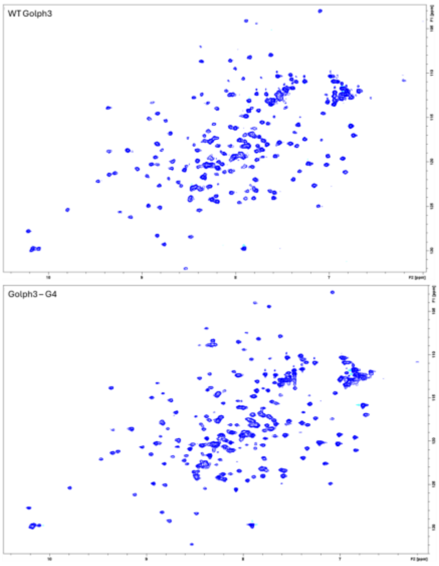

3. A quick T2 experiment could be carried out to verify that the NMR protein sample concurs with a monomeric form.

We have now done this, as detailed in the previous answer.

4.Using point mutants to confirm the assignments of a protein is a standard approach. However, the concern here is that mutation from D/E to A are nonconservative mutations carried out on residues that occur within structured helical regions of GOLPH3. There is a risk that structural changes could have occurred which alter the shifts of many other resonances (Lines 631 and 633, Fig S7B and C) and influence the robustness of using these point mutants to confirm the triple resonance assignments. Quantification of the number of peak perturbations in the different mutants should be presented in the supplementary.

We thank the reviewer for this important point. To quantify potential global structural effects of the point mutants we counted crosspeaks in the long HSQC used for assignment and compared them with the 243 peaks reported for WT (long HSQC). Peak counts for the mutants are as follows (note some samples were at lower concentration, which can affect visibility of weak peaks):

- *F30A (270 µM): 258 crosspeaks*
- *N32A (230 µM): 243 crosspeaks*
- *D208A (100 µM): 227 crosspeaks*
- *E209A (270 µM): 259 crosspeaks*

These counts show no evidence of large-scale loss of resonances for F30A, N32A or E209A, D208A measured at the lowest concentration displays a modest reduction in observable peaks consistent with weaker signal rather than wholesale unfolding. In addition, chemical shift perturbation patterns for the mutants are local and consistent with side-chain removal rather than widespread structural rearrangement (see main text Fig. S7 and residue-resolved CSP plots). We will include the above crosspeak counts and the corresponding per-residue CSP summaries in the Supplementary Information to make these controls explicit.

5. Interactions with LCS peptide:

(a) Summarise all the chemical shift perturbations with magnitude of changes.

*We have now added the CSPs to **Table S1***

(b) AlphaFold Multimer Model: Only one structure was presented as the only result but is this so? There are insufficient details given about the output statistics, how many poses were obtained, did they converged, how was the best pose selected, etc. This is a new generation of AI tool. Were others generative AI tools attempted? What model system was tried to verify the AF-Multimer approach?

All five models from the AlphaFold2-multimer run through ColabFold consistently predict interaction of the LCS with the interface identified by NMR, which is extensive and cannot be explained by a single docking pose, especially toward the C-terminal side of the peptide. However, taken together, all models cover this interface well. The pAE plots are largely red across the two proteins, but all show some white and blue (low pAE, hence higher confidence) at specific regions of the GOLPH3–LCS interface.

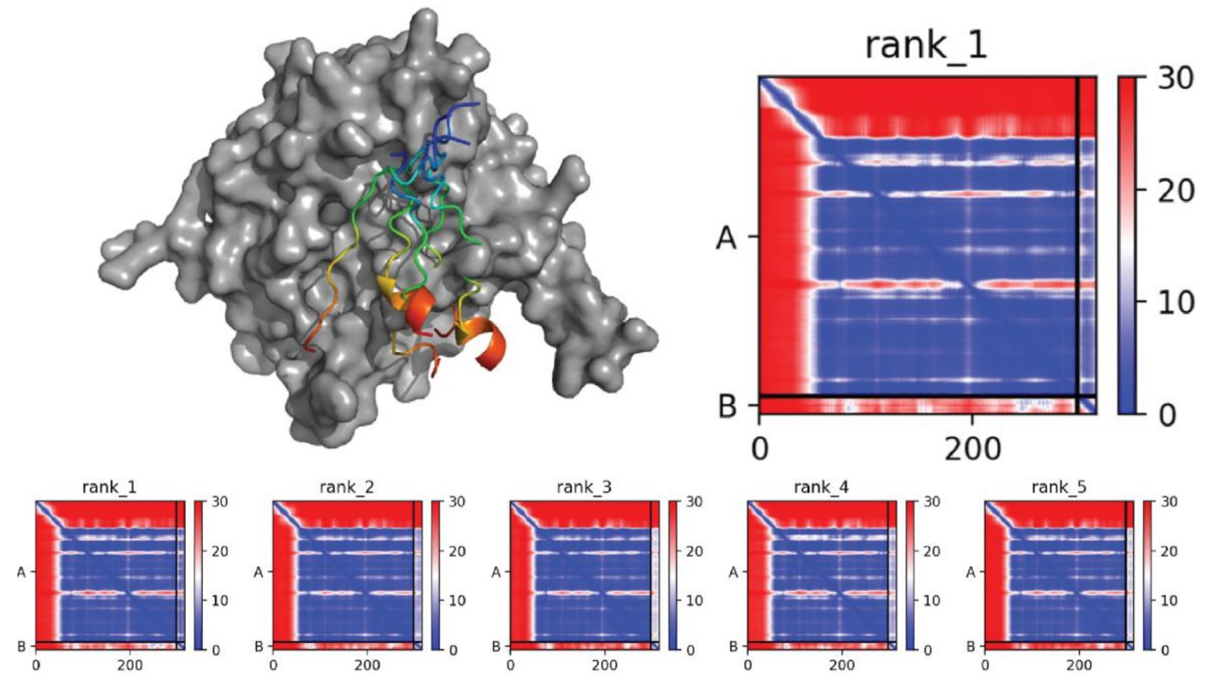

PointbyPoint Figure 2.

Top left: The five models of the GOLPH3–LCS complex predicted by AlphaFold2-multimer, with GOLPH3 shown as a grey surface and each of the five LCS poses shown as cartoons colored from blue at the N-terminus to red at the C-terminus.

Bottom: pAE plot for all five models from AlphaFold2-multimer, where the important scores are those crossing chains A (GOLPH3) and B (LCS peptide).

Top right: Enlarged view of the pAE plot for the model ranked #1, to better appreciate the shades of blue (low pAE) at specific points of the GOLPH3–LCS interface.

*In addition to the above reply, for this revision we also predicted the structure of the GOLPH3–LCS complex with 1,000 runs of AlphaFold 3 using different seeds. The resulting complexes are essentially the same as those obtained with AlphaFold2-multimer, but we have a larger sample and more informative scores (AlphaFold 3's scoring is more detailed). We now show one out of every ten snapshots from these 1,000 in Supplementary **Figure S8A** and **S8B**, with the latter illustrating in more detail the electrostatic and hydrophobic contacts observed in the AF3 model with the highest pTM score (0.89, reflecting global confidence) and highest ipTM score (0.53, specifically assessing the interface).*

Regarding cross-validation with experimental data: importantly, the predicted interface maps directly onto residues that shift in our NMR titrations (red sticks in the figures), providing experimental validation of the predicted binding surface. This concordance between NMR CSPs and recurring AF models is the main basis for our confidence in the modeled complex. Note that the NMR data were not used in any of the AlphaFold runs.

Moreover, ITC experiments using mutants of either LCS tail or of GOLPH3 indicate that residues predicted to partake in the interaction are indeed required for the binding. During the revision of this paper a new independent study has been published mapping GOLPH3 into the COPI complex[2] . A part of this study entails a mutational campaign on GOLPH3 to figure out which residues where relevant for client binding. Results reported in this study are convergent with ours substantiating oud findings.

Regarding alternative tools: beyond AF2-multimer, we performed extensive AF3 sampling (as described above) to broaden ensemble sampling. We did not include other protein–peptide AI docking tools in this round because, as described, <u>AF3/AF2-multimer provided a coherent, experimentally supported picture</u>.

siu(c) The structures shown in Figure 4C should be deposited in a public database such as Modelarchive.

We have now deposited the models as follows: the AlphaFold model in ModelArchive (ID ma-c5v3o) and the HADDOCK model in PDB-IHM (ID 9AAE). Because no single repository accepted both model types, we also submitted a unified Zenodo entry (doi:10.5281/zenodo.18430252) to keep them linked. In addition, we have deposited the partial backbone assignment for GOLPH3 in the BMRB (ID 53516).

6. Mutations of GOLPH3 for invitro Assays:

(a) it is not clear for the NMR, ITC, CD and interactions with LCS peptides whether site-directed mutants were performed on GOLPH3 or GOLPH3-4G. Please clarify – this is particularly important for understanding the effects of mutations on the melting temperatures.

We thank the reviewer for the request for clarification. D247A and D247A/D258A/D262A were generated on the wild-type (WT) GOLPH3 background.D247R and D247R/D258R/D262R were generated on the GGGG (4G) background. All NMR mutants were prepared on the GGGG (4G) background. We have added this information to the Methods and the figure legends for clarity.

(b) the mutations on the protein did produce measurable effects on the secondary structures of GOLPH3. As many of the point mutants were carried out on residues located in stable secondary structures, as well as the mutations were not conservative ones, some perturbations of protein secondary structures are expected and from the ellipticity calculations it is possible to quantify the extent of these perturbations, which in turn could affect the interpretation of some of the data; this should be done.

*We have now examined the secondary-structure content of all GOLPH3 mutants by circular dichroism and inspected HSQC spectra where available. The far-UV CD spectra of each mutant (including G4, D247A, D247R, DDD/AAA and DDD/RRR) essentially overlap in shape with the wild-type protein; deconvolution and ellipticity-ratio analysis (222/216, 222/208, 216/208) show no major, systematic changes in α-helix/β-sheet content (**Figure S9C,D** and **Table S2**). Likewise, the spread of cross-peak cross-sections in the 1H-15N HSQC spectra for the point mutants F30A, N32A, D208A and E209A is consistent with a similar folded state to wild type (no signs of global unfolding or major structural rearrangement). Together these data indicate that the mutations do not produce perturbations of secondary structure sufficient to affect interpretation of our functional results. We have added the CD spectra, ellipticity-ratio values, and a table of quantitative secondary-structure estimates to the Supplementary Information and included a brief statement to this effect in the Results.*

(c) Have point mutation effects on the secondary structures of LCS peptide been assessed either computationally or experimentally?

*We have now assessed LCS cytosolic tail secondary structure experimentally by circular dichroism (CD) and found no evidence of stable secondary structure for the full-length tail. Because some secondary-structure predictors returned marginal α-helical probabilities, we also performed CD in the presence of increasing concentrations of TFE (2,2,2-trifluoroethanol), which stabilizes α-helices by strengthening intramolecular H-bonds; even under these helix-promoting conditions we observed no helix signature. Truncation variants of the LCS tail were also tested by CD and likewise showed no detectable secondary structure (**Figure S7C, D**). These data are now included in the Supplementary Material.*

Line 366 and Figure 3: The data and description does not quantitatively address S-palmitoylation in the cells. All these data are showing is the presence of S-palmitoylation rather than the actual level of this post-translational modification. Secondly, this PTM is dynamic since S-

palmitoylation is reversible. Hence, these data and the description of the results is a snapshot of the level of PTM which could be quite small; this cannot be assessed from the current data since they are normalised to the WT level rather than an independent measure with, for example a quantifiable standard. Hence, the important and significance of the S-palmitoylation as an added mechanism in its role for GOLPH3 intra-Golgi trafficking need to be tempered.

We apologize for the insufficient explanation in the original text. We thank the reviewer for raising this point, which has allowed us to significantly improve this part of the manuscript.

Figure 3 presents three different assays to monitor S-acylation:

1. *Acyl-RAC, which captures S-acylated proteins and indicates whether a protein is S-acylated. While a snapshot of acylation, it does not provide information on specific sites or stoichiometry.*
2. *PEGylation, also a snapshot, offers more detailed information as every cysteine freed by hydroxylamine treatment is labeled with PEG. Figure 3D shows only a minimal band of GOLPH3 that has not undergone a band shift. This demonstrates that the vast majority of GOLPH3 is acylated on at least one cysteine at steady state. Quantification across multiple experiments is now provided.*
3. *3H-palmitate incorporation, which measures the signal incorporated into GOLPH3 during the pulse time, providing dynamic information rather than a snapshot. Comparisons between wild-type and mutant proteins indicate that 3H-palmitate incorporation is highly sensitive to the specific cysteine modified. Mutations of Cys84 almost abolish the signal, while mutations of Cys108 or Cys122 also affect S-palmitoylation. This suggests that all three cysteines can be modified, but acylation of Cys84 might be a prerequisite for the modification of the other two sites.*

*Briefly, in the PEGylation assay, free cysteines are first blocked, S-acyl thioesters are cleaved with hydroxylamine, and newly liberated cysteines are labeled with a maleimide-PEG reagent. Each PEG addition produces a discrete gel shift; thus, band intensities report the fraction of molecules bearing 0, 1, 2, or more modified cysteines. Quantification of these bands (now included in **Figure S4B**) shows that under our cellular conditions approximately **97% of GOLPH3 molecules are S-acylated on at least one cysteine** (approximately 54% mono-acylated, 30% di-acylated, and 13% tri-acylated). This demonstrates high steady-state occupancy rather than merely presence/absence.*

*The reviewer is entirely correct in highlighting that the modification is reversible. We have now addressed these dynamics. To probe turnover directly, we combined metabolic labeling with a clickable palmitate analogue followed by PEG-tagging ("click-PEG"). Silencing of ZDHHC5 or of ZDHHC3/13 (enzymes implicated in C108/C122 and C84 acylation, respectively) led to a near-complete loss of the click-PEG signal, consistent with these ZDHHCs being required for the observed acylation. Treatment with Palmostatin B or ML349 (an APT2 inhibitor), but not ML348 (an APT1 inhibitor) increased the click-PEG signal, indicating that APT-mediated deacylation (notably by APT2) does occur, suggesting ongoing cycles of acylation-deacylation (**Figure S4I**). Collectively, these observations support a model where the entire GOLPH3 population undergoes dynamic, sequential, and site-specific acylation (catalyzed by ZDHHC enzymes) and deacylation (by APT2).*
The Results section and the Discussion now include these data and a clear description of the various assays and their implications.

Line 366 and Figure 3E : What is the difference between lanes 1 and 3- they seem to be both from WT HeLa cells. Please clarify in figure legend.

We apologize for the lack of clarity in the figure legend.

- *Lane 1: WT HeLa cells.*
- *Lane 3: GOLPH3 KO HeLa cells re-expressing exogenously transfected WT GOLPH3.*

We have updated the figure legend in the revised manuscript to make this explicit.

Line 429: A rationale should also be given as to why the 5 cysteine residues are not S-palmitoylated to the same degree. Is this a structural or sequence or both issue?

Not all cysteines are S-acylation targets; in GOLPH3 we detect modification of three cysteines. Different ZDHHC enzymes target distinct cysteines, and their site specificity is encoded by the local sequence and folding around each Cys. General structural context also contributes: In this case, Cys84, Cys108 and Cys122 are more exposed at the protein surface than Cys92 and Cys280, and therefore are more likely to be modified (see attached figure). Nonetheless, this is only a first-approximation explanation: the mechanistic basis of S-acylation target specificity is an interesting question that extends beyond the scope of the current manuscript.

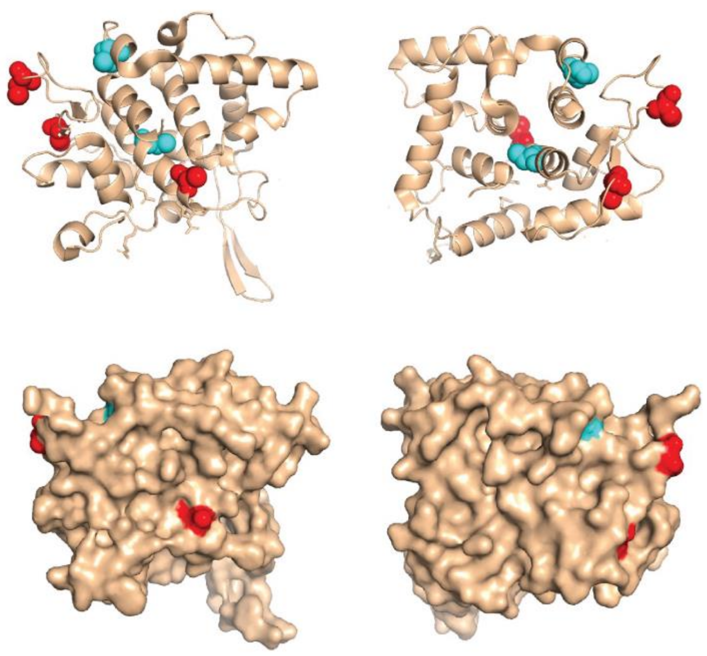

***PointbyPoint Figure 3**.*

Top: Cartoon representations (two different orientations) of GOLPH3 structure. Cysteines are shown as spheres. S-acylated cysteines are in red; non s-acylated cysteines are in cyan.

Bottom: Surface representation of the structures shown above.

Line 433-There is a need to have more details about the structure of GOLPH3 based on PDB 3KN1; although the crystal coordinates were released in 2009, a detailed description of the structure has not been published. At the very least some calculations of the electrostatic surface of GOLPH3 based in PDB 3KN1 should be presented both in the text and in the figures where references are made to the positive and negative surfaces of the protein.

*We have computed the electrostatic surface of GOLPH3 from PDB 3KN1 using the APBS server and mapped the potential onto the molecular surface (±kT/e color scale). The electrostatic properties are now shown in the revised **Figure 3D** and described in the Results, where we point out the positively charged patch and opposing negative surface referenced in the text.*

FigS5 Panel D should feature more prominently in the main figures rather than tucked away in FigureS5 since the positive and negative surfaces are important for the function of the protein and discussed much earlier in the text.

*Panel D from Figure S5 has been moved into a main figure (**Figure 3D**) in the revised manuscript so the positive and negative surface features are more prominently displayed and linked to the corresponding discussion.*

Line506: State the % sequence specific assignments achieved.

We achieved 11.3% sequence-specific backbone assignments (11.7% when prolines are excluded). this is now stated in the main text.

Line 427: Explain the functional significant of C108 and C122 being less S-acylated than C84.

We cannot conclude from our data that Cys108 and Cys122 are less S-acylated than Cys84. The [^{3}H]-palmitate incorporation assay primarily reports incorporation/turnover and can reflect slower acylation or slower turnover at particular sites rather than lower steady-state occupancy. Thus, differences observed could equally indicate altered kinetics (e.g., slower turnover) or sequential/site-dependent modification by distinct ZDHHCs. We now clarify this point and the associated caveats in the Results and Discussion.

Line 441: Explain the significance/rationale of ZDHHC13 specificity for C84 and ZDHHC5 for C108 and C122.

ZDHHC13 (and ZDHHC3) modify C84, whereas ZDHHC5 modifies C108/C122. Functionally, acylation at C84, installed by Golgi-localized ZDHHCs, favors a membrane orientation compatible with PI4P engagement and exposes the client-binding surface, thereby enabling GOLPH3 to retain Golgi enzymes (see also answer to reviewer 2 comment 3). In contrast, acylation at C108/C122 by the plasma-membrane-enriched ZDHHC5 promotes an alternative orientation that disrupts PI4P engagement and occludes the client-binding interface, rendering GOLPH3 non-conducive to enzyme retention. This is now better highlighted in the main text and in the discussion.

Reviewer #4 (Remarks to the Author):

The study explores molecular and cellular changes accompanying body-wide deletion of functional GOLPH3 in mice. Supported by various -omics analyses, an aspect this reviewer specifically was encouraged to look at, one conclusion is that disruption of GOLPH3 induces "glycosylation defects" both in terms of mucin-type O-glycosylation and GSL glycosylation. While such associations indeed appear to be supported, the manuscript does not well enough point out that evidence for a direct causal relationship between GOLPH3 dysfunction and altered glycosylation has not yet been provided. Conceivably, in addition to the involvement in retention and trafficking of GTs in the Golgi, GOLPH3 disruption may have unknown indirect functions that can't be disregarded such as for example by impacting the expression of nucleotide sugar transporters or glycosyltransferases/hydrolases or altering the glycosylation machinery in other unexpected ways through indirect pathways. As the glycoenzymes are often low abundant most of these would typically not be observed by proteomics and therefore require transcript level assessment.

*We agree that our current data do not yet prove a direct causal link between GOLPH3 loss and altered glycosylation, and that indirect mechanisms (for example, changes in expression of nucleotide-sugar transporters, glycosyltransferases/hydrolases, or other pathways) must be considered. To address this, we performed RNA-seq on WT and GOLPH3 KO livers. We found 58 significantly downregulated and 9 significantly upregulated genes ($p < 0.05$, $|FC| > 0.5$), but **inspection of a curated set of 238 glycogenes revealed no significant transcriptional changes** (**Figure S1D**). The transcriptomic shifts*

we observe instead reflect altered metabolism (downregulation of fatty-acid metabolic genes such as PNLIP, ELOVL2, FADS2, FABP2, consistent with TAG depletion) and a small set of upregulated genes indicating metabolic reprogramming (Aacs, Sult2a3, Cyp26b1), stress/inflammatory signaling (Txnip, Gbp2b), and increased DNA-replication/repair or cell-cycle activity (Lig1, Nup62cl). These changes are compatible with regenerative/proliferative responses secondary to liver injury and metabolic stress but do not explain the observed changes in protein and lipid glycosylation. Taken together, instead, the data support a model in which GOLPH3 loss perturbs glycosylation primarily via altered Golgi retention/stability of glycoenzymes, we have added the RNA-seq results to the revised manuscript (see ***Figure S1D****).*

Further, the statement that "loss of GOLPH3 disrupts glycosylation" is not accurate as the authors only presented data for the reduction of one or two form(s) of mucin-type glycosylation (T and/or Tn antigen) in GOLPH KOs, and thereby neither looked at if other glycoforms or the non-glycosylated forms were concomitantly increased nor identified which specific GTs were impacted by GOLPH ablation. Without such evidence, the conclusions should be better presented as "GOLPH3 loss appears to be associated with a global change in the protein and lipid glycosylation machinery albeit the specific glyco-enzymes driving these changes are yet to be established". Finally, "Our findings reveal a new layer of control in glycosylation: regulated intra-Golgi trafficking that tunes the retention or release of specific glycosyltransferases" was this actually shown in this work? I don't recall seeing data for Golgi trafficking of GTs in the manuscript.

We appreciate the reviewer's point regarding the specificity of our conclusions on glycosylation and the underlying trafficking mechanisms. We have revised the manuscript to address these points with greater precision.

First, we have tempered our language regarding the O-glycosylation data. Instead of stating broadly that "loss of GOLPH3 disrupts glycosylation," we now use the wording suggested by the referee. We explicitly acknowledge that while these changes are robust, the specific glyco-enzymes driving each alteration in the in vivo setting remain to be fully defined.

Regarding the reviewer's comment on intra-Golgi trafficking, we have enriched the Discussion by referencing our prior work (Rizzo et al., EMBO J 2021), where we demonstrated—in both mammals and yeast—that GOLPH3 drives the sorting of client enzymes into COPI vesicles, thereby mediating their recycling during cisternal maturation. This mechanistic role is further supported by recent structural data from Briggs and Munro (Science Advances 2005), which resolved GOLPH3 in complex with the COPI machinery, providing independent validation of its function in retrograde transport.

Crucially, this trafficking function explains the broad impact of GOLPH3 loss. Proteomic and functional studies identify >20 distinct Golgi residents as GOLPH3 interactors/clients, spanning GSL synthesis (e.g., B4GALT5), N-glycosylation (e.g., MAN1B1), and Mucin-type O-glycosylation (e.g., GALNT2). Consistent with this client diversity, depletion of GOLPH3 leads to widespread glycosylation defects across multiple pathways. Thus, while the fundamental role of GOLPH3 in intra-Golgi trafficking is well-established, our study adds a critical new dimension: ***regulation by S-acylation****. By demonstrating that reversible palmitoylation controls GOLPH3's membrane orientation and client engagement, we reveal S-acylation as a tunable "switch" for COPI sorting and enzyme recycling. It is in this specific sense that our findings "reveal a new layer of control in glycosylation," a concept we have now articulated more clearly in the revised Discussion.*

Figure 1C-D: Direction of change in volcano plot is not clear from labelling of volcano plots. Did the reduction of glycopeptides of Kng1, Apoc4, Lamp1 and Man1b1 in KOs lead to a relative higher level of the non-glycosylated form or other glycoforms that can support the observation

and give clues to which part of the O-glycosylation machinery is impacted by GOLPH-3 disruption? The figure gives the impression that only T-glycopeptides have been compared whereas T and Tn were mentioned in the Methods, which is correct? Regardless, given the narrow view of the O-glycoproteome is there evidence for the following statement? "Several known GALNT2 substrates (e.g., Kng1, Apoc4, Lamp1) show reduced glycosylation in GOLPH3-/- livers.". I suggest '…relative reduction in T antigens in GOLPH3-/- livers' to be more specific.

We have revised the text to be more precise and less generalizing. Specifically:

- *We have now clarified the direction of change in the legeld for the volcano plots.*
- *We now state that we used " lectin weak affinity chromatography (LWAC) followed by TMT-MS to enrich for T/Tn antigens O-glycopeptides"*
- *In the Discussion we now explicitly note that "broader glycoform profiling and dedicated studies are needed to assign which exact enzymatic steps are directly affected by GOLPH3 loss of function".*

These changes are incorporated in the revised manuscript and figure legend.

Figure 1I-K: "Further sphingolipidomic profiling confirmed elevated GlcCer levels, while 131 downstream metabolites of LCS activity remained unchanged (Figure 1J). These findings indicate reduced LCS activity in the absence of GOLPH3, resulting in a metabolic bottleneck and GlcCer accumulation". If this is the case, wouldn't it be expected that the products of this enzymatic reaction also should be reduced?

We thank the reviewer for this pertinent point. Lipid pool sizes reflect synthesis, turnover and interconversion, not instantaneous enzyme activity alone. In our mouse liver data (GSL lipidomics, proteomics and RNAseq) the marked increase in the immediate LCS substrate GlcCer together with comparatively smaller changes downstream provides evidence for an impaired flux through LCS in GOLPH3−/− livers. While the reviewer is correct that reduced LCS activity would, all else equal, tend to lower downstream product pools, multiple homeostatic and compensatory circuits in the GSL pathway can buffer or redistribute flux and product levels (see Russo et al., EMBO J. 2018; Capolupo et al., Science 2022). Thus, the elevated GlcCer: downstream-GSL ratio we observe is a sensitive indicator of a metabolic bottleneck at LCS even when absolute downstream GSL levels are not uniformly decreased.

Methods: "Methionine oxidation and HexNAc attachment to serine, threonine, and tyrosine were used as variable modifications for ETD MS2". Was HexNAc the only glycan composition in the glycan search space? The manuscript mentions that both T and Tn antigens were analyzed while the figures indicate that only T antigens were plotted. Please describe more clearly how the glycoproteomics data was searched and plotted so this critical part can be reproduced and fully understood. Were any normalization steps (other than peptide amount in each channel) performed for the TMT reporter ions before plotting the data? As well as the raw (glyco)proteomics data, the authors are encouraged to provide lists of the identified proteins and (glyco)peptides in the SI to enable ready access to the underpinning data.

We acknowledge that the initial description in the methods section inadvertently omitted the full glycan modification. This has now been corrected to accurately reflect our search parameters: "Methionine oxidation and Hex(1)HexNAc(1), HexNAc attachment to serine, threonine, and tyrosine were used as variable modifications for ETD MS2." This expanded search space indeed allowed for the identification of both T and Tn antigens, and the figures now reflect the appropriate data based on these identified glycopeptides.

Regarding the normalization of TMT reporter ion intensities prior to plotting, normalization and scaling were performed using the default settings of the Reporter Ions Quantifier Node (PD 2.2). Specifically, normalization was applied based on the total peptide amount, and the scaling mode utilized was "On All Average."
As suggested, a comprehensive list of the identified peptides and glycopeptides has now been included in the Supplementary Information to provide ready access to the underpinning data.

Minor: GalNac/GlcNac should be GalNAc and GlcNAc. Should 'clients' be 'protein substrates' or "binding partners"? Is 'molecular logic' the most accurate phrase?

We have amended the nomenclature to GalNAc and GlcNAc. We prefer to retain "client" (now defined at first use): in the GOLPH3 literature this term conveys a subtle distinction from simple "binding partner" (clients are Golgi enzymes whose localization/stability GOLPH3 helps maintain) and is more appropriate than "protein substrate," which implies enzymatic activity by GOLPH3.

1. Von Filseck, J. M., Vanni, S., Mesmin, B., Antonny, B. & Drin, G. A phosphatidylinositol-4-phosphate powered exchange mechanism to create a lipid gradient between membranes. *Nat Commun* **6**, 6671 (2015).
2. Taylor, R. J. *et al.* The mechanistic basis of cargo selection during Golgi maturation. *Sci. Adv.* **11**, eaea0016 (2025).

Reviewer #1 (Remarks to the Author):

I thank the authors for mostly addressing the reviewer comments. However, while the authors have addressed most of the comments, the quality of the responses and the corresponding revisions in the manuscript require revisions:

Previous Comment 1: "Mutants (C84A, C108A/C122A, etc.) are used in cell lines. It would strengthen the manuscript to demonstrate partial or full rescue of glycosylation/phenotypic defects in knockout mice or primary cells. This would establish direct physiological relevance of the regulatory mechanism."

Please include this answer and discussion in the main text including the relevant literature.

We have now added the following statements in the main text and in the discussion to cope with the criticism of this referee:

"While the physiological relevance of GOLPH3 S-acylation in regulating glycosylation and associated biological processes will require further in vivo investigations, our findings converge on a mechanistic model."

"Finally, our cellular studies demonstrate that GOLPH3 S-acylation is relevant for regulating glycosylation. Testing the role of GOLPH3 S-acylation on bone physiology directly in primary cells or in rescue experiments in knockout mice would further strengthen its relevance."

We believe this new text highlights the limitations of our approach. We do not see what additional literature needs to be cited here to sustain these statements.

Previous Comment 2: "The different orientations depending on PIP4 lipid composition are interesting. How do these orientations compare with available web-servers that predict the GOLPH3 membrane orientation such as DREAMM, OPM, MODA, and PMIpred? The authors should present this comparison and
include it in Figure S3."

Thank you for adding this discussion in the main text, however, you did not properly cite the tools that you used: "OPM, DREAMM, and MODA". I also did not find a proper citation for AlphaFold3 as well as for many other previous works.

I urge the authors to carefully review the manuscript line by line and ensure that appropriate citations are included throughout. Given the length of this computational/experimental study, the current number of references (49) appears insufficient to properly acknowledge prior work and the state of the art.

The referee is right. There was a problem with bibliography formatting. We have now fixed this and currently have 68 references. We have also added the relevant references for the tools we used.

Previous Comment 3: "What is the duration of each of the five independent replicas for each of the CG MD systems? It is not mentioned explicitly in the manuscript, but one can tell from Figure S3A that they have varying duration."

The sentence "(and run them in multiple replicas, each at least 2 μs long." is not sufficiently explaining what was done. What is the duration of each of the five independent replicas for each of the CG MD systems? Please add a table, explain why simulations have different lengths, and include the relevant information in the manuscript and fix the several typos.

We have corrected the text and updated the figure S3 to show the full simulation lengths. Specifically:

- *The five independent CG MD replicas have durations of 6.04 μs, 5.11 μs, 5.01 μs, 4.99 μs, and 5.17 μs, respectively. These values are now shown in the figure.*
- *The replica lengths slightly differ because simulations were terminated after stable membrane binding and equilibration were achieved in each run. Despite slight variation in total length, all replicas sample the same post-binding behavior: once the protein binds the membrane it remains bound for the remainder of each trajectory.*
- *In the original Figure S3A we only displayed the first 2 μs to avoid compressing the binding events at early times. However, to address your concern we have replaced that panel with a plot showing the entire duration of each replica; the full durations are also indicated on the time axis and in the legend.*

Previous Comment 7: "Terms such as “inactive state” and “alternative orientation” could be better defined for clarity."

The term “alternative orientation” remains in the text with no appropriate explanation with respect to the active and inactive state. Please clarify in the main text.

We have now changed nomenclature and refer to the “alternative orientation” as inverted orientation to stay closer to the data. The relationship between GOLPH3 orientation towards membranes and activity in retaining Golgi enzymes has been extensively discussed in the main text (see below). We do not think additional information is needed here.

“The functional consequences of these events are asymmetric. Cys84 lies on the β-hairpin/PtdIns(4)P-binding face and, when acylated, stabilizes the orientation in which this surface embeds into Golgi membranes and presents the client-binding groove at the membrane interface. Consistent with this, Cys84A mutants show reduced Golgi localization, heightened sensitivity to PI4KIIIβ inhibition (PIK93), and diminished ability to retain the LCS-SI-GFP reporter and to restore GSL biosynthesis in GOLPH3-KO cells. By contrast, Cys108 and Cys122 reside on the opposite side of the fold and are associated with the alternative, positively charged membrane-binding face. Their acylation correlates with reduced Golgi association and poorer retention activity, while C108A/C122A mutants are more strongly Golgi-localized, relatively insensitive to PIK93, and fully competent for LCS-SI-GFP retention and GSL rescue.

Together with the distinct localizations of ZDHHC13 (enriched at the trans-Golgi) and ZDHHC5 (broadly distributed), these data support a model in which: (i) initial Golgi recruitment of GOLPH3 is driven by β-hairpin insertion and PtdIns(4)P recognition; (ii) at the trans-Golgi, ZDHHC13/3 install Cys84 acylation, locking GOLPH3 into the PtdIns(4)P-bound, client-competent topology; and (iii) when GOLPH3 cycles to other membranes (e.g., plasma membrane), encounter with ZDHHC5 promotes Cys108/Cys122 acylation and engagement of the opposite face with PtdIns(4,5)P_2-rich

membranes, positioning the client-binding surface away from the membrane and functionally disengaging GOLPH3 from Golgi enzyme retention.

In this model, PtdIns(4)P binding and Cys84 S-acylation operate consecutively as a coincidence detector: both are needed to establish and maintain the active, Golgi-resident state. This is supported experimentally by the differential response of WT, R90L (PtdIns(4)P-binding deficient), C84A and C108A/C122A variants to PIK93. WT GOLPH3, once Golgi-bound, is relatively resistant to acute PtdIns(4)P depletion, implying that C84 acylation compensates when PtdIns(4)P is limiting. R90L fails to populate the Golgi properly and is largely insensitive to PIK93, as expected for a mutant that no longer depends on PtdIns(4)P. C84A becomes more dependent on PtdIns(4)P for Golgi association, whereas C108A/C122A shows strong Golgi residency even when PtdIns(4)P is reduced, consistent with a model where preventing "inactivating" acylation biases GOLPH3 toward the client-competent orientation."

Reviewer #3 (Remarks to the Author):

The authors have adequately and satisfactorily addressed the major queries made by this reviewer (reviewer 3). The extra work done in response to these queries are noted and appreciated. There are some minor corrections to be made:

Thank you

Minor Corrections (line numbers refer to the tracked changes version)
1. Line 1575: should the temperature be 308K rather than 318K.
done
2. Line 1579: replace semi-colon ";" with comma ","
done
3. Line 1585: replace word "plugging" with "using" as plugging is colloquial
done
4. Line 1595: remove the word "too" in "in p.p.m too"
done

Reviewer #4 (Remarks to the Author):

The authors have done a good job responding to the points raised and their efforts have elevated the quality of the manuscript. However, a minor revision is required before I can move to recommend this work for publication.

Line 937: "In the liver, GOLPH3 deficiency causes a reduction of T/Tn-antigen–bearing O-glycopeptides and accumulation of GlcCer...". I still do not see any data for Tn as Figure 1D appears to be exclusively T glycopeptides. Please include Tn data.

By design our LWAC approach does not discriminate between T and Tn antigen bearing peptides as Jacalin has affinity for both. The data we report should be interpreted as a measure of how much given peptides have been modified by GALNTs.

Line 1221: "Raw data have been deposited to the ProteomeXchange Consortium 43 via the PRIDE partner repository with the data set identifier PXD010155." Is this the correct

identifier for the generated (glyco)proteomics data for this study? Lipidomics and transcriptomics data should also be shared in line with community standards.

We have now made all data from proteomics lipidomics and transcriptomics available either in the form of supplementary files or as datasets submitted to public repositories.
